# Supplementary material for: Association between birth location and short-term outcomes for babies with gastroschisis, congenital diaphragmatic hernia and oesophageal fistula: a systematic review
Source: BMJ Paediatr Open. 2023 Jul 19;7(1):e002007. doi: 10.1136/bmjpo-2023-002007 (PMC10357737; doi:10.1136/bmjpo-2023-002007)
Supplement: Supplementary data [file bmjpo-2023-002007supp001.pdf]

Supplementary Material 1:

Table S1: Example Search Strategy

Search string	Database or further sources	Results	Date	Comments
((gastroschisis) OR (oesophageal atresia) OR (congenital diaphragmatic hernia)) AND ((location) OR (birth place) OR (site of delivery)) OR (transfer) OR (inborn) OR (outborn)	MEDLINE	192	2022-05-06	
MM "Gastroschisis") OR "gastroschisis" OR congenital diaphragmatic hernia OR congenital diaphragmal hernia OR tracheoesophageal fistula OR esophageal atresia AND ((location) OR (birth place) OR (site of delivery)) OR (transfer) OR (inborn) OR (outborn)	CINAHL	97	2022-05-06	
(((((ALL=(location)) OR ALL=(birthplace)) OR ALL=(site of delivery)) OR ALL=(inborn)) OR ALL=(outworn)) OR ALL=(transfer) AND (((ALL=(gastroschisis)) OR ALL=(congenital diaphragmatic hernia)) OR ALL=(tracheoesophageal fistula)) OR ALL=(esophageal atresia)	Web of Science	481	2022-05-06	
TITLE-ABS-KEY (((gastroschisis) OR (oesophageal AND atresia) OR (congenital AND diaphragmatic AND hernia)) AND ((location) OR (birth AND place) OR (site AND of AND delivery)) OR (transfer) OR (inborn) OR (outborn))	SCOPUS	529	2022-05-06	
((gastroschisis) OR (oesophageal atresia) OR (congenital diaphragmatic hernia)) AND ((location) OR (birth place) OR (site of delivery)) OR (transfer) OR (inborn) OR (outborn)	MEDLINE	10	2023-01-10	
(((((ALL=(location)) OR ALL=(birthplace)) OR ALL=(site of delivery)) OR ALL=(inborn)) OR ALL=(outworn)) OR ALL=(transfer) AND (((ALL=(gastroschisis)) OR ALL=(congenital diaphragmatic hernia)) OR ALL=(tracheoesophageal fistula)) OR ALL=(esophageal atresia)	Web of Science	35	2023-01-11	
TITLE-ABS-KEY (((gastroschisis) OR (oesophageal AND atresia) OR (congenital AND diaphragmatic AND hernia)) AND ((location) OR (birth AND place) OR (site AND of AND delivery)) OR (transfer) OR (inborn) OR (outborn))	SCOPUS	45	2023-01-11	
MM "Gastroschisis") OR "gastroschisis" OR congenital diaphragmatic hernia OR congenital diaphragmal hernia OR tracheoesophageal fistula OR esophageal atresia AND ((location) OR (birth place) OR (site of delivery)) OR (transfer) OR (inborn) OR (outborn)	CINAHL	9	2023-01-11	

Table S2: Excluded on Full Text Screening

Title	Author	Reason Exclusion
A single-center observational study on congenital diaphragmatic hernia: Outcome, predictors of mortality and experience from a tertiary perinatal center in Singapore.	Teo, Wan-Yee; Sriram, Bhavani; Alim, Aa Abdul; Ruan, Xucong; Rajadurai, V S	Details on whether transfer required? (Comparator);
Management and outcome of neonates with a prenatal diagnosis of esophageal atresia type A: A population-based study.	Garabedian, C; Bonnard, A; Rousseau, V; Sfeir, R; CRACMO; Drumez, E; Michaud, L; Gottrand, F; Houfflin-Debarge, V	Details on whether transfer required? (Comparator);
Survival of outborns with congenital diaphragmatic hernia: the role of protective ventilation, early presentation and transport distance: a retrospective cohort study.	Bojanic, Katarina; Pritisanac, Ena; Luetic, Tomislav; Vukovic, Jurica; Sprung, Juraj; Weingarten, Toby N; Carey, William A; Schroeder, Darrell R; Grizelj, Ruza	Details on whether transfer required? (Comparator);
[Influence of selected factors on the treatment and prognosis in newborns with gastroschisis on the basis of own experience].	Sawicka, Ewa; Wieprzowski, Lukasz; Jaczynska, Renata; Maciejewski, Tomasz	Details on whether transfer required? (Comparator);
Effect of time to surgical evaluation on the outcomes of infants with gastroschisis.	Bucher, Brian T; Mazotas, Ioanna G; Warner, Brad W; Saito, Jacqueline M	Details on whether transfer required? (Comparator);
Gastroschisis: a multi-centre comparison of management and outcome.	Manson, Joanna; Ameh, Emmanuel; Canvassar, Noel; Chen, Tiffany; den Hoeve, A Van; Lever, F; Hesse, Afua; Millar, Alastair; Emil, Sherif; Ade-Ajayi, Niyi	Details on whether transfer required? (Comparator);
[Congenital gastroschisis--prenatal diagnosis and perinatal management].	Weichert, J; Kahl, F O; Schroer, A; Bohlmann, M K; Diedrich, K; Hartge, D R	Details on whether transfer required? (Comparator);
Management and outcome for babies born with a gastroschisis.	Williamson, Sarah Louise; Lawrence, Louise; Arul, G. Suren; Rasiah, Shree Vishna	Details on exposure?

Study of 24 cases with congenital esophageal atresia: what are the risk factors?	Sugito K; Koshinaga T; Hoshino M; Inoue M; Goto H; Ikeda T; Hagiwara N	Details on whether transfer required? (Comparator);
A 20-year experience on neonatal extracorporeal membrane oxygenation in a referral center.	Schaible T; Hermle D; Loersch F; Demirakca S; Reinshagen K; Varnholt V; Schaible, T; Hermle, D; Loersch, F; Demirakca, S; Reinshagen, K; Varnholt, V	Details on whether transfer required? (Comparator);
Predictive factors for complications in children with esophageal atresia and tracheoesophageal fistula	Shah, R; Varjavandi, V; Krishnan, U	Details on whether transfer required? (Comparator);
Congenital diaphragmatic hernia - Results of an ECMO-centre	Dahlheim, M; Witsch, M; Demirakca, S; Lorenz, C; Schaible, T	Details on whether transfer required? (Comparator);
Congenital diaphragmatic hernia: A local experience	Khawahur, H; Kattan, A; Al-Alaiyan, S; Saidy, K	Details on whether transfer required? (Comparator);
Congenital diaphragmatic hernia: a modern day approach	Waag, KL; Loff, S; Zahn, K; Ali, M; Hien, S; Kratz, M; Neff, W; Schaffeder, R; Schaible, T	Details on whether transfer required? (Comparator);
Outcomes of Congenital Diaphragmatic Hernia: An 8-Year Experience	Fallahi, M; Mohajerzadeh, L; Borhani, S; Kazemian, M; Roozroukh, M; Khaleghnejad-Tabari, A; Azma, R; Mahdavi, A	Details on whether transfer required? (Comparator);
Outcomes of newborns with gastroschisis: The effects of mode of delivery, site of delivery, and interval from birth to surgery	Quirk, JG; Fortney, J; Collins, HB; West, J; Hassad, SJ; Wagner, C	
Congenital diaphragmatic hernia - a Belgrade single center experience	Kalanj, J; Salevic, P; Rsovac, S; Medjo, B; Antunovic, SS; Simic, D	Details on whether transfer required? (Comparator);
Congenital diaphragmatic hernia: a survey of practice in Scandinavia	Skari, H; Bjornland, K; Frenckner, B; Friberg, LG; Heikkinen, M; Hurme, T; Loe, B; Mollerlokken, G; Nielsen, OH; Qvist, N; Rintala, R; Sandgren, K; Serlo, W; Wagner, K; Wester, T; Emblem, R	Details on whether transfer required? (Comparator);
Survival of outborns with congenital diaphragmatic hernia: the role of protective ventilation, early presentation and transport distance: a retrospective cohort study	Bojanic, K; Pritisanac, E; Luetic, T; Vukovic, J; Sprung, J; Weingarten, TN; Carey, WA; Schroeder, DR; Grizelj, R	Details on whether transfer required? (Comparator);
A population-based study of congenital diaphragmatic hernia: Impact of associated anomalies and preoperative blood gases on survival	Kaiser, JR; Rosenfeld, CR	Details on whether transfer required? (Comparator);
Outcomes of congenital diaphragmatic hernia: A population-based study in western Australia	Colvin, J; Bower, C; Dickinson, JE; Sokol, J	Details on whether transfer required? (Comparator);
Outcomes in the physiologically most severe congenital diaphragmatic hernia (CDH) patients: Whom should we treat?	Kays, DW; Islam, S; Perkins, JM; Larson, SD; Taylor, JA; Talbert, JL	Details on whether transfer required? (Comparator);
Congenital diaphragmatic hernia: outcome review of 2,173 surgical repairs in US infants	Abdullah, F; Zhang, YY; Sciortino, C; Camp, M; Gabre-Kidan, A; Price, MR; Chang, DC	Details on whether transfer required? (Comparator);
Congenital Diaphragmatic Hernia Defect Size and Infant Morbidity at Discharge	Putnam, LR; Harting, MT; Tsao, K; Morini, F; Yoder, BA; Luco, M; Lally, PA; Lally, KP	Details on whether transfer required? (Comparator);
Analysis of 29 consecutive thoracoscopic repairs of congenital diaphragmatic hernia in neonates compared to historical controls	Cho, SD; Krishnaswami, S; Mckee, JC; Zallen, G; Silen, ML; Bliss, DW	Details on whether transfer required? (Comparator);
CONGENITAL DIAPHRAGMATIC-HERNIA - UPDATE ON REGIONAL EXPERIENCE	ALUMRAN, K; KHAWAJA, S; DAWODU, AH; ALARFJ, A	Details on whether transfer required? (Comparator);
IS SPECIALIST CENTER DELIVERY OF GASTROSCHISIS BENEFICIAL	NICHOLLS, G; UPADHYAYA, V; GORNALL, P; BUICK, RG; CORKERY, JJ	Details on whether transfer required? (Comparator);
Practice variation in gastroschisis: Factors influencing closure technique	Stanger, J; Mohajerani, N; Skarsgard, ED	Details on whether transfer required? (Comparator);
Delivery in a tertiary Center with co-located surgical facilities makes the difference among neonates with prenatally diagnosed major abnormalities	Calisti, A; Oriolo, L; Giannino, G; Spagnol, L; Molle, P; Buffone, EL; Donadio, C	Known birth location and whether surgical services are available on site (Intervention/Exposure)
INFLUENCE OF PLACE OF DELIVERY ON OUTCOME IN BABIES WITH GASTROSCHISIS	STOODLEY, N; SHARMA, A; NOBLETT, H; JAMES, D	Known birth location and whether surgical services are available on site (Intervention/Exposure)
Gastroschisis outcomes and site of delivery	Robilio, D; Greve, L; Towner, D	Known birth location and whether surgical services are available on site

		(Intervention/Exposure)
Mode of delivery and neonatal survival of infants with gastroschisis in Australia and New Zealand	Abdel-Latif, ME; Bolisetty, S; Abeywardana, S; Lui, K	Details on whether transfer required? (Comparator);
Factors Associated with Mortality in Neonates with Gastroschisis	Clark, RH; Walker, MW; Gauderer, MWL	Known birth location and whether surgical services are available on site (Intervention/Exposure)
Perinatal demography of gastroschisis in North Queensland	Whitehall, JS; Kandasamy, Y; Stalewski, H; Gill, A	Details on whether transfer required? (Comparator);
An Investigation of Racial-Ethnic Disparities in Location of Delivery, Hospital Transfer, and Survival Among Infants with Congenital Diaphragmatic Hernia	Carmichael, SL; Ma, C; Shaw, GM; Sylvester, KG; Hintz, SR	Known birth location and whether surgical services are available on site (Intervention/Exposure)
Gastroschisis and exomphalos in Ireland 1998-2004. Does antenatal diagnosis impact on outcome?	Murphy, FL; Mazlan, TA; Tarheen, F; Corbally, MT; Puri, P	Details on whether transfer required? (Comparator);
Trends in incidence and outcomes of gastroschisis in the United States: analysis of the national inpatient sample 2010-2014	Bhatt, P; Lekshminarayanan, A; Donda, K; Dapaah-Siakwan, F; Thakkar, B; Parat, S; Chabra, S; Billimoria, Z	Details on whether transfer required? (Comparator);
Complications of esophageal strictures dilatation in children A tertiary-center experience	Bawazir, O; Almaimani, MO	Details on whether transfer required? (Comparator);
The Effect of Level of Care on Gastroschisis Outcomes	Apfeld, JC; Kastenberg, ZJ; Sylvester, KG; Lee, HC	Details on whether transfer required? (Comparator);
Outcomes of newborns with gastroschisis: The effects of mode of delivery, site of delivery, and interval from birth to surgery - Discussion	Niebyl, JR; Woods, JR; Evans, MI; Queenan, JT; Carpenter, MW; Merkatz, IR; Quirk	Details on whether transfer required? (Comparator);
CONTROVERSIES IN THE MANAGEMENT OF GASTROSCHISIS - A STUDY OF 40 PATIENTS	STRINGER, MD; BRERETON, RJ; WRIGHT, VM	Details on whether transfer required? (Comparator);
Length of stay and cost analysis of neonates undergoing surgery at a tertiary neonatal unit in England	Shetty, S; Kennea, N; Desai, P; Giuliani, S; Richards, J	Details on whether transfer required? (Comparator);
Considerations for delivery of infants with congenital abnormalities	Wilkins-Haug, L	Details on whether transfer required? (Comparator);
Timing and location of delivery of the fetus with gastroschisis	Leuthner, SR; Wigton, T; Brown, AM; Sweeney, B; Sato, T; Aiken, J	Details on whether transfer required? (Comparator);
A systematic review and meta-analysis of the impact of location of delivery on outcomes in infants with gastroschisis	Nitsche, JF; Li, Y; Brost, B	Details on whether transfer required? (Comparator);
Prenatal diagnosis and the pediatric surgeon: The impact of prenatal consultation on perinatal management	Crombleholme, TM; DAlton, M; Cendron, M; Alman, B; Goldberg, MD; Klauber, GT; Cohen, A; Heilman, C; Lewis, M; Harris, BH	Details on whether transfer required? (Comparator);
Transfer and treatment mechanism and practical effect analysis of prenatally diagnosed congenital dia-phragmatic hernia	Ying, W.; Lishan, M.; Chao, L.; Yandong, W.; Jingna, L.; Yunlong, Z.; Yanxia, Z.; Rui, Z.	Details on whether transfer required? (Comparator);
The financial burden of surgery for congenital malformations—the austrian perspective	Gasparella, P.; Singer, G.; Kienesberger, B.; Arneitz, C.; Fülöp, G.; Castellani, C.; Till, H.; Schalamon, J.	Details on whether transfer required? (Comparator);
International recommendations on neonatal management with congenital diaphragmatic hernia: literature review	Babintseva, A.G.; Godovanets, Yu.D.; Khodzinska, Yu.Yu.; Popeliuk, N.O.; Koshurba, I.V.	Details on whether transfer required? (Comparator);
Clinical experience of ex-utero intrapartum treatment and prognostic factors of neonatal diaphragmatic hernia	Yunlong, Z.; Lishuang, M.; Yidin, J.; Ying, W.; Chao, L.; Yandong, W.; Jingna, L.; Yan, L.; Shan, J.; Hua, M.; Yunshu, O.; Feng, F.; Huf, Y.; Lijian, P.	Details on whether transfer required? (Comparator);
Effect of multidisciplinary treatment on outcomes in infants with congenital diaphragmatic hernia: 10-year experience	Pan, W.; Wang, W.; Wang, J.; Xie, W.; Wang, Y.; Jiang, Y.	Details on whether transfer required? (Comparator);
Prenatal diagnoses and outcomes of congenital diaphragmatic hernia.	Laye, M.R.; Rehberg, J.F.; Kosek, M.A.; Bufkin, L.K.; Bofill, J.A.	Details on whether transfer required? (Comparator);
Risk factors for mortality of newborns with gastroschisis in a tertiary hospital of Taiwan	Zhao, L.-L.; Chang, C.-J.; Chou, H.-C.; Tsao, P.-N.; Hsieh, W.-S.	Details on whether transfer required? (Comparator);
Gastroschisis: Determinants of neonatal outcome	Singh, S.J.; Fraser, A.; Leditschke, J.F.; Spence, K.; Kimble, R.; Dalby-Payne, J.;	Details on whether transfer required? (Comparator);

	Baskaranathan, S.; Barr, P.; Halliday, R.; Badawi, N.; Peat, J.K.; Glasson, M.; Cass, D.	
Congenital diaphragmatic hernia. Prenatal diagnosis and neonatal outcome	Lamberti, A.; Liguori, M.; Teodoro, A.; Tartaglione, A.; Caccioppoli, U.; Paladini, D.	Known birth location and whether surgical services are available on site (Intervention/Exposure)
Current status of antenatal diagnosis & perinatal medical network	Suita, S.	Details on whether transfer required? (Comparator);
Exomphalos and gastroschisis, a study of fetal and neonatal outcome in Wales 1974-1987	Evans, J.; Madarikan, B.A.; Lari, J.	Details on whether transfer required? (Comparator);
Anterior abdominal wall defects	Lafferty, P.M.; Emmerson, A.J.; Fleming, P.J.; Frank, J.D.; Noblett, H.R.	Known birth location and whether surgical services are available on site (Intervention/Exposure)
Development of a program for planned cesarean delivery and immediate pediatric surgical repair of ventral wall defects	Evans, M.I.; Drugan, A.; Greenholz, S.K.; Hauff, N.; Jewell, M.R.; Klein, M.D.	Details on whether transfer required? (Comparator);
Congenital diaphragmatic hernia: a 20 year experience	Simson, J.N.L.; Eckstein, H.B.	Details on whether transfer required? (Comparator);
Prenatal diagnosis and natural history of the fetus with a congenital diaphragmatic hernia: Initial clinical experience	Nakayama, D.K.; Harrison, M.R.; Chinn, D.H.; Callen, P.W.; Filly, R.A.; Golbus, M.S.; De Lorimier, A.A.	Known birth location and whether surgical services are available on site (Intervention/Exposure)
Perinatal management of gastroschisis	Fitzsimmons, J.; Nyberg, D. A.; Cyr, D. R.; Hatch, E.	Known birth location and whether surgical services are available on site (Intervention/Exposure)
Clinical profile, outcomes and predictors of mortality in neonates operated for gastrointestinal anomalies in a tertiary neonatal care unit- An observational study	Jerry, A.L.; Amboiram, P.; Balakrishnan, U.; Chandrasekaran, A.; Agarwal, P.; Devi, U.	Known birth location and whether surgical services are available on site (Intervention/Exposure)
Prenatal diagnoses and outcomes of congenital diaphragmatic hernia.	Laye, M.R.; Rehberg, J.F.; Kosek, M.A.; Bufkin, L.K.; Bofill, J.A.	Known birth location and whether surgical services are available on site (Intervention/Exposure)
Risk factors for mortality of newborns with gastroschisis in a tertiary hospital of Taiwan	Zhao, L.-L.; Chang, C.-J.; Chou, H.-C.; Tsao, P.-N.; Hsieh, W.-S.	Known birth location and whether surgical services are available on site (Intervention/Exposure)
Risk of need for extracorporeal membrane oxygenation support in neonates with congenital diaphragmatic hernia treated with inhaled nitric oxide	Sebald, M.; Friedlich, P.; Burns, C.; Stein, J.; Noori, S.; Ramanathan, R.; Seri, I.	Known birth location and whether surgical services are available on site (Intervention/Exposure)
Gastroschisis: Determinants of neonatal outcome	Singh, S.J.; Fraser, A.; Leditschke, J.F.; Spence, K.; Kimble, R.; Dalby-Payne, J.; Baskaranathan, S.; Barr, P.; Halliday, R.; Badawi, N.; Peat, J.K.; Glasson, M.; Cass, D.	Known birth location and whether surgical services are available on site (Intervention/Exposure)
Congenital diaphragmatic hernia. Prenatal diagnosis and neonatal outcome	Lamberti, A.; Liguori, M.; Teodoro, A.; Tartaglione, A.; Caccioppoli, U.; Paladini, D.	Known birth location and whether surgical services are available on site (Intervention/Exposure)
Current status of antenatal diagnosis & perinatal medical network	Suita, S.	Details on whether transfer required? (Comparator);
Exomphalos and gastroschisis, a study of fetal and neonatal outcome in Wales 1974-1987	Evans, J.; Madarikan, B.A.; Lari, J.	Details on whether transfer required? (Comparator);
Anterior abdominal wall defects	Lafferty, P.M.; Emmerson, A.J.; Fleming, P.J.; Frank, J.D.; Noblett, H.R.	Known birth location and whether surgical services are available on site (Intervention/Exposure)
Development of a program for planned cesarean delivery and immediate pediatric surgical repair of ventral wall defects	Evans, M.I.; Drugan, A.; Greenholz, S.K.; Hauff, N.; Jewell, M.R.; Klein, M.D.	Details on whether transfer required? (Comparator);
Congenital diaphragmatic hernia: a 20 year experience	Simson, J.N.L.; Eckstein, H.B.	Details on whether transfer required? (Comparator);
Prenatal diagnosis and natural history of the fetus with a congenital diaphragmatic hernia: Initial clinical experience	Nakayama, D.K.; Harrison, M.R.; Chinn, D.H.; Callen, P.W.; Filly, R.A.; Golbus, M.S.; De Lorimier, A.A.	Details on whether transfer required? (Comparator);
Perinatal management of gastroschisis	Fitzsimmons, J.; Nyberg, D. A.; Cyr, D. R.; Hatch, E.	Details on whether transfer required? (Comparator);
Clinical profile, outcomes and predictors of mortality in neonates operated for gastrointestinal anomalies in a tertiary neonatal care unit- An observational study	Jerry, A.L.; Amboiram, P.; Balakrishnan, U.; Chandrasekaran, A.; Agarwal, P.; Devi, U.	Details on whether transfer required? (Comparator);
