# Supplementary material for: Association between birth location and short-term outcomes for babies with gastroschisis, congenital diaphragmatic hernia and oesophageal fistula: a systematic review
Source: BMJ Paediatr Open. 2023 Jul 19;7(1):e002007. doi: 10.1136/bmjpo-2023-002007 (PMC10357737; doi:10.1136/bmjpo-2023-002007)
Supplement: Supplementary data [file bmjpo-2023-002007supp002.pdf]

**CONGENITAL DIAPHRAGMATIC HERNIA**											
**Author/Year**	**Setting**	**Population**	**Study Sample**	**Exclusions**	**Comparison**	**Outcomes**	**Analysis**	**Results – absolute numbers**	**Results – analysis presented**	**Adjustments for confounders**	**Author's conclusion on the effect of birth location on outcomes ?****
Al-Shanafey et al., 2002 (17)	Halifax, Canada	Infants born with CDH born in or referred to regional unit between 1973-1999	81 infants total : 41 transferred to surgical centre (outborn)	Diagnosis of lethal cardiac malformation Prenatal deaths and deaths immediately upon delivery	Born in specialist surgical centres vs born outside and referred	Mortality	Descriptive Chi-square test	No transfer: 9/40 died Transfer: 7/41 died	Non-significant difference between groups P=0.54	Nil	No difference
Algert et al., 2008 (12)	New South Wales, Australia	Infants born alive with CDH managed in a single regional centre between 2001-2003	57 infants total: 22 transferred to surgical centre (outborn)	Diagnosis made >28days after birth Antenatally diagnosable and lethal uncorrectable defects such as anencephaly, renal agenesis and trisomy 13 or 18	'Co-located hospital' – not requiring neonatal emergency transport vs with 'Other hospital' – requiring transport	1-year Mortality	Test of proportions	No transfer: 19/35 died Transfer: 4/22 died	Significant difference between groups P<0.01	Nil	Favour birth in surgical centre/ regionalisation of services
Aly et al., 2010 (21)	USA: Kids' Inpatient Database	All infants with CDH and respiratory distress requiring mechanical ventilation admitted to selected hospitals during 1997-2004 ς	2140 infants total: 1020 transferred to surgical centre (outborn)	Brain anomalies, abdominal wall defects, other congenital anomalies	Group 1 did not undergo transport vs group 2 transported from the birth hospital to the surgical facility	Mortality after surgery	Logistic regression modelling	No transfer: 143/1120 died Transfer: 165/1020 died	aOR: 1.46 (1.02-2.05) P=0.02	Gender, race, birth weight category, age at operation, comorbidities (pneumothorax, pulmonary hypertension, sepsis, necrotizing enterocolitis, birth asphyxia)	Favour birth in surgical centre/ regionalisation of services
Boloker et al., 2002 (22)	New York, USA	Infants born with CDH and admitted to a single surgical centre during 1992-2000 ς	120 infants total: 53 transferred to surgical centre (outborn)	Other congenital anomalies	Inborn vs outborn transfers	Mortality	Descriptive	No transfer: 23/67 died Transfer: 6/53 died		Nil	No difference
Carmichael et al., 2020 (23)	California, USA	Infants born with CDH during 2006-2011 in California ς	577 infants total: 357 transferred to surgical centre (outborn)	Infants with time of diagnosis unknown and infants that had fetal surgery	Transfer from birth hospital for repair vs no transfer from birth hospital	Mortality to infancy	Descriptive Hazard Ratio	No transfer: 103/220 died Transfer: 77/357 died	Unadjusted: HR: 0.4(0.3-0.5) Adjusted: aHR: 0.3 (0.2-0.5)	Demographics, Birthweight Gestational age Other birth defects	Favour birth in surgical centre/ regionalisation of services

Gallot et al., 2007 (18)	Central-Eastern France	Infants born with CDH during 1986-2003	387 infants total: 211 transferred (outborn)	Nil	Delivery in tertiary hospital vs peripheral hospital and transfer	Mortality	Descriptive Chi-square test	No transfer: 78/176 died Transfer: 74/211 died	Group difference: P=0.06	Nil	Favour birth in surgical centre/ regionalisation of services
Nagata et al., 2013 (20)	Japan	Infants born with CDH during 2006-2010 ς	614 infants total: 165 transferred to surgical centre (outborn)	Diagnosis made >28days after birth	Inborn vs outborn	Mortality	Descriptive Chi-square test	No transfer: 129/449 died Transfer: 22/165 died	Group difference: P<0.001	Nil	N/A
Nasr and Langer, 2011 (15)	Canada	Infants born with CDH during 2005-2008 ς	140 infants total: 65 transferred (outborn)	Postnatal diagnosis	Inborn: born in the same building as NICU vs outborn requiring an ambulance transfer	Mortality	Descriptive Multivariate analysis	No transfer: 17/75 died Transfer: 21/65	Group difference: P=0.30 aOR 2.8, P=0.04	Adjustment for possible case-mix – unspecified variables	Favour birth in surgical centre/ regionalisation of services
Putnam et al., 2016 (29)	Congenital Diaphragmatic Hernia Study Group: Australia, Canada, Chile, Germany, Italy, Japan, Malaysia, Poland, Russia, Scotland, Sweden, The Netherlands, USA	Infants born with CDH during 2007-2014 ς	3665 infants: number of transferred not available	Nil	Inborn vs outborn	Pulmonary morbidity<br><br>Neurologic morbidity<br><br>Gastrointestinal morbidity	Descriptive Multivariate analysis	327/660 transferred and had pulmonary morbidity<br><br>228/446 transferred and had neurologic morbidity<br><br>787/1348 transferred and had gastrointestinal morbidity	OR 1.25 (0.86-1.84), P=0.247<br><br>OR 1.04 (0.37-1.47), P=0.848<br><br>OR 1.64 (1.11-2.42), P=0.013	Patient characteristics, including defect size	No difference
Sola et al., 2010 (24)	USA – Kids' Inpatient Database	Infants with CDH during 1997-2006 ς	2774 infants total: 891 transferred (outborn)	Admission >8 days of age	Hospital transfer vs birth	Mortality	Descriptive Chi-square test	No transfer: 333/1680 died Transfer: 212/891 died	Group difference: P=0.003	Nil	No difference
Stopenski et al., 2022 (30)	Congenital Diaphragmatic Hernia Study Group: Argentina, Canada, Chile, Egypt, England, Germany, Italy,	Infants with CDH during 2007-2019 with prenatal diagnosis ς	4195 infants total: 1108 transferred (outborn)	Postnatal diagnosis	Inborn-delivered and treated at CDHSG hospitals. Outborn - delivered in a separate	Mortality	Descriptive Logistic regression	No transfer: 2082/3087 died Transfer: 733/1108 died	Group difference: P=0.44<br><br>OR: 1.07 (0.015-1.25) P=0.391	Demographics Clinical severity	No difference

	Japan, Malaysia, Netherlands, Poland, Qatar, Russia, Scotland, Spain, Sweden, USA				institution and required transfer to a definitive care center.						

Supplementary Table 1a: Summary of studies on CDH included.

*not all studies provided statistical analysis i.e., ORs, some studies only described the number of events

**not all authors provided conclusions on favoured birth location

RR- Risk Ration, aRR- adjusted Risk Ratio, OR – Odds Ratio ,aOR – adjusted Odds Ratio, P- p-value, HR hazard ratio, aHR – adjusted Hazard Ratio

† Length of stay given as median (IQR)

ς Potentially overlapping cohort: multicentre or population-based studies may include duplicates infants

**GASTROSCHISIS**											
**Author/Year**	**Setting**	**Population**	**Study Sample**	**Exclusions**	**Comparison**	**Outcomes**	**Analysis**	**Results – absolute numbers**	**Results – analysis presented**	**Adjustments for confounders**	**Author's conclusion on the effect of birth location on outcomes ?****
Algert et al., 2008 (12)	New South Wales, Australia	Infants born with gastroschisis during 2001-2003	62 infants: 11 transferred to a surgical centre	Diagnosis made >28days after birth Infants with antenatally diagnosable and lethal uncorrectable defects such as anencephaly, renal agenesis and trisomy 13 or 18	Co-located hospital – not requiring neonatal emergency transport compared with Other hospital – requiring transport	1-year Mortality	Test of Proportion	Transfer: 0/11 died<br>No transfer: 7/51 died	Non-significant difference	Nil	No difference
Dalton et al., 2017 (25)	Kansas, USA	Infants born with gastroschisis during 2010-2015 ς	79 infants total: 18 transferred to surgical centre (outborn)	Complicated gastroschisis diagnosis	Inborn vs outborn	Length of stay†<br><br>Days to first feed	Descriptive Fisher's exact test	Transfer: 40+/- 26 days<br>No transfer: 30+/- 15 days<br><br>Transfer: 15+/-8.4 days<br>No transfer: 13+/-8.5 days	Group difference: P= 0.03<br><br>Group difference: P=0.17	Nil	Favour birth in surgical centre/ regionalisation of services

						Days to full feed<br><br>Total Parenteral Nutrition days		Transfer: 36+/- 23 days<br>No transfer: 16 +/- 54 days<br><br>Transfer: 32+/- 23 days<br>No transfer: 23+/- 12 days	Group difference: P=0.07<br><br>Group difference: P= 0.03		
Hong et al., 2019 (26)	USA: Vermont Oxford Network	Infants >1500g born with gastroschisis during 2009-2015 ς	4663 infants total: 1134 transferred (outborn)	Infants transferred after repair	Inborn – birth in surgical centre vs outborn – delivery outside of centre	Mortality<br><br>Length of stay†	Multivariate logistic regression	Transfer: 23/1134 died<br>No transfer: 67/3529 died<br><br>Transfer: 38 (27,64) days<br>No transfer: 35 (26,55) days	aOR: 1.02 (0.60-1.72) P>0.05<br><br>Poisson regression: 0.10 (0.04,0.16) P<0.05	Clustering within hospitals	No difference
Kandasamy et al., 2009 (13)	North Queensland, Australia	Infants born with gastroschisis during 1988-2007 ς	50 infants total: 10 transferred (outborn)	Nil	Inborn vs outborn	Mortality<br><br>Length of stay	Chi-square test	No numerical data available – No difference between groups found			No difference
Kitchanan et al., 2000 (14)	North Queensland, Australia	Infants born with gastroschisis during 1988-1997ς	21 infants (inborn): 4 transferred (outborn)	Nil	Inborn – delivered in regional tertiary unit vs outborn – delivered in peripheral hospitals	Days to first feed<br><br>Days to full enteral feeding<br><br>Total Parenteral Nutrition days	Descriptive Non-parametric test	Transfer: 25 days<br>No transfer: 9 days<br><br>Transfer: 19 days<br>No transfer: 16 days<br><br>Transfer: 42 days<br>No transfer: 14 days	Group difference: P=0.012<br><br>Group difference: P= 0.02<br><br>Group difference: P=0.012		No difference
Nasr and Langer, 2012 (16)	Canada	Infants born with gastroschisis during 2005-2008	395 infants: 158 transferred	Postnatal diagnosis	Inborn: born in the same building as NICU vs outborn requiring an	Mortality<br><br>Length of	Descriptive Logistic regression	Transfer: 4/158 died<br>No transfer: 11/237 died<br><br>Transfer: 49 (27,62)	Group difference: P=0.10<br><br>Group difference:	Possible case-mix – not specified	No difference

					ambulance transfer	stay†<br><br>Days to first feed<br><br>Total Parental Nutrition days<br><br>Complications		days<br>No transfer: 48 (21,54) days<br><br>Transfer: 12 (6,14) days<br>No transfer: 12 (7,15) days<br><br>Transfer: 42 (22,40) days<br>No transfer: 36 (17,40) days<br><br>Transfer: 4/158<br>No transfer: 11/237	P=0.80<br><br>Group difference: P=0.85<br><br>Group difference: P= 0.19<br><br>Group difference: P=0.10<br>OR 1.60 (1.09-2.7), P=0.05		
Savoie et al., 2014 (27)	Paediatric Surgery Research Collaborative centres, USA: Memphis, Houston, Nashville, Little Rock, Indianapolis, Kansas	Infants with gastroschisis during 2008-2013	524 infants total: 239 transferred outborn	Nil	Inborn-delivery in hospital where surgical repair occurred vs outborn-required transfer to surgical centre for repair	30-day Mortality<br><br>Length of stay†<br><br>Days to first enteral feed<br><br>Days to full enteral feeding<br><br>Total Parenteral Nutrition days	Descriptive<br>T-test<br>Chi-square<br>Multivariable analyses	Transfer: 4/239 died<br>No transfer: 4/283 died<br><br>Transfer: 42 (28,76) days<br>No transfer: 34 (24,63) days<br><br>Transfer: 18 (13,26) days<br>No transfer: 16 (11,22) days<br><br>Transfer: 31 (23,57) days<br>No transfer: 27 (20,43) days<br><br>Transfer: 29 (20,54)	Group difference: P=0.81<br><br>Group difference: P=0.002<br><br>Group difference: P=0.004<br><br>Group difference: P=0.0008<br><br>Group difference: P=0.06	Possible confounders – not specified	Favour birth in surgical centre/ regionalisation of services

								days<br>No transfer:<br>27 (17,445)<br>days			

Supplementary Table 1b: Summary of studies on gastroschisis included.

*not all studies provided statistical analysis i.e., ORs, some studies only described the number of events

**not all authors provided conclusions on favoured birth location

RR- Risk Ration, aRR- adjusted Risk Ratio, OR – Odds Ratio ,aOR – adjusted Odds Ratio, P- p-value, HR hazard ratio, aHR – adjusted Hazard Ratio

† Length of stay given as median (IQR)

ς Potentially overlapping cohort: multicentre or population-based studies may include duplicates infants

**OESOPHAGEAL ATRESIA / TRACHEOESOPHAGEAL FISTULA**											
**Author/Year**	**Setting**	**Population**	**Study Sample**	**Exclusions**	**Compairson**	**Outcomes**	**Analysis**	**Results – absolute numbers**	**Results – analysis presented**	**Adjustments for confounders**	**Author's conclusion on the effect of birth location on outcomes ?****
Algert et al., 2008 (12)	New South Wales, Australia	Infants with oesophageal atresia during 2001-2003	42 infants: 25 transferred	Diagnosis made >28days after birth<br>Antenatally diagnosable and lethal uncorrectable defects such as anencephaly, renal agenesis and trisomy 13 or 18	Co-located hospital – not requiring neonatal emergency transport compared with 'Other hospital' – requiring transport	1-year Mortality	Test of Proportion	Transfer:<br>1/25 died<br>No transfer:<br>3/17 died	Non-significant difference between groups	Nil	No differences
Schlee et al., 2022 (19)	Frankfurt, Germany	Infants with oesophageal atresia during 2009-2013	57 infants total: 35 transferred (outborn)	Nil	Inborn-born in institution vs outborn – referred postnatally and transported for surgery	1-year mortality<br><br>Length of stay†<br><br>Days to full enteral feeding	Descriptive<br>Mann-Whitney U test<br>Fisher's exact test	Transfer:<br>0/35 died<br>No transfer:<br>2/17 died<br><br>Transfer:<br>17 (13,36) days<br>No transfer:<br>92 (18,158) days<br><br>Transfer:<br>8 (6,11) days<br>No transfer:<br>12 (7,18) days	Group Difference:<br>P= 0.103<br><br>Group difference:<br>P= 0.009<br><br>Group difference:<br>P=0.137	Nil	No difference

Wang et al., 2014 (28)	USA: Kids' Inpatient Database	Infants with oesophageal atresia/trachoesophageal fistula during 1997-2009	4168 infants total: 1169 transferred (outborn)	Nil	Divided by admission source: Birth vs Hospital transfer	Mortality	Descriptive Fisher's exact test	Transfer: 99/1169 died<br>No transfer: 186/1850 died	Group difference: P=0.399	Nil	No difference

Supplementary Table 1c: Summary of studies on TOF/OA included.

*not all studies provided statistical analysis i.e., ORs, some studies only described the number of events

**not all authors provided conclusions on favoured birth location

RR- Risk Ration, aRR- adjusted Risk Ratio, OR – Odds Ratio ,aOR – adjusted Odds Ratio, P- p-value, HR hazard ratio, aHR – adjusted Hazard Ratio

† Length of stay given as median (IQR)

ς Potentially overlapping cohort: multicentre or population-based studies may include duplicates infants
