# Supplementary material for: Association between birth location and short-term outcomes for babies with gastroschisis, congenital diaphragmatic hernia and oesophageal fistula: a systematic review
Source: BMJ Paediatr Open. 2023 Jul 19;7(1):e002007. doi: 10.1136/bmjpo-2023-002007 (PMC10357737; doi:10.1136/bmjpo-2023-002007)
Supplement: Author's manuscript [file bmjpo-2023-002007.draft_revisions.pdf]

BMJ Paediatrics Open

BMJ Paediatrics Open is committed to open peer review. As part of this commitment we make the peer review history of every article we publish publicly available.

When an article is published we post the peer reviewers' comments and the authors' responses online. We also post the versions of the paper that were used during peer review. These are the versions that the peer review comments apply to.

The versions of the paper that follow are the versions that were submitted during the peer review process. They are not the versions of record or the final published versions. They should not be cited or distributed as the published version of this manuscript.

BMJ Paediatrics Open is an open access journal and the full, final, typeset and author-corrected version of record of the manuscript is available on our site with no access controls, subscription charges or pay-per-view fees (http://bmjpaedsopen.bmj.com).

If you have any questions on BMJ Paediatrics Open's open peer review process please email info.bmjpo@bmj.com

BMJ Paediatrics Open

Association between birth location and short-term outcomes for babies with gastroschisis, congenital diaphragmatic hernia and oesophageal fistula: a systematic review

Journal:	*BMJ Paediatrics Open*
Manuscript ID	bmjpo-2023-002007
Article Type:	Original research
Date Submitted by the Author:	03-Apr-2023
Complete List of Authors:	Nezafat Maldonado, Behrouz; Imperial College London, Neonatal Medicine Chelsea and Westminster Hospital campus<br>Singhal, Graciaa; Hull York Medical School<br>Chow, LiYan; Chelsea and Westminster Hospital NHS Foundation Trust, Neonatal Medicine Department<br>Hargreaves, Dougal; Imperial College London School of Public Health<br>Gale, Chris; Imperial College London, Section of Neonatal Medicine<br>Battersby, Cheryl; Imperial College London, Neonatal Medicine Chelsea and Westminster Hospital campus
Keywords:	Neonatology

SCHOLARONE™
Manuscripts

I, the Submitting Author has the right to grant and does grant on behalf of all authors of the Work (as defined in the below author licence), an exclusive licence and/or a non-exclusive licence for contributions from authors who are: i) UK Crown employees; ii) where BMJ has agreed a CC-BY licence shall apply, and/or iii) in accordance with the terms applicable for US Federal Government officers or employees acting as part of their official duties; on a worldwide, perpetual, irrevocable, royalty-free basis to BMJ Publishing Group Ltd ("BMJ") its licensees and where the relevant Journal is co-owned by BMJ to the co-owners of the Journal, to publish the Work in this journal and any other BMJ products and to exploit all rights, as set out in our licence.

The Submitting Author accepts and understands that any supply made under these terms is made by BMJ to the Submitting Author unless you are acting as an employee on behalf of your employer or a postgraduate student of an affiliated institution which is paying any applicable article publishing charge ("APC") for Open Access articles. Where the Submitting Author wishes to make the Work available on an Open Access basis (and intends to pay the relevant APC), the terms of reuse of such Open Access shall be governed by a Creative Commons licence – details of these licences and which Creative Commons licence will apply to this Work are set out in our licence referred to above.

Other than as permitted in any relevant BMJ Author's Self Archiving Policies, I confirm this Work has not been accepted for publication elsewhere, is not being considered for publication elsewhere and does not duplicate material already published. I confirm all authors consent to publication of this Work and authorise the granting of this licence.

Confidential: For Review Only

Association between birth location and short-term outcomes for babies with gastroschisis, congenital diaphragmatic hernia and oesophageal fistula: a systematic review

B Nezafat Maldonado[1], G Singhal[2], L Chow[3], D Hargreaves[4], C Gale[1], C Battersby[1]

1. Neonatal Medicine, School of Public Health, Faculty of Medicine, Imperial College London, Chelsea & Westminster Hospital campus, London, United Kingdom
2. Hull York Medical School,
3. Neonatal Medicine Department, Chelsea and Westminster NHS Foundation Trust
4. Mohn Centre for Children's Health and Wellbeing, Imperial College London

Corresponding author:
Dr Behrouz Nezafat Maldonado
Neonatal Medicine
School of Public Health
Imperial College London
Chelsea and Westminster Hospital Campus
S W10 9NH
bnezafat@imperial.ac.uk

Dr Behrouz Nezafat Maldonado
Neonatal Medicine, School of Public Health, Faculty of Medicine, Imperial College London, Chelsea & Westminster Hospital campus, London, United Kingdom

Ms Graciaa Singhal
Hull York Medical Schol

Dr Dougal Hargreaves
Mohn Centre for Children's Health and Wellbeing, Imperial College London

Professor Chris Gale
Neonatal Medicine, School of Public Health, Faculty of Medicine, Imperial College London, Chelsea & Westminster Hospital campus, London, United Kingdom

Dr Cheryl Battersby
Neonatal Medicine, School of Public Health, Faculty of Medicine, Imperial College London, Chelsea & Westminster Hospital campus, London, United Kingdom

Key words: Neonatology
Word count: 2601 excluding title page, abstract, references, figures and tables

Confidential: For Review Only

ABSTRACT

Background

Neonatal care is commonly regionalised, meaning specialist services are only available at certain units. Consequently, infants with surgical conditions needing specialist care that are born in non-surgical centres require postnatal transfer. Best practice models advocate for co-located maternity and surgical services as the place of birth for infants with antenatally diagnosed congenital conditions to avoid postnatal transfers. We conducted a systematic review to explore the association between location of birth and short-term outcomes of babies with gastroschisis, congenital diaphragmatic hernia (CDH) and oesophageal atresia with or without tracheoesophageal fistula (TOF/OA).

Methods

We searched MEDLINE, CINAHL, Web of Science and SCOPUS databases for studies from high income countries comparing outcomes for infants with gastroschisis, CDH or TOF/OA based on their place of delivery. Outcomes of interest included mortality, length of stay, age at first feed, comorbidities, and duration of parenteral nutrition. We assessed study quality using the Newcastle-Ottawa Scale. We present a narrative synthesis of our findings.

Results

Nineteen cohort studies compared outcomes of babies with one of gastroschisis, CDH or TOF/OA. Heterogeneity across the studies precluded meta-analysis. Eight studies carried out case-mix adjustments. Overall, we found conflicting evidence. There is limited evidence to suggest that birth in a maternity unit with a co-located surgical centre was associated with a reduction in mortality for CDH and decreased length of stay for gastroschisis.

Conclusions

There is little evidence to suggest that delivery in co-located maternity-surgical services may be associated with shortened length of stay and reduced mortality. Our findings are limited by significant heterogeneity, potential for bias and paucity of strong evidence. This supports the need for further research to investigate the impact of birth location on outcomes for babies with congenital surgical conditions and inform future design of neonatal care systems.

Review registration PROSPERO Registration number: CRD42022329090

What is already known on this topic

Infants with congenital surgical anomalies require specialist surgical care and neonatal care. This specialist care may not be available across all units. As a result, some infants are delivered in a non-surgical centre and are then postnatally transferred to a surgical centre. International consensus recommends delivery in co-located maternity and surgical units for babies with antenatally diagnosed surgical conditions.

What this study adds

We systematically identified international data exploring the impact of birth in a non-surgical centre on neonatal outcomes for infants with CDH, gastroschisis or TOF/OA. There is limited evidence to suggest that birth in a non-surgical centre may be associated with increased mortality, prolonged length of stay and delayed enteral feeding.

How this study might affect research, practice or policy

This study highlights key gaps in the evidence; studies able to account for case-mix and clinical severity and reporting on core outcomes across different birth locations are needed to inform future development of neonatal care systems.

Confidential: For Review Only

INTRODUCTION:

Infants with congenital surgical conditions require specialist surgical input soon after birth. It is important to ensure that specialist services are available to adequately care after these babies. Neonatal care may be delivered through centralised or decentralised models. This means that different levels of care may be available at birth across health care settings.

Antenatal care aims to identify congenital anomalies, for optimal delivery planning with access to specialist care. Guidelines recommend delivery in a centre with co-located neonatal intensive care and neonatal surgical services for infants with suspected surgical anomalies (1–3). However, socioeconomic, environmental or health factors, or due to the nature of the defect anomalies are not identified antenatally, and infants are born in a unit without surgical services. Postnatal transfer can hinder parental access and bonding with their baby, as complex infants may be cared for in specialist centres far from the family residence. Clinical pathways should enable provision of intensive care to all infants where this is required, while minimising postnatal transfer (3).

To improve neonatal surgical care, we must understand how birthplace impacts outcomes for congenital anomalies. Some anomalies, such as gastroschisis, are often diagnosed antenatally, while others, are more like tracheoesophageal fistula or oesophageal atresia (TOF/OA) are not. Other defects such as congenital diaphragmatic hernia (CDH) may be antenatally diagnosed and require intensive care from birth, meaning delivery in the right place can be key. As a result, planning on place of delivery varies based on the condition. These three conditions, that may be detected or present in different ways, serve as exemplar congenital anomalies to explore the impact of birth location on short-term outcomes.

This review aims to understand the impact of delivery location on short term neonatal outcomes for infants born with congenital diaphragmatic hernia (CDH), gastroschisis and tracheoesophageal fistula or oesophageal atresia (TOF/OA). We aim to map the latest evidence to inform future research and influence neonatal service design.

METHOD:

Search methods for identification of studies

This systematic review was prospectively registered in PROSPERO (Registration number: CRD42022329090) and conducted in accordance with PRISMA (4,5). We searched the following databases: MEDLINE, CINAHL, Web of Science and SCOPUS. Supplementary File 1 includes example search strategy.

Selection criteria

Studies were eligible for inclusion if they:

I. were published between 1stJanuary2000 and 15thJanuary2023 in any language,
II. were conducted in a high-income country as defined by the World Bank (6)
III. included a study population of infants diagnosed with one of CDH, gastroschisis and TOF/OA
IV. provided data on birth location across at least two groups and,
V. reported at least one outcome of interest

Studies were excluded if they did not report delivery location, did not provide quantitative data or did not allow for comparisons. We also searched the bibliography of included studies, grey literature, and contacted experts for relevant papers.

Outcomes of interest

Outcomes of interest were mortality, length of stay, time to full enteral feeds, time to first feed, sepsis, presence of morbidity, duration of parenteral nutrition, presence of liver disease, growth and breastfeeding rates(as a proxy measure for maternal-separation). Outcomes were decided upon reviewing the literature, including core outcome set on gastroschisis and core outcome set in neonatology (7,8).

Selection of studies

Three reviewers (BNM, LC, GS) screened title and abstracts using CADIMA software for records management, deduplicate, screening and data extraction (9). Conflicts were resolved by team discussions. Data extraction

Confidential: For Review Only

was carried out using a predetermined template to include baseline characteristics and outcome data. Two reviewers (BNM and GS) conducted data extraction independently with conflicts resolved through discussions. Only data related to CDH, gastroschisis, or TOF/OA were extracted from studies involving infants with other surgical conditions.

Risk of bias and quality of evidence assessment

The Newcastle Ottawa Scale (NOS) assessed quality and risk of bias of included studies (10), using nine points across three domains: (I) participants selection; (II) comparability and (III) detection of exposure/outcome. Depending on the scoring, studies were rated 'good', 'fair', or 'poor' quality using standard guidance (Table 2). The NOS assessment was independently done by two reviewers (BNM and GS) and discrepancies resolved by discussion. Studies that included more than one condition of interest but used the same methodology were assessed once. Studies that include the same population or potentially overlapping populations were independently assessed.

Data extraction and analysis

Data extraction followed a predefined collection form. We present findings grouped by condition. For each outcome we present, when available, the absolute rates as well as unadjusted and adjusted results in cases and control, and results of statistical significance tests. We use vote-counting based on the direction of effect methodology, comparing the number of positive studies – that showed benefit – to the number of negative studies – showing harm. We report direction of effect by outcome and study quality, and we also present the overall effect of birth location (11). The author's conclusion was also considered in the overall effect.

Patient and public involvement statement

No patients or members of the public were involved in this systematic review. As this was a review of available literature, no ethics approval was necessary.

RESULTS:

Description of studies

Of 1398 records retrieved, 909 remained after deduplication and were screened on title and abstract. 813 were excluded on title and abstract and ninety-five full texts were retrieved and assessed for eligibility. Nineteen studies met our inclusion criteria and were included in the review. Reasons for exclusion included the study setting, the lack of a control and exposure group or no comparison between groups provided in the results. Grey literature and bibliography searches did not yield any further references. Figure 1 shows the PRISMA flow diagram for the records found and screened.

Study characteristics

Studies were either conducted in a single country e.g., Australia(12–14), Canada (15–17), France (18), Germany (19), Japan (20), USA (21–28), or at an international level across multiple countries (29,30). One studied included data on all three congenital malformations of interest for the review (12), whilst the rest focused on only one of them. Study size ranged from 50 to 4663 babies, and we expect overlap in the populations included across the studies in view of the time-period and settings included. Case-mix adjustments were carried out in eight studies (12,13,15,16,22,27,29,31). The main characteristics of included studies are summarised in Table 1.

Mortality was most reported; however, the definition of mortality was inconsistent across studies. Other outcomes reported included length of hospital stay, postnatal age at first feed, postnatal age at full enteral feeding, presence of comorbidities and duration of total parenteral nutrition.

Quality assessment

We assessed study quality using the NOS (10). A composite score depending on the score across the three domains was assigned to each study, as described previously. Study quality assessment is summarised on Table 2. All studies performed well in the selection and outcome domains of the tool, however performance in the comparability domain was variable. Ten studies were rated as 'good' quality, and nine as 'poor' quality.

Protocol modifications

https://mc.manuscriptcentral.com/bmjpo

Confidential: For Review Only

High degree of heterogeneity precluded a meta-analysis. Instead, we present a narrative synthesis with vote-counting on the direction of effect (11).

Vote counting for measure of effect

We present the direction of effect for each study for the outcomes reported. In addition, we report the overall direction of effect of birth in surgical centre for CDH, gastroschisis and TOF/OA. Figure 2 displays vote-counting on overall direction of effect. Table 3 summarises direction of effect per outcome and the quality rating per study.

Confidential: For Review Only

Author/Year	Setting	Population	Study Sample	Exclusions	Outcomes	Analysis	Results – absolute numbers	Results – analysis presented	Adjustments for confounders	Author's conclusion on the effect of birth location on outcomes ?**
**CONGENITAL DIAPHRAGMATIC HERNIA**										
Al-Shanafey et al., 2002 (17)	Halifax, Canada	Infants born with CDH born in or referred to regional unit between 1973-1999	81 infants: 41 transferred to surgical centre	Diagnosis of lethal cardiac malformation Prenatal deaths and deaths immediately upon delivery	Mortality	Descriptive Chi-square test	No transfer: 9/40 died Transfer: 7/41 died	Non-significant difference between groups P=0.54	Nil	No difference
Algert et al., 2008 (12)	New South Wales, Australia	Infants born alive with CDH managed in a single regional centre between 2001-2003	57 infants: 22 transferred to surgical centre	Diagnosis made >28days after birth Antenatally diagnosable and lethal uncorrectable defects such as anencephaly, renal agenesis and trisomy 13 or 18	1-year Mortality	Test of proportions	No transfer: 19/35 died Transfer: 4/22 died	Significant difference between groups P<0.01	Nil	Favour birth in surgical centre/ regionalisation of services
Aly et al., 2010 (21)	USA: Kids' Inpatient Database	All infants with CDH and respiratory distress requiring mechanical ventilation admitted to selected hospitals during 1997-2004 ς	2140 infants: 1020 transferred to surgical centre	Brain anomalies, abdominal wall defects, other congenital anomalies	Mortality after surgery	Logistic regression modelling	No transfer: 143/1120 died Transfer: 165/1020 died	aOR: 1.46 (1.02-2.05) P=0.02	Gender, race, birth weight category, age at operation, comorbidities (pneumothorax, pulmonary hypertension, sepsis, necrotizing enterocolitis, birth asphyxia)	Favour birth in surgical centre/ regionalisation of services
Boloker et al., 2002 (22)	New York, USA	Infants born with CDH and admitted to a single surgical centre during 1992-2000 ς	120 infants: 53 transferred to surgical centre	Other congenital anomalies	Mortality	Descriptive	No transfer: 23/67 died Transfer: 6/53 died		Nil	No difference
Carmichael et al., 2020 (23)	California, USA	Infants born with CDH during 2006-2011 in California ς	577 infants: 357 transferred to surgical centre	Infants with time of diagnosis unknown	Mortality to infancy	Descriptive Hazard Ratio	No transfer: 103/220 died Transfer: 77/357 died	Unadjusted: HR: 0.4(0.3-0.5) Adjusted: aHR: 0.3 (0.2-0.5)	Demographics, Birthweight Gestational age Other birth defects	Favour birth in surgical centre/ regionalisation of services
Gallot et al., 2007 (18)	Central-Eastern France	Infants born with CDH during 1986-2003	387 infants: 211 transferred	Nil	Mortality	Descriptive Chi-square test	No transfer: 78/176 died Transfer: 74/211 died	Group difference: P=0.06	Nil	Favour birth in surgical centre/ regionalisation of services
Nagata et al., 2013 (20)	Japan	Infants born with CDH during 2006-2010 ς	614 infants: 165 transferred to surgical centre	Diagnosis made >28days after birth	Mortality	Descriptive Chi-square test	No transfer: 129/449 died Transfer: 22/165 died	Group difference: P<0.001	Nil	N/A
Nasr and Langer, 2011 (15)	Canada	Infants born with CDH during 2005-2008 ς	140 infants: 65 transferred	Postnatal diagnosis	Mortality	Descriptive Multivariate analysis	No transfer: 17/75 died	Group difference:	Adjustment for possible case-mix –	Favour birth in surgical centre/

Confidential: For Review Only

							Transfer: 21/65	P=0.30<br>aOR 2.8, P=0.04	unspecified variables	regionalisation of services
Putnam et al., 2016 (29)	Congenital Diaphragmatic Hernia Study Group: Australia, Canada, Chile, Germany, Italy, Japan, Malaysia, Poland, Russia, Scotland, Sweden, The Netherlands, USA	Infants born with CDH during 2007-2014 ς	3665 infants: number of transferred not available	Nil	Pulmonary morbidity<br><br>Neurologic morbidity<br><br>Gastrointestinal morbidity	Descriptive<br>Multivariate analysis	327/660 transferred and had pulmonary morbidity<br><br>228/446 transferred and had neurologic morbidity<br><br>787/1348 transferred and had gastrointestinal morbidity	OR 1.25 (0.86-1.84), P=0.247<br><br>OR 1.04 (0.37-1.47), P=0.848<br><br>OR 1.64 (1.11-2.42), P=0.013	Patient characteristics, including defect size	No difference
Sola et al., 2010 (24)	USA – Kids' Inpatient Database	Infants with CDH during 1997-2006 ς	2774 infants: 891 transferred	Admission >8 days of age	Mortality	Descriptive<br>Chi-square test	No transfer: 333/1680 died<br>Transfer: 212/891 died	Group difference: P=0.003	Nil	No difference
Stopenski et al., 2022 (30)	Congenital Diaphragmatic Hernia Study Group: Argentina, Canada, Chile, Egypt, England, Germany, Italy, Japan, Malaysia, Netherlands, Poland, Qatar, Russia, Scotland, Spain, Sweden, USA	Infants with CDH during 2007-2019 with prenatal diagnosis ς	4195 infants: 1108 transferred	Postnatal diagnosis	Mortality	Descriptive<br>Logistic regression	No transfer: 2082/3087 died<br>Transfer: 733/1108 died	Group difference: P=0.44<br><br>OR: 1.07 (0.015-1.25) P=0.391	Demographics<br>Clinical severity	No difference
**GASTROSCHISIS**										
Algert et al., 2008 (12)	New South Wales, Australia	Infants born with gastroschisis during 2001-2003	62 infants: 11 transferred to a surgical centre	Diagnosis made >28days after birth<br>Antenatally diagnosable and lethal uncorrectable defects such as anencephaly, renal agenesis and trisomy 13 or 18	1-year Mortality	Test of Proportion	Transfer: 0/11 died<br>No transfer: 7/51 died	Non-significant difference	Nil	No difference
Dalton et al., 2017 (25)	Kansas, USA	Infants born with gastroschisis during 2010-2015 ς	79 infants: 18 transferred to surgical centre	Complicated gastroschisis diagnosis	Length of stay†	Descriptive<br>Fisher's exact test	Transfer: 40+/- 26 days	Group difference:	Nil	Favour birth in surgical centre/

Confidential: For Review Only

					Days to first feed<br><br>Days to full feed<br><br>Total Parenteral Nutrition days		No transfer: 30+/- 15 days<br><br>Transfer: 15+/-8.4 days<br>No transfer: 13+/-8.5 days<br><br>Transfer: 36+/- 23 days<br>No transfer: 16 +/- 54 days<br><br>Transfer: 32+/- 23 days<br>No transfer: 23+/- 12 days	P= 0.03<br><br>Group difference: P=0.17<br><br>Group difference: P=0.07<br><br>Group difference: P= 0.03		regionalisation of services
Hong et al., 2019 (26)	USA: Vermont Oxford Network	Infants >1500g born with gastroschisis during 2009-2015 ς	4663 infants: 1134 transferred	Infants transferred after repair	Mortality<br><br>Length of stay†	Multivariate logistic regression	Transfer: 23/1134 died<br>No transfer: 67/3529 died<br><br>Transfer: 38 (27,64) days<br>No transfer: 35 (26,55) days	aOR: 1.02 (0.60-1.72) P>0.05<br><br>Poisson regression: 0.10 (0.04,0.16) P<0.05	Clustering within hospitals	No difference
Kandasamy et al., 2009 (13)	North Queensland, Australia	Infants born with gastroschisis during 1988-2007 ς	50 infants: 10 transferred	Nil	Mortality<br><br>Length of stay	Chi-square test	No numerical data available – No difference between groups found			No difference
Kitchanan et al., 2000 (14)	North Queensland, Australia	Infants born with gastroschisis during 1988-1997ς	21 infants: 4 transferred	Nil	Days to first feed<br><br>Days to full enteral feeding<br><br>Total Parenteral Nutrition days	Descriptive Non-parametric test	Transfer: 25 days<br>No transfer: 9 days<br><br>Transfer: 19 days<br>No transfer: 16 days<br><br>Transfer: 42 days<br>No transfer: 14 days	Group difference: P=0.012<br><br>Group difference: P= 0.02<br><br>Group difference: P=0.012		No difference

Confidential: For Review Only

Nasr and Langer, 2012 (16)	Canada	Infants born with gastroschisis during 2005-2008	395 infants: 158 transferred	Postnatal diagnosis	Mortality<br><br>Length of stay†<br><br>Days to first feed<br><br>Total Parental Nutrition days<br><br>Complications	Descriptive<br>Logistic regression	Transfer: 4/158 died<br>No transfer: 11/237 died<br><br>Transfer: 49 (27,62) days<br>No transfer: 48 (21,54) days<br><br>Transfer: 12 (6,14) days<br>No transfer: 12 (7,15) days<br><br>Transfer: 42 (22,40) days<br>No transfer: 36 (17,40) days<br><br>Transfer: 4/158<br>No transfer: 11/237	Group difference: P=0.10<br><br>Group difference: P=0.80<br><br>Group difference: P=0.85<br><br>Group difference: P= 0.19<br><br>Group difference: P=0.10<br>OR 1.60 (1.09-2.7), P=0.05	Possible case-mix – not specified	No difference
Savoie et al., 2014 (27)	Paediatric Surgery Research Collaborative centres, USA: Memphis, Houston, Nashville, Little Rock, Indianapolis, Kansas	Infants with gastroschisis during 2008-2013	524 infants: 239 transferred	Nil	30-day Mortality<br><br>Length of stay†<br><br>Days to first enteral feed<br><br>Days to full enteral feeding<br><br>Total Parenteral Nutrition days	Descriptive<br>T-test<br>Chi-square<br>Multivariable analyses	Transfer: 4/239 died<br>No transfer: 4/283 died<br><br>Transfer: 42 (28,76) days<br>No transfer: 34 (24,63) days<br><br>Transfer: 18 (13,26) days<br>No transfer: 16 (11,22) days<br><br>Transfer: 31 (23,57) days<br>No transfer: 27 (20,43) days<br><br>Transfer: 29 (20,54) days<br>No transfer: 27 (17,445) days	Group difference: P=0.81<br><br>Group difference: P=0.002<br><br>Group difference: P=0.004<br><br>Group difference: P=0.0008<br><br>Group difference: P=0.06	Possible confounders – not specified	Favour birth in surgical centre/ regionalisation of services
**OESOPHAGEAL ATRESIA / TRACHEOESOPHAGEAL FISTULA**										

Confidential: For Review Only

Algert et al., 2008 (12)	New South Wales, Australia	Infants with oesophageal atresia during 2001-2003	42 infants: 25 transferred	Diagnosis made >28days after birth Antenatally diagnosable and lethal uncorrectable defects such as anencephaly, renal agenesis and trisomy 13 or 18	1-year Mortality	Test of Proportion	Transfer: 1/25 died No transfer: 3/17 died	Non-significant difference between groups	Nil	No differences
Schlee et al., 2022 (19)	Frankfurt, Germany	Infants with oesophageal atresia during 2009-2013	57 infants: 35 transferred	Nil	1-year mortality<br><br>Length of stay†<br><br>Days to full enteral feeding	Descriptive Mann-Whitney U test Fisher's exact test	Transfer: 0/35 died No transfer: 2/17 died<br><br>Transfer: 17 (13,36) days No transfer: 92 (18,158) days<br><br>Transfer: 8 (6,11) days No transfer: 12 (7,18) days	Group Difference: P= 0.103<br><br>Group difference: P= 0.009<br><br>Group difference: P=0.137	Nil	No difference
Wang et al., 2014 (28)	USA: Kids' Inpatient Database	Infants with oesophageal atresia/trachoesophageal fistula during 1997-2009	4168 infants: 1169 transferred	Nil	Mortality	Descriptive Fisher's exact test	Transfer: 99/1169 died No transfer: 186/1850 died	Group difference: P=0.399	Nil	No difference

Table 1: Summary of studies included.

*not all studies provided statistical analysis i.e., ORs, some studies only described the number of events

**not all authors provided conclusions on favoured birth location

RR- Risk Ration, aRR- adjusted Risk Ratio, OR – Odds Ratio ,aOR – adjusted Odds Ratio, P- p-value, HR hazard ratio, aHR – adjusted Hazard Ratio

† Length of stay given as median (IQR)

ς Potentially overlapping cohort: multicentre or population-based studies may include duplicates infants

Confidential: For Review Only

	Selection				Comparability		Outcome			Overall rating §
**Author, year †**	1	2	3	4	5	6	7	8	9	
Al-Shanafey (2002) (17)	*	*	*	*			*	*	*	Poor
Algert (2008) (12)	*	*	*	*			*	*	*	Poor
Aly (2010) (21)	*	*	*	*	*	*	*	*	*	Good
Boloker (2002) (22)	*	*	*	*			*	*	*	Poor
Carmichael (2020) (23)	*	*	*	*	*	*	*	*	*	Good
Dalton (2017) (25)	*	*	*	*			*	*	*	Poor
Gallot (2007) (18)	*	*	*	*	*		*	*	*	Good
Hong (2019) (26)	*	*	*	*	*		*	*	*	Good
Kandasamy (2009) (13)	*	*	*	*			*	*	*	Poor
Kitchanan (2000) (14)	*	*	*	*			*	*	*	Poor
Nagata (2013) (20)	*	*	*	*			*	*	*	Poor
Nasr (2011) (15)	*	*	*	*	*	*	*	*	*	Good
Nasr (2012) (16)	*	*	*	*	*	*	*	*	*	Good
Putnam (2016) (29)	*	*	*	*	*		*	*	*	Good
Savoie (2014) (27)	*	*	*	*	*		*	*	*	Good
Schlee (2022) (19)	*	*	*	*			*	*	*	Poor
Sola (2010) (24)	*	*	*	*	*		*	*	*	Good
Stopenski (2022) (30)	*	*	*	*	*	*	*	*	*	Good
Wang (2014) (28)	*	*	*	*			*	*	*	Poor

Table 2: Quality Assessment – Newcastle Ottawa Quality Assessment Scale for Cohort Studies

†Cohort studies: 1. representativeness of the exposed cohort, 2. selection of the non-exposed cohort, 3. ascertainment of exposure, 4. demonstration that the outcome of interest was not present at start of the study, 5. comparability of cohorts based on the design or analysis most important factor, 6. comparability of cohorts based on the design or analysis second important factor, 7. assessment of outcome, 8. was follow-up long enough for outcomes to occur and 9. adequacy of follow-up of cohort.

§Good quality rating for those with 3 or 4 stars in selection domain AND 1 or 2 stars in comparability domain AND 2 or 3 stars in outcome/exposure domain. Fair quality for 2 stars in selection domain AND 1 or 2 stars in comparability domain AND 2 or 3 stars in outcome/exposure domain. Poor quality for 0 or 1 star in selection domain OR 0 stars in comparability domain OR 0 or 1 stars in outcome/exposure domain

Confidential: For Review Only

Author/Year	Mortality	Length of Stay	Age First Enteral feed	Days to full enteral Feeding	PN Duration	Morbidity	Overall direction of effect (D) of birth in surgical centre
**CONGENITAL DIAPHRAGMATIC HERNIA**							
Al-Shanafey et al., 2002 (17)	◀▶						◀▶
Algert et al., 2008 (12)	▼						▼
Aly et al., 2010 (21)	▼ §						▼ §
Boloker et al., 2002 (22)	◀▶						◀▶
Carmichael et al., 2020 (23)	▼§						▼ §
Gallot et al., 2007 (18)	▼						▼
Nagata et al., 2013 (20)	◀▶						◀▶
Nasr and Langer, 2011 (15)	▼§						▼§
Putnam et al., 2016 (29)						◀▶§	◀▶§
Sola et al., 2010 (24)	◀▶						◀▶
Stopenski et al., 2022 (30)	◀▶§						◀▶§
**GASTROSCHISIS**							
Algert et al., 2008 (12)	◀▶						◀▶
Dalton et al., 2017 (25)		▼	▼	▼	▼		▼
Hong et al., 2019 (26)	◀▶§	◀▶§					◀▶§
Kandasamy et al., 2009 (13)	◀▶	◀▶					◀▶
Kitchanan et al., 2000 (14)			▼	▼	▼		◀▶§
Nasr and Langer, 2012 (16)	◀▶§	◀▶§	◀▶§	◀▶§	◀▶§		◀▶§
Savoie et al., (27)	◀▶§	▼§	▼§	▼§	▼§		▼§
**OESOPHAGEAL ATRESIA / TRACHEOESOPHAGEAL FISTULA**							
Algert et al., 2008 (12)	◀▶						◀▶
Schlee et al., 2022 (19)	◀▶	▼		◀▶			◀▶
Wang et al., 2014 (28)	◀▶						◀▶

Table 3: Summary of direction of effect of birth in a surgical unit

Outcomes of interest and the overall direction of effect of birth in a surgical centre compared to birth outside a surgical centre (D) showed for each study included.

Arrows denote harm (▲), benefit (▼) or conflicting evidence (◀▶).

§ denotes studies carried out case-mix adjustments

Red colour – 'Poor' quality, Yellow colour – 'Fair' quality, Green colour- 'Good' quality.

Confidential: For Review Only

Congenital diaphragmatic hernia
Eleven observational studies included babies with CDH, two were prospective whilst nine were retrospective studies (12,15,17,18,20–24,29,30). Three used single centre data to compare two populations – infants born within their unit compared to infants referred from other hospital and transferred in. The other eight studies explored data across multiple centres through patient databases and used the birth location and subsequent transfers for surgical management to define two groups. Mortality was reported in ten studies; however the definition of mortality varied – from death during admission to death in infancy. Two studies included data from multiple countries, members of the Congenital Diaphragmatic Hernia Study Group (CDHSG) (29,30). There is potential overlap in populations across studies using patient databases, however we were not able to distinguish the cohorts.

Five studies with exploring mortality favoured delivery in a surgical centre for infants with CDH– three of these rated as 'good' quality studies (Figure 2). The other five studies reported no difference in mortality. No difference in morbidity rate was reported in an international study from the CDHSG (29).

Five population-level studies carried out case-mix adjustments. Three studies of these studies, concluded that birth in surgical centre was favourable for this population, whilst two studies using international data did not find a difference in mortality after case-mix adjustments. We also found differences in the population included in the studies, some studies excluded infants with other congenital anomalies whilst other studies had no exclusion criteria – this brings selection bias and would influence the mortality rates reported. In addition, the rate of antenatal diagnosis and termination of pregnancy for congenital anomalies varies across study setting and period, which would impact the birth location and the survival rate (32,33). It is important to highlight that some authors recognised that the reported results may be skewed by 'hidden mortality', as infants may have died at delivery or prior to surgery or reaching the surgical centre and that would have impacted on the population included in their study.

Gastroschisis
Seven studies explored the impact of delivery site on the outcomes for neonates diagnosed with gastroschisis (12–14,16,25–27). All studies were single country. Three studies were rated 'good' quality and four 'poor' quality. Three multicentre studies used population level databases (16,26,27). Inclusion and exclusion criteria varied across the studies, notably one study excluded complicated gastroschisis - limiting comparison of the studies (25).

There may be overlap across USA-based cohorts, but we were unable to distinguish them. Five studies reported on mortality and all found no differences between groups (12,13,16,26,27). Median length of stay was explored in five studies. Two of these found transfers to a surgical centre was associated longer stay, these were rated as 'poor' and 'good' quality studies (25,27). The other three studies found no difference in length of stay (13,16,26). Four studies reported on duration of total parenteral nutrition, and one study, rated as 'poor' quality, found that those infants born outside a surgical centre had a longer duration whilst the others found no significant difference (14,16,25,27). One study adjusted for clustering within hospital and found no difference in mortality or length of stay (26). Two studies adjusted for case-mix of unspecified characteristics and one concluded that birth in surgical centre led to favourable outcomes, particularly length of stay (29).

Oesophageal atresia and tracheoesophageal atresia
Three included studies explored the impact of birth location and outcomes for babies diagnosed with oesophageal atresia or tracheoesophageal atresia (12,19,28). All studies used retrospective data. Algert et al., and Schlee et al., included data from a single surgical centre in Australia and Germany respectively, whilst Wang et al., used population-level data to study the population across the USA. All studies were rated 'poor' quality. No study adjusted for case-mix in their analysis. No differences in outcomes of interest – mortality, length of stay or days to full enteral feeding – were found across the studies included.

DISCUSSION:

This systematic review identified nineteen studies exploring the impact of birth location on short-term outcomes of infants born with gastroschisis, congenital diaphragmatic hernia or oesophageal atresia and tracheoesophageal atresia. All studies were observational cohort studies using data from high income to

Confidential: For Review Only

explore the impact of birth location on short-term outcomes. Study quality and risk of bias was assessed using the Newcastle-Ottawa Scale. Case-mix adjustment, not included within the quality rating, was not carried out in all studies. High study heterogeneity precluded a meta-analysis. We present a narrative synthesis with vote counting on the direction of effect. The overall direction of effect suggests limited evidence that birth in a non-surgical centre may be associated with worst outcomes particularly for increased mortality for CDH and prolonged length of stay in gastroschisis, with conflicting evidence for other outcomes and TOF/OA (Figure 2). No studies explored the impact of birth location on maternal-baby separation, and impact on breastfeeding, skin to skin or family integrated care, despite the positive impact of family integrated care on neonatal outcomes (34).

We found high heterogeneity across all included studies, and findings need to be interpreted with caution. Across all three conditions, variables that may impact outcomes such as mortality – such as associated congenital anomalies, comorbidities, surgical techniques, and surgical complications – were either not reported or not adjusted for. The included population also varied, for example studies exploring CDH, may have included complex babies with severe cardiac anomalies or genetic conditions. Complex babies are more likely to be delivered in centres with co-located services, increasing the risk of bias in our findings (12). In addition, outcome reporting was inconsistent, limiting our ability to draw conclusions - for example some studies reported mortality to discharge home whilst other reported mortality to one year of age (35). We included studies across a 20-year period, during which there have been significant improvements in fetal medicine, neonatology and surgery. This is reflected in the changes in survival rates during this time, for example CDH mortality rates have decreased by 2.4% annually since 2001 (36–39). This introduces a time-period bias when comparing the results.

Overall, we found conflicting evidence regarding the importance of location of birth for congenital surgical anomalies. There is limited evidence to suggest that birth outside a surgical centre may be associated with worst neonatal outcomes, particularly for infants with CDH which carries a high mortality rate in the first few days after birth. Similarly, requiring transfer for surgical repair of a defect such as gastroschisis may lead to delay in establishing enteral feeding and prolonged hospital stays, as found in some of the studies included. However, it is important to recognise that greater centralisation of services may also have disadvantages for families. For example, evaluation of the centralisation of congenital cardiac services in England has identified trade-offs between improved clinical outcomes and family experience – with reorganisation of care potentially widening inequalities and impacting access, with increased travel times and costs (40).

Strengths of this systematic review include the comprehensive search criteria to yield international literature from high-income countries, with no limitations on language of publication and prospective registration. In addition to published literature, we also other sources of literature and consulted experts for additional studies. We present all outcomes reported in the included studies to reduce selective outcome reporting bias. Limitations of this review includes the degree of bias discussed as well as not being able to carry out a meta-analysis of the studies included.

Future research is needed to yield concrete evidence on the impact of birth outside a surgical centre on infants with congenital surgical conditions. Using establishing routine or registry-based datasets may be the most efficient way of capturing whole-population data to explore this relationship. Analysis should adjust for case-mix, including maternal and infant characteristics. Additional outcomes, for example maternal-baby separation, as well as long-term outcomes should also be explored. Qualitative work exploring the experience of parents and carers of infants that undergo transfers postnatally will complement quantitative work and provide strong evidence to shape future design of neonatal health services.

In conclusion, we found limited evidence suggesting that birth in a non-surgical centre may contribute to prolonged length of stay and increase mortality. However, the quality of evidence was insufficient to make concrete conclusions and further research is needed.

FUNDING STATEMENT

This research is support by the National Institute for Health Research grant ACF-2020-21-011 awarded to BNM. CG was supported by the United Kingdom Medical Research Council through a Transitional Support Fellowship (MR/V036866/1). CB is supported through a UK NIHR Advanced Fellowship personal award. Imperial College London Open Access Fund supported the publication and dissemination of this work.

Confidential: For Review Only

COMPETING INTERESTS

None declared.

CONTRIBUTIONS

BNM, DH, CG and CB conceived and designed the study. BNM designed and carried out the search strategy. BNM, GS and LC reviewed search results, screened title/abstracts and full texts and extracted data from included studies. BNM and GS interpreted the results. BNM wrote the first draft and all authors contributed to the final manuscript. All authors have approved the final manuscript as submitted.

Confidential: For Review Only

References:

1. Kenny S. Getting it Right First Time: Paediatric Surgery GIRFT Programme National Specialty Report. [Internet]. NHS England and NHS Improvement; 2021 Feb [cited 2022 Apr 16]. Available from: https://www.gettingitrightfirsttime.co.uk/wp-content/uploads/2021/09/PaediatricSurgeryReport-Sept21w.pdf

2. BAPM. National Care Principles for the Management of Congenital Diaphragmatic Hernia (2018) [Internet]. British Association of Perinatal Medicine. [cited 2023 Jan 14]. Available from: https://www.bapm.org/resources/22-national-care-principles-for-the-management-of-congenital-diagphragmatic-hernia-2018

3. Snoek KG, Reiss IKM, Greenough A, Capolupo I, Urlesberger B, Wessel L, et al. Standardized Postnatal Management of Infants with Congenital Diaphragmatic Hernia in Europe: The CDH EURO Consortium Consensus - 2015 Update. Neonatology. 2016;110(1):66–74.

4. Moher D, Liberati A, Tetzlaff J, Altman DG, PRISMA Group. Preferred reporting items for systematic reviews and meta-analyses: the PRISMA statement. BMJ. 2009 Jul 21;339:b2535.

5. Registration of systematic reviews in PROSPERO: 30,000 records and counting | Systematic Reviews | Full Text [Internet]. [cited 2023 Jan 10]. Available from: https://systematicreviewsjournal.biomedcentral.com/articles/10.1186/s13643-018-0699-4

6. World Bank Country and Lending Groups – World Bank Data Help Desk [Internet]. [cited 2023 Mar 4]. Available from: https://datahelpdesk.worldbank.org/knowledgebase/articles/906519-world-bank-country-and-lending-groups

7. Webbe JWH, Duffy JMN, Afonso E, Al-Muzaffar I, Brunton G, Greenough A, et al. Core outcomes in neonatology: development of a core outcome set for neonatal research. Archives of Disease in Childhood - Fetal and Neonatal Edition. 2020 Jul 1;105(4):425–31.

8. Allin BSR, Hall NJ, Ross AR, Marven SS, Kurinczuk JJ, Knight M. Development of a gastroschisis core outcome set. Archives of Disease in Childhood - Fetal and Neonatal Edition. 2019 Jan 1;104(1):F76–82.

9. Kohl C, McIntosh EJ, Unger S, Haddaway NR, Kecke S, Schiemann J, et al. Online tools supporting the conduct and reporting of systematic reviews and systematic maps: a case study on CADIMA and review of existing tools. Environmental Evidence. 2018 Feb 1;7(1):8.

10. Ottawa Hospital Research Institute [Internet]. [cited 2023 Jan 10]. Available from: https://www.ohri.ca/programs/clinical_epidemiology/oxford.asp

11. Higgins J, Tthomas J, Chandler J, Cumpston M, Li T, Page M, et al. Cochrane Handbook for Systematic Reviews of Interventions [Internet]. [cited 2023 Jan 10]. Available from: https://training.cochrane.org/handbook

12. Algert C, Bowen J, Hadfield R, Olive E, Morris J, Roberts C. Birth at hospitals with co-located paediatric units for infants with correctable birth defects. AUSTRALIAN & NEW ZEALAND JOURNAL OF OBSTETRICS & GYNAECOLOGY. 2008;48(3):273–9.

13. Kandasamy Y, Whitehall J, Gill A, Stalewski H. Surgical management of gastroschisis in North Queensland from 1988 to 2007. JOURNAL OF PAEDIATRICS AND CHILD HEALTH. 2010;46(1–2):40–4.

14. Kitchanan S, Patole S, Muller R, Whitehall J. Neonatal outcome of gastroschisis and exomphalos: A 10-year review. JOURNAL OF PAEDIATRICS AND CHILD HEALTH. 2000;36(5):428–30.

Confidential: For Review Only

15. Nasr A, Langer J, Canadian Pediat Surg Network. Influence of location of delivery on outcome in neonates with congenital diaphragmatic hernia. JOURNAL OF PEDIATRIC SURGERY. 2011;46(5):814–6.

16. Nasr A, Langer J, Canadian Paediat Surg Network. Influence of location of delivery on outcome in neonates with gastroschisis. JOURNAL OF PEDIATRIC SURGERY. 2012;47(11):2022–5.

17. Al-Shanafey S, Giacomantonio M, Henteleff H. Congenital diaphragmatic hernia: experience without extracoporeal membrane oxygenation. PEDIATRIC SURGERY INTERNATIONAL. 2002;18(1):28–31.

18. Gallot D, Boda C, Ughetto S, Perthus I, Robert-Gnansia E, Francannet C, et al. Prenatal detection and outcome of congenital diaphragmatic hernia: a French registry-based study. Ultrasound Obstet Gynecol. 2007 Mar;29(3):276–83.

19. Schlee D, Theilen T, Fiegel H, Hutter M, Rolle U. Outcome of esophageal atresia: inborn versus outborn patients. DISEASES OF THE ESOPHAGUS. 2022;35(8).

20. Nagata K, Usui N, Kanamori Y, Takahashi S, Hayakawa M, Okuyama H, et al. The current profile and outcome of congenital diaphragmatic hernia: A nationwide survey in Japan. JOURNAL OF PEDIATRIC SURGERY. 2013;48(4):738–44.

21. Aly H, Bianco-Batlles D, Mohamed MA, Hammad TA. Mortality in infants with congenital diaphragmatic hernia: a study of the United States National Database. J Perinatol. 2010 Aug;30(8):553–7.

22. Boloker J, Bateman D, Wung J, Stolar C. Congenital diaphragmatic hernia in 120 infants treated consecutively with permissive hypercapnea/spontaneous respiration/elective repair. JOURNAL OF PEDIATRIC SURGERY. 2002;37(3):357–65.

23. Carmichael S, Ma C, Lee H, Shaw G, Sylvester K, Hintz S. Survival of infants with congenital diaphragmatic hernia in California: impact of hospital, clinical, and sociodemographic factors. JOURNAL OF PERINATOLOGY. 2020;40(6):943–51.

24. Sola J, Bronson S, Cheung M, Ordonez B, Neville H, Koniaris L. Survival disparities in newborns with congenital diaphragmatic hernia: a national perspective. JOURNAL OF PEDIATRIC SURGERY. 2010;45(6):1336–42.

25. Dalton B, Gonzalez K, Reddy S, Hendrickson R, Iqbal C. Improved outcomes for inborn babies with uncomplicated gastroschisis. JOURNAL OF PEDIATRIC SURGERY. 2017;52(7):1132–4.

26. Hong C, Fullerton B, Han S, Morrow K, Edwards E, Soll R, et al. Impact of disease-specific volume and hospital transfer on outcomes in gastroschisis. JOURNAL OF PEDIATRIC SURGERY. 2019;54(1):65–9.

27. Savoie K, Huang E, Aziz S, Blakely M, Dassinger S, Dorale A, et al. Improving gastroschisis outcomes: Does birth place matter? JOURNAL OF PEDIATRIC SURGERY. 2014;49(12):1771–5.

28. Wang B, Tashiro J, Allan B, Sola J, Parikh P, Hogan A, et al. A nationwide analysis of clinical outcomes among newborns with esophageal atresia and tracheoesophageal fistulas in the United States. JOURNAL OF SURGICAL RESEARCH. 2014;190(2):604–12.

29. Putnam L, Harting M, Tsao K, Morini F, Yoder B, Luco M, et al. Congenital Diaphragmatic Hernia Defect Size and Infant Morbidity at Discharge. PEDIATRICS. 2016;138(5).

30. Stopenski S, Guner Y, Jolley J, Major C, Hatfield T, Ebanks A, et al. Inborn Versus Outborn Delivery in Neonates With Congenital Diaphragmatic Hernia. JOURNAL OF SURGICAL RESEARCH. 2022;270:245–51.

31. Murphy F, Mazlan T, Tarheen F, Corbally M, Puri P. Gastroschisis and exomphalos in Ireland 1998-2004. Does antenatal diagnosis impact on outcome? PEDIATRIC SURGERY INTERNATIONAL. 2007;23(11):1059–63.

Confidential: For Review Only

32. Aldridge N, Pandya P, Rankin J, Miller N, Broughan J, Permalloo N, et al. Detection rates of a national fetal anomaly screening programme: A national cohort study. BJOG. 2023 Jan;130(1):51–8.

33. Morris JK, Springett AL, Greenlees R, Loane M, Addor MC, Arriola L, et al. Trends in congenital anomalies in Europe from 1980 to 2012. PLOS ONE. 2018 Apr 5;13(4):e0194986.

34. O'Brien K, Robson K, Bracht M, Cruz M, Lui K, Alvaro R, et al. Effectiveness of Family Integrated Care in neonatal intensive care units on infant and parent outcomes: a multicentre, multinational, cluster-randomised controlled trial. Lancet Child Adolesc Health. 2018 Apr;2(4):245–54.

35. Webbe JWH, Ali S, Sakonidou S, Webbe T, Duffy JMN, Brunton G, et al. Inconsistent outcome reporting in large neonatal trials: a systematic review. Arch Dis Child Fetal Neonatal Ed. 2020 Jan;105(1):69–75.

36. Bhatt P, Poku FA, Umscheid J, Ayensu M, Parmar N, Vasudeva R, et al. Trends in prevalence and mortality of gastroschisis and omphalocele in the United States from 2010 to 2018. World J Pediatr. 2022 Jul 1;18(7):511–4.

37. Tan Tanny SP, Beck C, King SK, Hawley A, Brooks JA, McLeod E, et al. Survival Trends and Syndromic Esophageal Atresia. Pediatrics. 2021 May;147(5):e2020029884.

38. Politis MD, Bermejo-Sánchez E, Canfield MA, Contiero P, Cragan JD, Dastgiri S, et al. Prevalence and mortality in children with congenital diaphragmatic hernia: a multicountry study. Annals of Epidemiology. 2021 Apr 1;56:61-69.e3.

39. Glinianaia SV, Rankin J, Pierini A, Coi A, Santoro M, Tan J, et al. Ten-Year Survival of Children With Congenital Anomalies: A European Cohort Study. Pediatrics. 2022 Feb 11;149(3):e2021053793.

40. Safe_and_Sustainable_Review_of_Childrens_Congenital_Cardiac_Services_in_England_Pre_Consultation_Business_Case.pdf [Internet]. [cited 2023 Feb 1]. Available from: https://www.chfed.org.uk/wp-content/uploads/2012/06/Safe_and_Sustainable_Review_of_Childrens_Congenital_Cardiac_Services_in_England_Pre_Consultation_Business_Case.pdf

Confidential: For Review Only

Figure 1: PRISMA flowchart

Figure 2: Harvest plot of overall direction-of-effect in included studies by overall quality score.
Height depicts overall quality judgement and colour depicts surgical conditions (blue = CDH; yellow = gastroschisis; orange=TOF/OA).
Arrows denote adverse effects (↑), beneficial effects (↓) or conflicting evidence (⇔) of birth in a surgical unit.

Confidential: For Review Only

Confidential: For Review Only

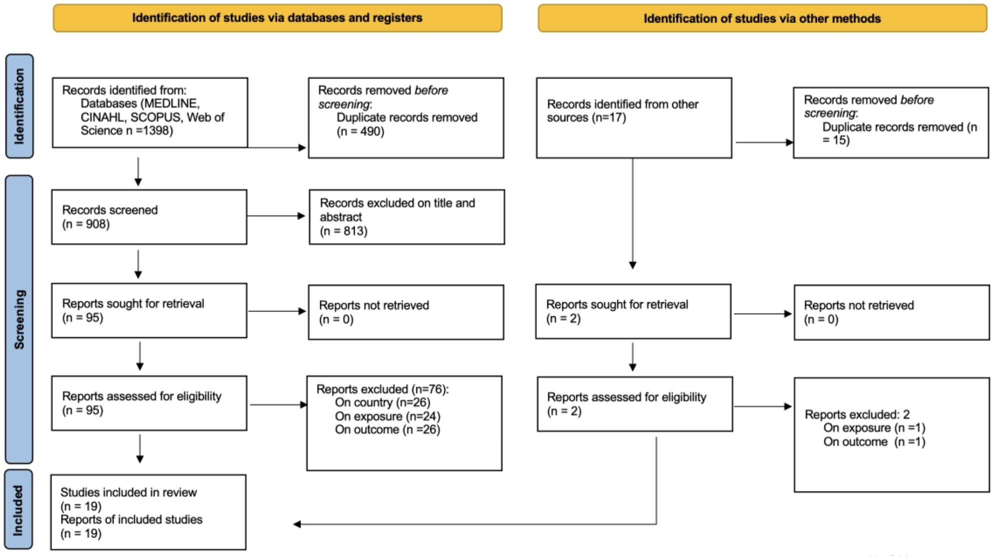

Figure 1: PRISMA flowchart

403x227mm (144 x 144 DPI)

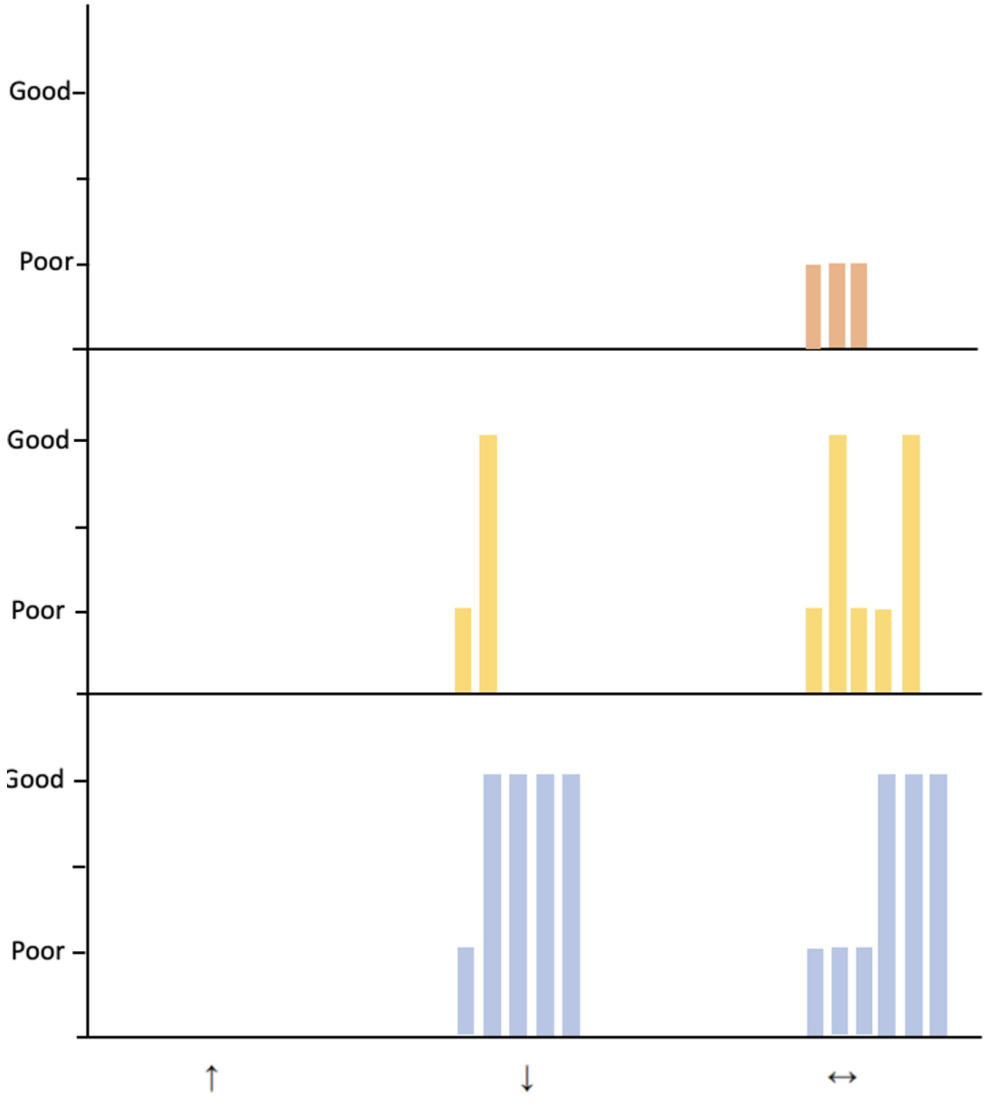

Figure 2: Harvest plot of overall direction-of-effect in included studies by overall quality score.
Height depicts overall quality judgement and colour depicts surgical conditions (blue = CDH; yellow = gastroschisis; orange=TOF/OA).
Arrows denote adverse effects (↑), beneficial effects (↓) or conflicting evidence (⇔) of birth in a surgical unit.

194x220mm (144 x 144 DPI)

Supplementary Material 1:

Table S1: Example Search Strategy

Search string	Database or further sources	Results	Date	Comments
((gastroschisis) OR (oesophageal atresia) OR (congenital diaphragmatic hernia)) AND ((location) OR (birth place) OR (site of delivery)) OR (transfer) OR (inborn) OR (outborn)	MEDLINE	192	2022-05-06	
MM "Gastroschisis") OR "gastroschisis" OR congenital diaphragmatic hernia OR congenital diaphragmal hernia OR tracheoesophageal fistula OR esophageal atresia AND ((location) OR (birth place) OR (site of delivery)) OR (transfer) OR (inborn) OR (outborn)	CINAHL	97	2022-05-06	
(((((ALL=(location)) OR ALL=(birthplace)) OR ALL=(site of delivery)) OR ALL=(inborn)) OR ALL=(outworn)) OR ALL=(transfer) AND (((ALL=(gastroschisis)) OR ALL=(congenital diaphragmatic hernia)) OR ALL=(tracheoesophageal fistula)) OR ALL=(esophageal atresia)	Web of Science	481	2022-05-06	
TITLE-ABS-KEY (((gastroschisis) OR (oesophageal AND atresia) OR (congenital AND diaphragmatic AND hernia)) AND ((location) OR (birth AND place) OR (site AND of AND delivery)) OR (transfer) OR (inborn) OR (outborn))	SCOPUS	529	2022-05-06	
((gastroschisis) OR (oesophageal atresia) OR (congenital diaphragmatic hernia)) AND ((location) OR (birth place) OR (site of delivery)) OR (transfer) OR (inborn) OR (outborn)	MEDLINE	10	2023-01-10	
(((((ALL=(location)) OR ALL=(birthplace)) OR ALL=(site of delivery)) OR ALL=(inborn)) OR ALL=(outworn)) OR ALL=(transfer) AND (((ALL=(gastroschisis)) OR ALL=(congenital diaphragmatic hernia)) OR ALL=(tracheoesophageal fistula)) OR ALL=(esophageal atresia)	Web of Science	35	2023-01-11	
TITLE-ABS-KEY (((gastroschisis) OR (oesophageal AND atresia) OR (congenital AND diaphragmatic AND hernia)) AND ((location) OR (birth AND place) OR (site AND of AND delivery)) OR (transfer) OR (inborn) OR (outborn))	SCOPUS	45	2023-01-11	
MM "Gastroschisis") OR "gastroschisis" OR congenital diaphragmatic hernia OR congenital diaphragmal hernia OR tracheoesophageal fistula OR esophageal atresia AND ((location) OR (birth place) OR (site of delivery)) OR (transfer) OR (inborn) OR (outborn)	CINAHL	9	2023-01-11	

Table S2: Excluded on Full Text Screening

Title	Author	Reason Exclusion
A single-center observational study on congenital diaphragmatic hernia: Outcome, predictors of mortality and experience from a tertiary perinatal center in Singapore.	Teo, Wan-Yee; Sriram, Bhavani; Alim, Aa Abdul; Ruan, Xucong; Rajadurai, V S	Details on whether transfer required? (Comparator);
Management and outcome of neonates with a prenatal diagnosis of esophageal atresia type A: A population-based study.	Garabedian, C; Bonnard, A; Rousseau, V; Sfeir, R; CRACMO; Drumez, E; Michaud, L; Gottrand, F; Houfflin-Debarge, V	Details on whether transfer required? (Comparator);
Survival of outborns with congenital diaphragmatic hernia: the role of protective ventilation, early presentation and transport distance: a retrospective cohort study.	Bojanic, Katarina; Pritisanac, Ena; Luetic, Tomislav; Vukovic, Jurica; Sprung, Juraj; Weingarten, Toby N; Carey, William A; Schroeder, Darrell R; Grizelj, Ruza	Details on whether transfer required? (Comparator);
[Influence of selected factors on the treatment and prognosis in newborns with gastroschisis on the basis of own experience].	Sawicka, Ewa; Wieprzowski, Lukasz; Jaczynska, Renata; Maciejewski, Tomasz	Details on whether transfer required? (Comparator);
Effect of time to surgical evaluation on the outcomes of infants with gastroschisis.	Bucher, Brian T; Mazotas, Ioanna G; Warner, Brad W; Saito, Jacqueline M	Details on whether transfer required? (Comparator);
Gastroschisis: a multi-centre comparison of management and outcome.	Manson, Joanna; Ameh, Emmanuel; Canvassar, Noel; Chen, Tiffany; den Hoeve, A Van; Lever, F; Hesse, Afua; Millar, Alastair; Emil, Sherif; Ade-Ajayi, Niyi	Details on whether transfer required? (Comparator);
[Congenital gastroschisis--prenatal diagnosis and perinatal management].	Weichert, J; Kahl, F O; Schroer, A; Bohlmann, M K; Diedrich, K; Hartge, D R	Details on whether transfer required? (Comparator);
Management and outcome for babies born with a gastroschisis.	Williamson, Sarah Louise; Lawrence, Louise; Arul, G. Suren; Rasiah, Shree Vishna	Details on exposure?

Confidential: For Review Only

Study of 24 cases with congenital esophageal atresia: what are the risk factors?	Sugito K; Koshinaga T; Hoshino M; Inoue M; Goto H; Ikeda T; Hagiwara N	Details on whether transfer required? (Comparator);
A 20-year experience on neonatal extracorporeal membrane oxygenation in a referral center.	Schaible T; Hermle D; Loersch F; Demirakca S; Reinshagen K; Varnholt V; Schaible, T; Hermle, D; Loersch, F; Demirakca, S; Reinshagen, K; Varnholt, V	Details on whether transfer required? (Comparator);
Predictive factors for complications in children with esophageal atresia and tracheoesophageal fistula	Shah, R; Varjavandi, V; Krishnan, U	Details on whether transfer required? (Comparator);
Congenital diaphragmatic hernia - Results of an ECMO-centre	Dahlheim, M; Witsch, M; Demirakca, S; Lorenz, C; Schaible, T	Details on whether transfer required? (Comparator);
Congenital diaphragmatic hernia: A local experience	Khawahur, H; Kattan, A; Al-Alaiyan, S; Saidy, K	Details on whether transfer required? (Comparator);
Congenital diaphragmatic hernia: a modern day approach	Waag, KL; Loff, S; Zahn, K; Ali, M; Hien, S; Kratz, M; Neff, W; Schaffeder, R; Schaible, T	Details on whether transfer required? (Comparator);
Outcomes of Congenital Diaphragmatic Hernia: An 8-Year Experience	Fallahi, M; Mohajerzadeh, L; Borhani, S; Kazemian, M; Roozroukh, M; Khaleghnejad-Tabari, A; Azma, R; Mahdavi, A	Details on whether transfer required? (Comparator);
Outcomes of newborns with gastroschisis: The effects of mode of delivery, site of delivery, and interval from birth to surgery	Quirk, JG; Fortney, J; Collins, HB; West, J; Hassad, SJ; Wagner, C	
Congenital diaphragmatic hernia - a Belgrade single center experience	Kalanj, J; Salevic, P; Rsovac, S; Medjo, B; Antunovic, SS; Simic, D	Details on whether transfer required? (Comparator);
Congenital diaphragmatic hernia: a survey of practice in Scandinavia	Skari, H; Bjornland, K; Frenckner, B; Friberg, LG; Heikkinen, M; Hurme, T; Loe, B; Mollerlokken, G; Nielsen, OH; Qvist, N; Rintala, R; Sandgren, K; Serlo, W; Wagner, K; Wester, T; Emblem, R	Details on whether transfer required? (Comparator);
Survival of outborns with congenital diaphragmatic hernia: the role of protective ventilation, early presentation and transport distance: a retrospective cohort study	Bojanic, K; Pritisanac, E; Luetic, T; Vukovic, J; Sprung, J; Weingarten, TN; Carey, WA; Schroeder, DR; Grizelj, R	Details on whether transfer required? (Comparator);
A population-based study of congenital diaphragmatic hernia: Impact of associated anomalies and preoperative blood gases on survival	Kaiser, JR; Rosenfeld, CR	Details on whether transfer required? (Comparator);
Outcomes of congenital diaphragmatic hernia: A population-based study in western Australia	Colvin, J; Bower, C; Dickinson, JE; Sokol, J	Details on whether transfer required? (Comparator);
Outcomes in the physiologically most severe congenital diaphragmatic hernia (CDH) patients: Whom should we treat?	Kays, DW; Islam, S; Perkins, JM; Larson, SD; Taylor, JA; Talbert, JL	Details on whether transfer required? (Comparator);
Congenital diaphragmatic hernia: outcome review of 2,173 surgical repairs in US infants	Abdullah, F; Zhang, YY; Sciortino, C; Camp, M; Gabre-Kidan, A; Price, MR; Chang, DC	Details on whether transfer required? (Comparator);
Congenital Diaphragmatic Hernia Defect Size and Infant Morbidity at Discharge	Putnam, LR; Harting, MT; Tsao, K; Morini, F; Yoder, BA; Luco, M; Lally, PA; Lally, KP	Details on whether transfer required? (Comparator);
Analysis of 29 consecutive thoracoscopic repairs of congenital diaphragmatic hernia in neonates compared to historical controls	Cho, SD; Krishnaswami, S; Mckee, JC; Zallen, G; Silen, ML; Bliss, DW	Details on whether transfer required? (Comparator);
CONGENITAL DIAPHRAGMATIC-HERNIA - UPDATE ON REGIONAL EXPERIENCE	ALUMRAN, K; KHAWAJA, S; DAWODU, AH; ALARFJ, A	Details on whether transfer required? (Comparator);
IS SPECIALIST CENTER DELIVERY OF GASTROSCHISIS BENEFICIAL	NICHOLLS, G; UPADHYAYA, V; GORNALL, P; BUICK, RG; CORKERY, JJ	Details on whether transfer required? (Comparator);
Practice variation in gastroschisis: Factors influencing closure technique	Stanger, J; Mohajerani, N; Skarsgard, ED	Details on whether transfer required? (Comparator);
Delivery in a tertiary Center with co-located surgical facilities makes the difference among neonates with prenatally diagnosed major abnormalities	Calisti, A; Oriolo, L; Giannino, G; Spagnol, L; Molle, P; Buffone, EL; Donadio, C	Known birth location and whether surgical services are available on site (Intervention/Exposure)
INFLUENCE OF PLACE OF DELIVERY ON OUTCOME IN BABIES WITH GASTROSCHISIS	STOODLEY, N; SHARMA, A; NOBLETT, H; JAMES, D	Known birth location and whether surgical services are available on site (Intervention/Exposure)
Gastroschisis outcomes and site of delivery	Robilio, D; Greve, L; Towner, D	Known birth location and whether surgical services are available on site

Confidential: For Review Only

		(Intervention/Exposure)
Mode of delivery and neonatal survival of infants with gastroschisis in Australia and New Zealand	Abdel-Latif, ME; Bolisetty, S; Abeywardana, S; Lui, K	Details on whether transfer required? (Comparator);
Factors Associated with Mortality in Neonates with Gastroschisis	Clark, RH; Walker, MW; Gauderer, MWL	Known birth location and whether surgical services are available on site (Intervention/Exposure)
Perinatal demography of gastroschisis in North Queensland	Whitehall, JS; Kandasamy, Y; Stalewski, H; Gill, A	Details on whether transfer required? (Comparator);
An Investigation of Racial-Ethnic Disparities in Location of Delivery, Hospital Transfer, and Survival Among Infants with Congenital Diaphragmatic Hernia	Carmichael, SL; Ma, C; Shaw, GM; Sylvester, KG; Hintz, SR	Known birth location and whether surgical services are available on site (Intervention/Exposure)
Gastroschisis and exomphalos in Ireland 1998-2004. Does antenatal diagnosis impact on outcome?	Murphy, FL; Mazlan, TA; Tarheen, F; Corbally, MT; Puri, P	Details on whether transfer required? (Comparator);
Trends in incidence and outcomes of gastroschisis in the United States: analysis of the national inpatient sample 2010-2014	Bhatt, P; Lekshminarayanan, A; Donda, K; Dapaah-Siakwan, F; Thakkar, B; Parat, S; Chabra, S; Billimoria, Z	Details on whether transfer required? (Comparator);
Complications of esophageal strictures dilatation in children A tertiary-center experience	Bawazir, O; Almaimani, MO	Details on whether transfer required? (Comparator);
The Effect of Level of Care on Gastroschisis Outcomes	Apfeld, JC; Kastenberg, ZJ; Sylvester, KG; Lee, HC	Details on whether transfer required? (Comparator);
Outcomes of newborns with gastroschisis: The effects of mode of delivery, site of delivery, and interval from birth to surgery - Discussion	Niebyl, JR; Woods, JR; Evans, MI; Queenan, JT; Carpenter, MW; Merkatz, IR; Quirk	Details on whether transfer required? (Comparator);
CONTROVERSIES IN THE MANAGEMENT OF GASTROSCHISIS - A STUDY OF 40 PATIENTS	STRINGER, MD; BRERETON, RJ; WRIGHT, VM	Details on whether transfer required? (Comparator);
Length of stay and cost analysis of neonates undergoing surgery at a tertiary neonatal unit in England	Shetty, S; Kennea, N; Desai, P; Giuliani, S; Richards, J	Details on whether transfer required? (Comparator);
Considerations for delivery of infants with congenital abnormalities	Wilkins-Haug, L	Details on whether transfer required? (Comparator);
Timing and location of delivery of the fetus with gastroschisis	Leuthner, SR; Wigton, T; Brown, AM; Sweeney, B; Sato, T; Aiken, J	Details on whether transfer required? (Comparator);
A systematic review and meta-analysis of the impact of location of delivery on outcomes in infants with gastroschisis	Nitsche, JF; Li, Y; Brost, B	Details on whether transfer required? (Comparator);
Prenatal diagnosis and the pediatric surgeon: The impact of prenatal consultation on perinatal management	Crombleholme, TM; DAlton, M; Cendron, M; Alman, B; Goldberg, MD; Klauber, GT; Cohen, A; Heilman, C; Lewis, M; Harris, BH	Details on whether transfer required? (Comparator);
Transfer and treatment mechanism and practical effect analysis of prenatally diagnosed congenital dia-phragmatic hernia	Ying, W.; Lishan, M.; Chao, L.; Yandong, W.; Jingna, L.; Yunlong, Z.; Yanxia, Z.; Rui, Z.	Details on whether transfer required? (Comparator);
The financial burden of surgery for congenital malformations—the austrian perspective	Gasparella, P.; Singer, G.; Kienesberger, B.; Arneitz, C.; Fülöp, G.; Castellani, C.; Till, H.; Schalamon, J.	Details on whether transfer required? (Comparator);
International recommendations on neonatal management with congenital diaphragmatic hernia: literature review	Babintseva, A.G.; Godovanets, Yu.D.; Khodzinska, Yu.Yu.; Popeliuk, N.O.; Koshurba, I.V.	Details on whether transfer required? (Comparator);
Clinical experience of ex-utero intrapartum treatment and prognostic factors of neonatal diaphragmatic hernia	Yunlong, Z.; Lishuang, M.; Yidin, J.; Ying, W.; Chao, L.; Yandong, W.; Jingna, L.; Yan, L.; Shan, J.; Hua, M.; Yunshu, O.; Feng, F.; Huf, Y.; Lijian, P.	Details on whether transfer required? (Comparator);
Effect of multidisciplinary treatment on outcomes in infants with congenital diaphragmatic hernia: 10-year experience	Pan, W.; Wang, W.; Wang, J.; Xie, W.; Wang, Y.; Jiang, Y.	Details on whether transfer required? (Comparator);
Prenatal diagnoses and outcomes of congenital diaphragmatic hernia.	Laye, M.R.; Rehberg, J.F.; Kosek, M.A.; Bufkin, L.K.; Bofill, J.A.	Details on whether transfer required? (Comparator);
Risk factors for mortality of newborns with gastroschisis in a tertiary hospital of Taiwan	Zhao, L.-L.; Chang, C.-J.; Chou, H.-C.; Tsao, P.-N.; Hsieh, W.-S.	Details on whether transfer required? (Comparator);
Gastroschisis: Determinants of neonatal outcome	Singh, S.J.; Fraser, A.; Leditschke, J.F.; Spence, K.; Kimble, R.; Dalby-Payne, J.;	Details on whether transfer required? (Comparator);

Confidential: For Review Only

	Baskaranathan, S.; Barr, P.; Halliday, R.; Badawi, N.; Peat, J.K.; Glasson, M.; Cass, D.	
Congenital diaphragmatic hernia. Prenatal diagnosis and neonatal outcome	Lamberti, A.; Liguori, M.; Teodoro, A.; Tartaglione, A.; Caccioppoli, U.; Paladini, D.	Known birth location and whether surgical services are available on site (Intervention/Exposure)
Current status of antenatal diagnosis & perinatal medical network	Suita, S.	Details on whether transfer required? (Comparator);
Exomphalos and gastroschisis, a study of fetal and neonatal outcome in Wales 1974-1987	Evans, J.; Madarikan, B.A.; Lari, J.	Details on whether transfer required? (Comparator);
Anterior abdominal wall defects	Lafferty, P.M.; Emmerson, A.J.; Fleming, P.J.; Frank, J.D.; Noblett, H.R.	Known birth location and whether surgical services are available on site (Intervention/Exposure)
Development of a program for planned cesarean delivery and immediate pediatric surgical repair of ventral wall defects	Evans, M.I.; Drugan, A.; Greenholz, S.K.; Hauff, N.; Jewell, M.R.; Klein, M.D.	Details on whether transfer required? (Comparator);
Congenital diaphragmatic hernia: a 20 year experience	Simson, J.N.L.; Eckstein, H.B.	Details on whether transfer required? (Comparator);
Prenatal diagnosis and natural history of the fetus with a congenital diaphragmatic hernia: Initial clinical experience	Nakayama, D.K.; Harrison, M.R.; Chinn, D.H.; Callen, P.W.; Filly, R.A.; Golbus, M.S.; De Lorimier, A.A.	Known birth location and whether surgical services are available on site (Intervention/Exposure)
Perinatal management of gastroschisis	Fitzsimmons, J.; Nyberg, D. A.; Cyr, D. R.; Hatch, E.	Known birth location and whether surgical services are available on site (Intervention/Exposure)
Clinical profile, outcomes and predictors of mortality in neonates operated for gastrointestinal anomalies in a tertiary neonatal care unit- An observational study	Jerry, A.L.; Amboiram, P.; Balakrishnan, U.; Chandrasekaran, A.; Agarwal, P.; Devi, U.	Known birth location and whether surgical services are available on site (Intervention/Exposure)
Prenatal diagnoses and outcomes of congenital diaphragmatic hernia.	Laye, M.R.; Rehberg, J.F.; Kosek, M.A.; Bufkin, L.K.; Bofill, J.A.	Known birth location and whether surgical services are available on site (Intervention/Exposure)
Risk factors for mortality of newborns with gastroschisis in a tertiary hospital of Taiwan	Zhao, L.-L.; Chang, C.-J.; Chou, H.-C.; Tsao, P.-N.; Hsieh, W.-S.	Known birth location and whether surgical services are available on site (Intervention/Exposure)
Risk of need for extracorporeal membrane oxygenation support in neonates with congenital diaphragmatic hernia treated with inhaled nitric oxide	Sebald, M.; Friedlich, P.; Burns, C.; Stein, J.; Noori, S.; Ramanathan, R.; Seri, I.	Known birth location and whether surgical services are available on site (Intervention/Exposure)
Gastroschisis: Determinants of neonatal outcome	Singh, S.J.; Fraser, A.; Leditschke, J.F.; Spence, K.; Kimble, R.; Dalby-Payne, J.; Baskaranathan, S.; Barr, P.; Halliday, R.; Badawi, N.; Peat, J.K.; Glasson, M.; Cass, D.	Known birth location and whether surgical services are available on site (Intervention/Exposure)
Congenital diaphragmatic hernia. Prenatal diagnosis and neonatal outcome	Lamberti, A.; Liguori, M.; Teodoro, A.; Tartaglione, A.; Caccioppoli, U.; Paladini, D.	Known birth location and whether surgical services are available on site (Intervention/Exposure)
Current status of antenatal diagnosis & perinatal medical network	Suita, S.	Details on whether transfer required? (Comparator);
Exomphalos and gastroschisis, a study of fetal and neonatal outcome in Wales 1974-1987	Evans, J.; Madarikan, B.A.; Lari, J.	Details on whether transfer required? (Comparator);
Anterior abdominal wall defects	Lafferty, P.M.; Emmerson, A.J.; Fleming, P.J.; Frank, J.D.; Noblett, H.R.	Known birth location and whether surgical services are available on site (Intervention/Exposure)
Development of a program for planned cesarean delivery and immediate pediatric surgical repair of ventral wall defects	Evans, M.I.; Drugan, A.; Greenholz, S.K.; Hauff, N.; Jewell, M.R.; Klein, M.D.	Details on whether transfer required? (Comparator);
Congenital diaphragmatic hernia: a 20 year experience	Simson, J.N.L.; Eckstein, H.B.	Details on whether transfer required? (Comparator);
Prenatal diagnosis and natural history of the fetus with a congenital diaphragmatic hernia: Initial clinical experience	Nakayama, D.K.; Harrison, M.R.; Chinn, D.H.; Callen, P.W.; Filly, R.A.; Golbus, M.S.; De Lorimier, A.A.	Details on whether transfer required? (Comparator);
Perinatal management of gastroschisis	Fitzsimmons, J.; Nyberg, D. A.; Cyr, D. R.; Hatch, E.	Details on whether transfer required? (Comparator);
Clinical profile, outcomes and predictors of mortality in neonates operated for gastrointestinal anomalies in a tertiary neonatal care unit- An observational study	Jerry, A.L.; Amboiram, P.; Balakrishnan, U.; Chandrasekaran, A.; Agarwal, P.; Devi, U.	Details on whether transfer required? (Comparator);

Confidential: For Review Only

Confidential: For Review Only

BMJ Paediatrics Open

Association between birth location and short-term outcomes for babies with gastroschisis, congenital diaphragmatic hernia and oesophageal fistula: a systematic review

Journal:	*BMJ Paediatrics Open*
Manuscript ID	bmjpo-2023-002007.R1
Article Type:	Original research
Date Submitted by the Author:	16-May-2023
Complete List of Authors:	Nezafat Maldonado, Behrouz; Imperial College London, Neonatal Medicine Chelsea and Westminster Hospital campus<br>Singhal, Graciaa; Hull York Medical School<br>Chow, LiYan; Chelsea and Westminster Hospital NHS Foundation Trust, Neonatal Medicine Department<br>Hargreaves, Dougal; Imperial College London School of Public Health<br>Gale, Chris; Imperial College London, Section of Neonatal Medicine<br>Battersby, Cheryl; Imperial College London, Neonatal Medicine Chelsea and Westminster Hospital campus
Keywords:	Neonatology, Health services research

SCHOLARONE™
Manuscripts

I, the Submitting Author has the right to grant and does grant on behalf of all authors of the Work (as defined in the below author licence), an exclusive licence and/or a non-exclusive licence for contributions from authors who are: i) UK Crown employees; ii) where BMJ has agreed a CC-BY licence shall apply, and/or iii) in accordance with the terms applicable for US Federal Government officers or employees acting as part of their official duties; on a worldwide, perpetual, irrevocable, royalty-free basis to BMJ Publishing Group Ltd ("BMJ") its licensees and where the relevant Journal is co-owned by BMJ to the co-owners of the Journal, to publish the Work in this journal and any other BMJ products and to exploit all rights, as set out in our licence.

The Submitting Author accepts and understands that any supply made under these terms is made by BMJ to the Submitting Author unless you are acting as an employee on behalf of your employer or a postgraduate student of an affiliated institution which is paying any applicable article publishing charge ("APC") for Open Access articles. Where the Submitting Author wishes to make the Work available on an Open Access basis (and intends to pay the relevant APC), the terms of reuse of such Open Access shall be governed by a Creative Commons licence – details of these licences and which Creative Commons licence will apply to this Work are set out in our licence referred to above.

Other than as permitted in any relevant BMJ Author's Self Archiving Policies, I confirm this Work has not been accepted for publication elsewhere, is not being considered for publication elsewhere and does not duplicate material already published. I confirm all authors consent to publication of this Work and authorise the granting of this licence.

Confidential: For Review Only

Association between birth location and short-term outcomes for babies with gastroschisis, congenital diaphragmatic hernia and oesophageal fistula: a systematic review

B Nezafat Maldonado[1], G Singhal[2], L Chow[3], D Hargreaves[4], C Gale[1], C Battersby[1]

1. Neonatal Medicine, School of Public Health, Faculty of Medicine, Imperial College London, Chelsea & Westminster Hospital campus, London, United Kingdom
2. Hull York Medical School,
3. Neonatal Medicine Department, Chelsea and Westminster NHS Foundation Trust
4. Mohn Centre for Children's Health and Wellbeing, Imperial College London

Corresponding author:
Dr Behrouz Nezafat Maldonado
Neonatal Medicine
School of Public Health
Imperial College London
Chelsea and Westminster Hospital Campus
S W10 9NH
bnezafat@imperial.ac.uk

Dr Behrouz Nezafat Maldonado
Neonatal Medicine, School of Public Health, Faculty of Medicine, Imperial College London, Chelsea & Westminster Hospital campus, London, United Kingdom

Ms Graciaa Singhal
Hull York Medical Schol

Dr Dougal Hargreaves
Mohn Centre for Children's Health and Wellbeing, Imperial College London

Professor Chris Gale
Neonatal Medicine, School of Public Health, Faculty of Medicine, Imperial College London, Chelsea & Westminster Hospital campus, London, United Kingdom

Dr Cheryl Battersby
Neonatal Medicine, School of Public Health, Faculty of Medicine, Imperial College London, Chelsea & Westminster Hospital campus, London, United Kingdom

Confidential: For Review Only

Key words: Neonatology
Word count: 2601 excluding title page, abstract, references, figures and tables

ABSTRACT

Background

Neonatal care is commonly regionalised, meaning specialist services are only available at certain units. Consequently, infants with surgical conditions needing specialist care that are born in non-surgical centres require postnatal transfer. Best practice models advocate for co-located maternity and surgical services as the place of birth for infants with antenatally diagnosed congenital conditions to avoid postnatal transfers. We conducted a systematic review to explore the association between location of birth and short-term outcomes of babies with gastroschisis, congenital diaphragmatic hernia (CDH) and oesophageal atresia with or without tracheoesophageal fistula (TOF/OA).

Methods

We searched MEDLINE, CINAHL, Web of Science and SCOPUS databases for studies from high income countries comparing outcomes for infants with gastroschisis, CDH or TOF/OA based on their place of delivery. Outcomes of interest included mortality, length of stay, age at first feed, comorbidities, and duration of parenteral nutrition. We assessed study quality using the Newcastle-Ottawa Scale. We present a narrative synthesis of our findings.

Results

Nineteen cohort studies compared outcomes of babies with one of gastroschisis, CDH or TOF/OA. Heterogeneity across the studies precluded meta-analysis. Eight studies carried out case-mix adjustments. Overall, we found conflicting evidence. There is limited evidence to suggest that birth in a maternity unit with a co-located surgical centre was associated with a reduction in mortality for CDH and decreased length of stay for gastroschisis.

Conclusions

There is little evidence to suggest that delivery in co-located maternity-surgical services may be associated with shortened length of stay and reduced mortality. Our findings are limited by significant heterogeneity, potential for bias and paucity of strong evidence. This supports the need for further research to investigate the impact of birth location on outcomes for babies with congenital surgical conditions and inform future design of neonatal care systems.

Review registration PROSPERO Registration number: CRD42022329090

What is already known on this topic

Infants with congenital surgical anomalies require specialist surgical care and neonatal care. This specialist care may not be available across all units. As a result, some infants are delivered in a non-surgical centre and are then postnatally transferred to a surgical centre. International consensus recommends delivery in co-located maternity and surgical units for babies with antenatally diagnosed surgical conditions.

What this study adds

We systematically identified international data exploring the impact of birth in a non-surgical centre on neonatal outcomes for infants with CDH, gastroschisis or TOF/OA. There is limited evidence to suggest that birth in a non-surgical centre may be associated with increased mortality, prolonged length of stay and delayed enteral feeding.

How this study might affect research, practice or policy

This study highlights key gaps in the evidence; studies able to account for case-mix and clinical severity and reporting on core outcomes across different birth locations are needed to inform future development of neonatal care systems.

Confidential: For Review Only

INTRODUCTION:

Infants with congenital surgical conditions require specialist surgical input soon after birth. It is important to ensure that specialist services are available to adequately care for these babies. Neonatal care may be delivered through centralised or decentralised models. This means that different levels of care may be available at birth across health care settings.

Antenatal care aims to identify congenital anomalies, for optimal delivery planning with access to specialist care. Guidelines recommend delivery in a centre with neonatal intensive care and neonatal surgical services located within the same facility for infants with suspected surgical anomalies (1–3). However, due to socioeconomic, environmental or health factors, or the nature of the defect anomalies are not identified antenatally, and infants are born in a unit without surgical services. Postnatal transfer can hinder parental access and bonding with their baby, as complex infants may be cared for in specialist centres far from the family residence. Clinical pathways should enable provision of intensive care to all infants where this is required, while minimising postnatal transfer (3).

To improve neonatal surgical care, we must understand how birthplace impacts outcomes for congenital anomalies. Some anomalies, such as gastroschisis, are often diagnosed antenatally, while others like tracheoesophageal fistula or oesophageal atresia (TOF/OA) are not. Other defects such as congenital diaphragmatic hernia (CDH) may be antenatally diagnosed and require intensive care from birth, meaning delivery in the right place can be key. As a result, planning the place of delivery varies based on the condition. Gastroschisis, tracheoesophageal fistula or oesophageal atresia and congenital diaphragmatic hernia have different rates of antenatal detection, and the potential benefits of being born in the right place will vary depending on the condition and the nature of the defect itself. However, they serve as exemplar congenital anomalies to explore the impact of birth location on short-term outcomes.

This review aims to understand the impact of delivery location on short term neonatal outcomes for infants born with congenital diaphragmatic hernia (CDH), gastroschisis and tracheoesophageal fistula or oesophageal atresia (TOF/OA). We aim to map the latest evidence to inform future research and influence neonatal service design.

METHOD:

Search methods for identification of studies

This systematic review was prospectively registered in PROSPERO (Registration number: CRD42022329090) and conducted in accordance with PRISMA (4,5). We searched the following databases: MEDLINE, CINAHL, Web of Science and SCOPUS. Supplementary File 1 includes example search strategy.

Selection criteria

Studies were eligible for inclusion if they:

I. were published between 1stJanuary2000 and 15thJanuary2023 in any language,
II. were conducted in a high-income country as defined by the World Bank (6)
III. included a study population of infants diagnosed with one of CDH, gastroschisis and TOF/OA
IV. provided data on birth location across at least two groups and,
V. reported at least one outcome of interest

Studies were excluded if they did not report delivery location, did not provide quantitative data or did not allow for comparisons. We also searched the bibliography of included studies, grey literature, and contacted experts for relevant papers.

Outcomes of interest

Outcomes of interest were mortality, length of stay, time to full enteral feeds, time to first feed, sepsis, presence of morbidity, duration of parenteral nutrition, presence of liver disease, growth and breastfeeding rates(as a proxy measure for maternal-separation). Outcomes were decided upon reviewing the literature, including core outcome set on gastroschisis and core outcome set in neonatology (7,8).

Selection of studies

https://mc.manuscriptcentral.com/bmjpo

Confidential: For Review Only

Three reviewers (BNM, LC, GS) screened title and abstracts using CADIMA software for records management, deduplicate, screening and data extraction (9). Conflicts were resolved by team discussions. Data extraction was carried out using a predetermined template to include baseline characteristics and outcome data. Two reviewers (BNM and GS) conducted data extraction independently with conflicts resolved through discussions. Only data related to CDH, gastroschisis, or TOF/OA were extracted from studies involving infants with other surgical conditions.

Risk of bias and quality of evidence assessment

The Newcastle Ottawa Scale (NOS) assessed quality and risk of bias of included studies (10), using nine points across three domains: (I) participants selection; (II) comparability and (III) detection of exposure/outcome. Depending on the scoring, studies were rated 'good', 'fair', or 'poor' quality using standard guidance. The NOS assessment was independently done by two reviewers (BNM and GS) and discrepancies resolved by discussion. Studies that included more than one condition of interest but used the same methodology were assessed once. Studies that include the same population or potentially overlapping populations were independently assessed.

Data extraction and analysis

Data extraction followed a predefined collection form. We present findings grouped by condition. For each outcome we present, when available, the absolute rates as well as unadjusted and adjusted results in cases and control, and results of statistical significance tests. We use vote-counting based on the direction of effect methodology, comparing the number of positive studies – that showed benefit – to the number of negative studies – showing harm. We report direction of effect by outcome and study quality, and we also present the overall effect of birth location (11). The author's conclusion was also considered in the overall effect.

Patient and public involvement statement

No patients or members of the public were involved in this systematic review. As this was a review of available literature, no ethics approval was necessary.

RESULTS:

Description of studies

Of 1398 records retrieved, 909 remained after deduplication and were screened on title and abstract. 813 were excluded on title and abstract and ninety-five full texts were retrieved and assessed for eligibility. Nineteen studies met our inclusion criteria and were included in the review. Reasons for exclusion included the study setting, the lack of a control and exposure group or no comparison between groups provided in the results. Grey literature and bibliography searches did not yield any further references. Figure 1 shows the PRISMA flow diagram for the records found and screened.

Study characteristics

Studies were either conducted in a single country e.g., Australia(12–14), Canada (15–17), France (18), Germany (19), Japan (20), USA (21–28), or at an international level across multiple countries (29,30). One study included data on all three congenital malformations of interest for the review (12), whilst the rest focused on only one of them. Study size ranged from 50 to 4663 babies, and we expect overlap in the populations included across the studies in view of the time-period and settings included. Case-mix adjustments were carried out in eight studies (12,13,15,16,22,27,29,31). The main characteristics of included studies are summarised in Table 1.

Mortality was most reported; however, the definition of mortality was inconsistent across studies. Other outcomes reported included length of hospital stay, postnatal age at first feed, postnatal age at full enteral feeding, presence of comorbidities and duration of total parenteral nutrition. Only mortality was reported for all conditions, and some outcomes were not reported for certain conditions.

Quality assessment

We assessed study quality using the NOS (10). A composite score depending on the score across the three domains was assigned to each study, as described previously. Study quality assessment is summarised on Table 2. All studies performed well in the selection and outcome domains of the tool, however performance in the comparability domain was variable. Ten studies were rated as 'good' quality, and nine as 'poor' quality.

https://mc.manuscriptcentral.com/bmjpo

Protocol modifications

High degree of heterogeneity precluded a meta-analysis. Instead, we present a narrative synthesis with vote-counting on the direction of effect (11).

Vote counting for measure of effect

We present the direction of effect for each study for the outcomes reported. In addition, we report the overall direction of effect of birth in surgical centre for CDH, gastroschisis and TOF/OA. Figure 2 displays vote-counting on overall direction of effect. Table 3 summarises direction of effect per outcome and the quality rating per study.

Confidential: For Review Only

Author/Year / Setting	Population	Study Sample	Comparison	Outcomes	Results – absolute numbers	Results – analysis presented	Author's conclusion on the effect of birth location on outcomes ?**
Al-Shanafey et al., 2002 Halifax, Canada (17)	Infants born with CDH born in or referred to regional unit between 1973-1999	81 infants total : 41 transferred to surgical centre (outborn)	Born in specialist surgical centres vs born outside and referred	Mortality	No transfer:<br>9/40 died<br>Transfer:<br>7/41 died	Non-significant difference between groups P=0.54	No difference
Algert et al., 2008, New South Wales, Australia(12)	Infants born alive with CDH managed in a single regional centre between 2001-2003	57 infants total: 22 transferred to surgical centre (outborn)	'Co-located hospital' – not requiring neonatal emergency transport vs with 'Other hospital' – requiring transport	1-year Mortality	No transfer:<br>19/35 died<br>Transfer:<br>4/22 died	Significant difference between groups P<0.01	Favour birth in surgical centre/ regionalisation of services
Aly et al., 2010, USA (21)	All infants with CDH and respiratory distress requiring mechanical ventilation admitted to selected hospitals during 1997-2004 ς	2140 infants total: 1020 transferred to surgical centre (outborn)	Group 1 did not undergo transport vs group 2 transported from the birth hospital to the surgical facility	Mortality after surgery	No transfer:<br>143/1120 died<br>Transfer:<br>165/1020 died	aOR: 1.46 (1.02-2.05) P=0.02	Favour birth in surgical centre/ regionalisation of services
Boloker et al., 2002, New York, USA (22)	Infants born with CDH and admitted to a single surgical centre during 1992-2000 ς	120 infants total: 53 transferred to surgical centre (outborn)	Inborn vs outborn transfers	Mortality	No transfer: 23/67 died<br>Transfer:<br>6/53 died		No difference
Carmichael et al., 2020. California, USA (23)	Infants born with CDH during 2006-2011 in California ς	577 infants total: 357 transferred to surgical centre (outborn)	Transfer from birth hospital for repair vs no transfer from birth hospital	Mortality to infancy	No transfer: 103/220 died<br>Transfer:<br>77/357 died	Unadjusted:<br>HR: 0.4(0.3-0.5)<br>Adjusted:<br>aHR: 0.3 (0.2-0.5)	Favour birth in surgical centre/ regionalisation of services
Gallot et al., 2007, Centrar-Eastern France (18)	Infants born with CDH during 1986-2003	387 infants total: 211 transferred (outborn)	Delivery in tertiary hospital vs peripheral hospital and transfer	Mortality	No transfer: 78/176 died<br>Transfer:<br>74/211 died	Group difference:<br>P=0.06	Favour birth in surgical centre/ regionalisation of services
Nagata et al., 2013, Japan (20)	Infants born with CDH during 2006-2010 ς	614 infants total: 165 transferred to surgical centre (outborn)	Inborn vs outborn	Mortality	No transfer: 129/449 died<br>Transfer:<br>22/165 died	Group difference:<br>P<0.001	N/A
Nasr and Langer, 2011, Canada (15)	Infants born with CDH during 2005-2008 ς	140 infants total: 65 transferred (outborn)	Inborn: born in the same building as NICU vs outborn requiring an ambulance transfer	Mortality	No transfer: 17/75 died<br>Transfer:<br>21/65	Group difference:<br>P=0.30<br>aOR 2.8, P=0.04	Favour birth in surgical centre/ regionalisation of services
Putnam et al., 2016, Congenital Diaphragmatic Hernia Study Group: Australia, Canada, Chile, Germany, Italy, Japan, Malaysia, Poland, Russia, Scotland, Sweden, The Netherlands, USA (29)	Infants born with CDH during 2007-2014 ς	3665 infants: number of transferred not available	Inborn vs outborn	Pulmonary morbidity<br><br><br>Neurologic morbidity<br><br><br>Gastrointestinal morbidity	327/660 transferred and had pulmonary morbidity<br><br>228/446 transferred and had neurologic morbidity<br><br>787/1348 transferred and had gastrointestinal morbidity	OR 1.25 (0.86-1.84), P=0.247<br><br><br>OR 1.04 (0.37-1.47), P=0.848	No difference

Confidential: For Review Only

						OR 1.64 (1.11-2.42), P=0.013	
Sola et al., 2010, USA (24)	Infants with CDH during 1997-2006 ς	2774 infants total: 891 transferred (outborn)	Hospital transfer vs birth	Mortality	No transfer: 333/1680 died<br>Transfer:<br>212/891 died	Group difference: P=0.003	No difference
Stopenski et al., 2022, Congenital Diaphragmatic Hernia Study Group: Australia, Canada, Chile, Germany, Italy, Japan, Malaysia, Poland, Russia, Scotland, Sweden, The Netherlands, USA (30)	Infants with CDH during 2007-2019 with prenatal diagnosis ς	4195 infants total: 1108 transferred (outborn)	Inborn- delivered and treated at CDHSG hospitals. Outborn - delivered in a separate institution and required transfer to a definitive care center.	Mortality	No transfer: 2082/3087 died<br>Transfer:<br>733/1108 died	Group difference: P=0.44<br><br>OR: 1.07 (0.015-1.25) P=0.391	No difference

Table 1a: Summary table of included studies on CDH (See Supplementary Materials for complete table).

*not all studies provided statistical analysis i.e., ORs, some studies only described the number of events

**not all authors provided conclusions on favoured birth location

RR- Risk Ration, aRR- adjusted Risk Ratio, OR – Odds Ratio ,aOR – adjusted Odds Ratio, P- p-value, HR hazard ratio, aHR – adjusted Hazard Ratio

† Length of stay given as median (IQR)

ς Potentially overlapping cohort: multicentre or population-based studies may include duplicates infants

Confidential: For Review Only

**Author/Year/ Setting**	**Population**	**Study Sample**	**Comparison**	**Outcomes**	**Results – absolute numbers**	**Results – analysis presented**	**Author's conclusion on the effect of birth location on outcomes ?****
Algert et al., 2008, New South Wales, Australia (12)	Infants born with gastroschisis during 2001-2003	62 infants: 11 transferred to a surgical centre	Co-located hospital – not requiring neonatal emergency transport compared with Other hospital – requiring transport	1-year Mortality	Transfer:<br>0/11 died<br>No transfer:<br>7/51 died	Non-significant difference	No difference
Dalton et al., 2017, USA (25)	Infants born with gastroschisis during 2010-2015 ς	79 infants total: 18 transferred to surgical centre (outborn)	Inborn vs outborn	Length of stay†<br><br>Days to first feed<br><br>Days to full feed	Transfer:<br>40+/- 26 days<br>No transfer:<br>30+/- 15 days<br><br>Transfer:<br>15+/-8.4 days<br>No transfer:<br>13+/-8.5 days<br><br>Transfer:<br>36+/- 23 days<br>No transfer:<br>16 +/- 54 days	Group difference:<br>P= 0.03<br><br>Group difference:<br>P=0.17<br><br>Group difference:<br>P=0.07	Favour birth in surgical centre/ regionalisation of services

				Total Parenteral Nutrition days	Transfer:<br>32+/- 23 days<br>No transfer:<br>23+/- 12 days	Group difference:<br>P= 0.03	
Hong et al., 2019, USA (26)	Infants >1500g born with gastroschisis during 2009-2015 ς	4663 infants total: 1134 transferred (outborn)	Inborn – birth in surgical centre vs outborn – delivery outside of centre	Mortality<br><br>Length of stay†	Transfer:<br>23/1134 died<br>No transfer:<br>67/3529 died<br><br>Transfer:<br>38 (27,64) days<br>No transfer:<br>35 (26,55) days	aOR: 1.02 (0.60-1.72) P>0.05<br><br>Poisson regression:<br>0.10 (0.04,0.16)<br>P<0.05	No difference
Kandasamy et al., 2009, North Queensland, australia (13)	Infants born with gastroschisis during 1988-2007 ς	50 infants total: 10 transferred (outborn)	Inborn vs outborn	Mortality<br><br>Length of stay	No numerical data available – No difference between groups found		No difference
Kitchanan et al., 2000, North Queensland, australia (14)	Infants born with gastroschisis during 1988-1997ς	21 infants (inborn): 4 transferred (outborn)	Inborn – delivered in regional tertiary unit vs outborn – delivered in peripheral hospitals	Days to first feed<br><br>Days to full enteral feeding<br><br>Total Parenteral Nutrition days	Transfer:<br>25 days<br>No transfer:<br>9 days<br><br>Transfer:<br>19 days<br>No transfer:<br>16 days<br><br>Transfer:<br>42 days<br>No transfer:<br>14 days	Group difference:<br>P=0.012<br><br>Group difference:<br>P= 0.02<br><br>Group difference:<br>P=0.012	No difference
Nasr and Langer, 2012, Canada (16)	Infants born with gastroschisis during 2005-2008	395 infants: 158 transferred	Inborn: born in the same building as NICU vs outborn requiring an ambulance transfer	Mortality<br><br>Length of stay†<br><br>Days to first feed<br><br>Total Parental Nutrition days	Transfer:<br>4/158 died<br>No transfer:<br>11/237 died<br><br>Transfer:<br>49 (27,62) days<br>No transfer:<br>48 (21,54) days<br><br>Transfer:<br>12 (6,14) days<br>No transfer:<br>12 (7,15) days	Group difference:<br>P=0.10<br><br>Group difference:<br>P=0.80<br><br>Group difference:<br>P=0.85<br><br>Group difference:<br>P= 0.19	No difference

Confidential: For Review Only

				Complications	Transfer:<br>42 (22,40) days<br>No transfer:<br>36 (17,40) days<br><br>Transfer:<br>4/158<br>No transfer:<br>11/237	Group difference:<br>P=0.10<br>OR 1.60 (1.09-2.7),<br>P=0.05	
Savoie et al., 2014, USA (27)	Infants with gastroschisis during 2008-2013	524 infants total: 239 transferred outborn	Inborn- delivery in hospital where surgical repair occurred vs outborn- required transfer to surgical centre for repair	30-day Mortality<br><br>Length of stay†<br><br>Days to first enteral feed<br><br>Days to full enteral feeding<br><br>Total Parenteral Nutrition days	Transfer:<br>4/239 died<br>No transfer:<br>4/283 died<br><br>Transfer:<br>42 (28,76) days<br>No transfer:<br>34 (24,63) days<br><br>Transfer:<br>18 (13,26) days<br>No transfer:<br>16 (11,22) days<br><br>Transfer:<br>31 (23,57) days<br>No transfer:<br>27 (20,43) days<br><br>Transfer:<br>29 (20,54) days<br>No transfer:<br>27 (17,445) days	Group difference:<br>P=0.81<br><br>Group difference:<br>P=0.002<br><br>Group difference:<br>P=0.004<br><br>Group difference:<br>P=0.0008<br><br>Group difference:<br>P=0.06	Favour birth in surgical centre/ regionalisation of services

Table 1b: Summary table of included studies on gastroschisis (See Supplementary Materials for complete table).
*not all studies provided statistical analysis i.e., ORs, some studies only described the number of events
**not all authors provided conclusions on favoured birth location
RR- Risk Ratio, aRR- adjusted Risk Ratio, OR – Odds Ratio ,aOR – adjusted Odds Ratio, P- p-value, HR hazard ratio, aHR – adjusted Hazard Ratio
† Length of stay given as median (IQR)
ς Potentially overlapping cohort: multicentre or population-based studies may include duplicates infants

Confidential: For Review Only

Author/Year/ Setting	Population	Study Sample	Comparison	Outcomes	Results – absolute numbers	Results – analysis presented	Author's conclusion on the effect of birth location on outcomes ?**
Algert et al., 2008, New South Wales, Australia (12)	Infants with oesophageal atresia during 2001-2003	42 infants: 25 transferred	Co-located hospital – not requiring neonatal emergency transport compared with 'Other hospital' – requiring transport	1-year Mortality	Transfer: 1/25 died<br>No transfer: 3/17 died	Non-significant difference between groups	No differences
Schlee et al., 2022, Germany (19)	Infants with oesophageal atresia during 2009-2013	57 infants total: 35 transferred (outborn)	Inborn- born in institution vs outborn – referred postnatally and transported for surgery	1-year mortality<br><br>Length of stay†<br><br>Days to full enteral feeding	Transfer: 0/35 died<br>No transfer: 2/17 died<br><br>Transfer: 17 (13,36) days<br>No transfer: 92 (18,158) days<br><br>Transfer: 8 (6,11) days<br>No transfer: 12 (7,18) days	Group Difference: P= 0.103<br><br>Group difference: P= 0.009<br><br>Group difference: P=0.137	No difference
Wang et al., 2014, USA (28)	Infants with oesophageal atresia/trachoesophageal fistula during 1997-2009	4168 infants total: 1169 transferred (outborn)	Divided by admission source: Birth vs Hospital transfer	Mortality	Transfer: 99/1169 died<br>No transfer: 186/1850 died	Group difference: P=0.399	No difference

Table 1c: Summary table of included studies on TOF/OA (See Supplementary Materials for complete table).

*not all studies provided statistical analysis i.e., ORs, some studies only described the number of events

**not all authors provided conclusions on favoured birth location

RR- Risk Ration, aRR- adjusted Risk Ratio, OR – Odds Ratio ,aOR – adjusted Odds Ratio, P- p-value, HR hazard ratio, aHR – adjusted Hazard Ratio

† Length of stay given as median (IQR)

ς Potentially overlapping cohort: multicentre or population-based studies may include duplicates infants

Confidential: For Review Only

	Selection				Comparability		Outcome			Overall rating §
**Author, year †**	1	2	3	4	5	6	7	8	9	
Al-Shanafey (2002) (17)	*	*	*	*			*	*	*	Poor
Algert (2008) (12)	*	*	*	*			*	*	*	Poor
Aly (2010) (21)	*	*	*	*	*	*	*	*	*	Good
Boloker (2002) (22)	*	*	*	*			*	*	*	Poor
Carmichael (2020) (23)	*	*	*	*	*	*	*	*	*	Good
Dalton (2017) (25)	*	*	*	*			*	*	*	Poor
Gallot (2007) (18)	*	*	*	*	*		*	*	*	Good
Hong (2019) (26)	*	*	*	*	*		*	*	*	Good
Kandasamy (2009) (13)	*	*	*	*			*	*	*	Poor
Kitchanan (2000) (14)	*	*	*	*			*	*	*	Poor
Nagata (2013) (20)	*	*	*	*			*	*	*	Poor
Nasr (2011) (15)	*	*	*	*	*	*	*	*	*	Good
Nasr (2012) (16)	*	*	*	*	*	*	*	*	*	Good
Putnam (2016) (29)	*	*	*	*	*		*	*	*	Good
Savoie (2014) (27)	*	*	*	*	*		*	*	*	Good
Schlee (2022) (19)	*	*	*	*			*	*	*	Poor
Sola (2010) (24)	*	*	*	*	*		*	*	*	Good
Stopenski (2022) (30)	*	*	*	*	*	*	*	*	*	Good
Wang (2014) (28)	*	*	*	*			*	*	*	Poor

Table 2: Quality Assessment – Newcastle Ottawa Quality Assessment Scale for Cohort Studies

†Cohort studies: 1. representativeness of the exposed cohort, 2. selection of the non-exposed cohort, 3. ascertainment of exposure, 4. demonstration that the outcome of interest was not present at start of the study, 5. comparability of cohorts based on the design or analysis most important factor, 6. comparability of cohorts based on the design or analysis second important factor, 7. assessment of outcome, 8. was follow-up long enough for outcomes to occur and 9. adequacy of follow-up of cohort.

§Good quality rating for those with 3 or 4 stars in selection domain AND 1 or 2 stars in comparability domain AND 2 or 3 stars in outcome/exposure domain. Fair quality for 2 stars in selection domain AND 1 or 2 stars in comparability domain AND 2 or 3 stars in outcome/exposure domain. Poor quality for 0 or 1 star in selection domain OR 0 stars in comparability domain OR 0 or 1 stars in outcome/exposure domain

Confidential: For Review Only

Author/Year	Mortality	Length of Stay	Age First Enteral feed	Days to full enteral Feeding	PN Duration	Morbidity	Overall direction of effect (D) of birth in surgical centre
**CONGENITAL DIAPHRAGMATIC HERNIA**							
Al-Shanafey et al., 2002 (17)	◀▶						◀▶
Algert et al., 2008 (12)	▼						▼
Aly et al., 2010 (21)	▼ §						▼ §
Boloker et al., 2002 (22)	◀▶						◀▶
Carmichael et al., 2020 (23)	▼§						▼ §
Gallot et al., 2007 (18)	▼						▼
Nagata et al., 2013 (20)	◀▶						◀▶
Nasr and Langer, 2011 (15)	▼§						▼§
Putnam et al., 2016 (29)						◀▶§	◀▶§
Sola et al., 2010 (24)	◀▶						◀▶
Stopenski et al., 2022 (30)	◀▶§						◀▶§
**GASTROSCHISIS**							
Algert et al., 2008 (12)	◀▶						◀▶
Dalton et al., 2017 (25)		▼	▼	▼	▼		▼
Hong et al., 2019 (26)	◀▶§	◀▶§					◀▶§
Kandasamy et al., 2009 (13)	◀▶	◀▶					◀▶
Kitchanan et al., 2000 (14)			▼	▼	▼		◀▶§
Nasr and Langer, 2012 (16)	◀▶§	◀▶§	◀▶§	◀▶§	◀▶§		◀▶§
Savoie et al., (27)	◀▶§	▼§	▼§	▼§	▼§		▼§
**OESOPHAGEAL ATRESIA / TRACHEOESOPHAGEAL FISTULA**							
Algert et al., 2008 (12)	◀▶						◀▶
Schlee et al., 2022 (19)	◀▶	▼		◀▶			◀▶
Wang et al., 2014 (28)	◀▶						◀▶

Table 3: Summary of direction of effect of birth in a surgical unit

Outcomes of interest and the overall direction of effect of birth in a surgical centre compared to birth outside a surgical centre (D) showed for each study included.
Arrows denote harm (▲), benefit (▼) or conflicting evidence (◀▶).
§ denotes studies carried out case-mix adjustments
Red colour – 'Poor' quality, Yellow colour – 'Fair' quality, Green colour- 'Good' quality.

Confidential: For Review Only

Congenital diaphragmatic hernia

Eleven observational studies included babies with CDH, two were prospective whilst nine were retrospective studies (12,15,17,18,20–24,29,30). Three used single centre data to compare two populations – infants born within their unit compared to infants referred from other hospital and transferred in. The other eight studies explored data across multiple centres through patient databases and used the birth location and subsequent transfers for surgical management to define two groups. Mortality was reported in ten studies; however, the definition of mortality varied – from death during admission to death in infancy. Two studies included data from multiple countries, members of the Congenital Diaphragmatic Hernia Study Group (CDHSG) (29,30). There is potential overlap in populations across studies using patient databases, however we were not able to distinguish the cohorts. The two studies using data from the CDHSG include the same population, however they report different outcomes (mortality versus morbidity) and as such both studies have been included.

Five studies with exploring mortality favoured delivery in a surgical centre for infants with CDH– three of these rated as 'good' quality studies (Figure 2). The other five studies reported no difference in mortality. No difference in morbidity rate was reported in an international study from the CDHSG (29).

We are not able to report other outcomes such as length of stay, postnatal age at first feed, and duration of parenteral nutrition due to reporting limitation as these were unreported in the included studies.

Five population-level studies carried out case-mix adjustments. Three of these studies, concluded that birth in surgical centre was favourable for this population, whilst two studies using international data did not find a difference in mortality after case-mix adjustments. We also found differences in the population included in the studies, some studies excluded infants with other congenital anomalies whilst other studies had no exclusion criteria – this brings selection bias and would influence the mortality rates reported. In addition, the rate of antenatal diagnosis and termination of pregnancy for congenital anomalies varies across study setting and period, which would impact the birth location and the survival rate (32,33). It is important to highlight that some authors recognised that the reported results may be skewed by 'hidden mortality', as infants may have died at delivery or prior to surgery or reaching the surgical centre and that would have impacted on the population included in their study.

Gastroschisis

Seven studies explored the impact of delivery site on the outcomes for neonates diagnosed with gastroschisis (12–14,16,25–27). All studies were single country. Three studies were rated 'good' quality and four 'poor' quality. Three multicentre studies used population level databases (16,26,27). Inclusion and exclusion criteria varied across the studies, notably one study excluded complicated gastroschisis (25), whilst others did not describe whether the population study included simple or complex gastroschisis, limiting comparison of the findings.

There may be overlap across USA-based cohorts, but we were unable to distinguish them. Five studies reported on mortality and all found no differences between groups (12,13,16,26,27). Median length of stay was explored in five studies. Two of these found transfers to a surgical centre was associated longer stay, these were rated as 'poor' and 'good' quality studies (25,27). The other three studies found no difference in length of stay (13,16,26). Four studies reported on duration of total parenteral nutrition, and one study, rated as 'poor' quality, found that those infants born outside a surgical centre had a longer duration whilst the others found no significant difference (14,16,25,27). One study adjusted for clustering within hospital and found no difference in mortality or length of stay (26). Two studies adjusted for case-mix of unspecified characteristics and one concluded that birth in surgical centre led to favourable outcomes, particularly length of stay (29).

Oesophageal atresia and tracheoesophageal atresia

Three included studies explored the impact of birth location and outcomes for babies diagnosed with oesophageal atresia or tracheoesophageal atresia (12,19,28). All studies used retrospective data. Algert et al., and Schlee et al., included data from a single surgical centre in Australia and Germany respectively, whilst Wang et al., used population-level data to study the population across the USA, findings showed no difference on mortality by location of birth but lower mortality in specialised children's hospitals after excluding for immediate transfers . All studies were rated 'poor' quality. No study adjusted for case-mix in their analysis. No

https://mc.manuscriptcentral.com/bmjpo
Confidential: For Review Only

differences in outcomes of interest – mortality, length of stay or days to full enteral feeding – were found across the studies included.

DISCUSSION:

This systematic review identified nineteen studies exploring the impact of birth location on short-term outcomes of infants born with gastroschisis, congenital diaphragmatic hernia or oesophageal atresia and tracheoesophageal atresia. All studies were observational cohort studies using data from high income countries to explore the impact of birth location on short-term outcomes. Although we aimed to report on different short-term outcomes, mortality was the only outcome that we could report for all three condition due to the data available. Study quality and risk of bias was assessed using the Newcastle-Ottawa Scale. Case-mix adjustment, not included within the quality rating, was not carried out in all studies. High study heterogeneity precluded a meta-analysis. We present a narrative synthesis with vote counting on the direction of effect. The overall direction of effect suggests limited evidence that birth in a non-surgical centre may be associated with worse outcomes particularly for increased mortality for CDH and prolonged length of stay in gastroschisis, with conflicting evidence for other outcomes and TOF/OA (Figure 2). No studies explored the impact of birth location on maternal-baby separation, and impact on breastfeeding, skin to skin or family integrated care, despite the positive impact of family integrated care on neonatal outcomes (34).

We found high heterogeneity across all included studies, and findings need to be interpreted with caution. Across all three conditions, fetal and neonatal variables e.g. associated congenital anomalies, comorbidities, surgical techniques, and surgical complications may impact on outcomes and were either not reported or not adjusted for. In addition, the time of diagnosis, the nature of the defect whether simple or complex and the size of the defect may impact both the location of birth as well as the short-term outcomes. The included population also varied, for example studies exploring CDH, may have included complex babies with severe cardiac anomalies or genetic conditions. Complex babies are more likely to be delivered in centres with co-located services, increasing the risk of bias in our findings (12). In addition, outcome reporting was inconsistent, limiting our ability to draw conclusions - for example some studies reported mortality to discharge home whilst other reported mortality to one year of age (35). We included studies across a 20-year period, during which there have been significant improvements in fetal medicine, neonatology and surgery. This is reflected in the changes in survival rates during this time, for example CDH mortality rates have decreased by 2.4% annually since 2001 (36–39). This introduces a time-period bias when comparing the results.

Overall, we found conflicting evidence regarding the importance of location of birth for congenital surgical anomalies. There is limited evidence to suggest that birth outside a surgical centre may be associated with worse neonatal outcomes, particularly for infants with CDH which carries a high mortality rate in the first few days after birth. Similarly, requiring transfer for surgical repair of a defect such as gastroschisis may lead to delay in establishing enteral feeding and prolonged hospital stays, as found in some of the studies included. However, it is important to recognise that delivery of infants with congenital surgical condition in centres with paediatric surgery services may also have disadvantages for families. For example, evaluation of the centralisation of congenital cardiac services in England has identified trade-offs between improved clinical outcomes and family experience – with reorganisation of care potentially widening inequalities and impacting access, with increased travel times and costs (40).

Strengths of this systematic review include the comprehensive search criteria to yield international literature from high-income countries, with no limitations on language of publication and prospective registration. In addition to published literature, we also other sources of literature and consulted experts for additional studies. We present all outcomes reported in the included studies to reduce selective outcome reporting bias. Limitations of this review includes the degree of bias discussed as well as not being able to carry out a meta-analysis of the studies included. The studies included, particularly for gastroschisis and TOF/OA, report on a population from nearly ten years ago and does reflect contemporary care which has since advanced. For gastroschisis, for examples, bedside sutureless umbilical closure without intubation has been shown to be a feasible initial management that reduces time to gastroschisis closure (41). Similarly, minimally invasive surgery for the repair of long gap oesophageal atresia may also bring about improvements in outcomes for TOF/OA. For CDH, fetoscopic endoluminal tracheal occlusion (FETO) may be used to treat severe cases of CDH in utero (42,43). This introduces a time bias as well as a selection bias to our results and the findings may not

Confidential: For Review Only

be generalisable. In addition, the lack of granular details on the severity or complexity of the condition and antenatal and postnatal interventions can make it difficult to conduct valid comparisons. Most studies report on transfer or outborn status (commonly requiring an ambulance transfer after delivery), as the birth location exposure. However, these terms are not consistently defined and may mean different things depending on setting and configuration of services. The rate of antenatal diagnosis was poorly reported throughout the studies. As a result, we are not able to separate those infants that were antenatally diagnosed and born in a surgical centre, versus those that had an antenatal diagnosis, but birth occurred outside a surgical centre (which may be planned for small defects, or unplanned), versus those that were not antenatally diagnosed. Case-mix adjustments is key to making valid and informative comparisons, due to the spectrum of disease complexity across these three conditions, for example simple versus complex gastroschisis or large defects to smaller CDH, for example.

Future research is needed to yield concrete evidence on the impact of birth outside a surgical centre on infants with congenital surgical conditions. This should include studies across different spectrums of disease severity and explore delivery in different location from high volume centres with maternity on-site, to high volume without maternity services as well low volume centres and births outside of a surgical centre. Using established routine or registry-based datasets may be the most efficient way of capturing whole-population data to explore this relationship. Analysis should adjust for case-mix, including maternal and infant characteristics. Additional outcomes, for example maternal-baby separation, as well as long-term outcomes should also be explored. Qualitative work exploring the experience of parents and carers of infants that undergo transfers postnatally will complement quantitative work and provide strong evidence to shape future design of neonatal health services.

In conclusion, we found limited evidence suggesting that birth in a non-surgical centre may contribute to prolonged length of stay and increase mortality. However, the quality of evidence was insufficient to make concrete conclusions and further research is needed.

FUNDING STATEMENT

This research is support by the National Institute for Health Research grant ACF-2020-21-011 awarded to BNM. CG was supported by the United Kingdom Medical Research Council through a Transitional Support Fellowship (MR/V036866/1). CB is supported through a UK NIHR Advanced Fellowship personal award (NIHR300617). Imperial College London Open Access Fund supported the publication and dissemination of this work.

COMPETING INTERESTS

None declared.

CONTRIBUTIONS

BNM, DH, CG and CB conceived and designed the study. BNM designed and carried out the search strategy. BNM, GS and LC reviewed search results, screened title/abstracts and full texts and extracted data from included studies. BNM and GS interpreted the results. BNM wrote the first draft and all authors contributed to the final manuscript. All authors have approved the final manuscript as submitted.

Confidential: For Review Only

References:

1. Kenny S. Getting it Right First Time: Paediatric Surgery GIRFT Programme National Specialty Report. [Internet]. NHS England and NHS Improvementt; 2021 Feb [cited 2022 Apr 16]. Available from: https://www.gettingitrightfirsttime.co.uk/wp-content/uploads/2021/09/PaediatricSurgeryReport-Sept21w.pdf

2. BAPM. National Care Principles for the Management of Congenital Diagphragmatic Hernia (2018) [Internet]. British Association of Perinatal Medicine. [cited 2023 Jan 14]. Available from: https://www.bapm.org/resources/22-national-care-principles-for-the-management-of-congenital-diagphragmatic-hernia-2018

3. Snoek KG, Reiss IKM, Greenough A, Capolupo I, Urlesberger B, Wessel L, et al. Standardized Postnatal Management of Infants with Congenital Diaphragmatic Hernia in Europe: The CDH EURO Consortium Consensus - 2015 Update. Neonatology. 2016;110(1):66–74.

4. Moher D, Liberati A, Tetzlaff J, Altman DG, PRISMA Group. Preferred reporting items for systematic reviews and meta-analyses: the PRISMA statement. BMJ. 2009 Jul 21;339:b2535.

5. Registration of systematic reviews in PROSPERO: 30,000 records and counting | Systematic Reviews | Full Text [Internet]. [cited 2023 Jan 10]. Available from: https://systematicreviewsjournal.biomedcentral.com/articles/10.1186/s13643-018-0699-4

6. World Bank Country and Lending Groups – World Bank Data Help Desk [Internet]. [cited 2023 Mar 4]. Available from: https://datahelpdesk.worldbank.org/knowledgebase/articles/906519-world-bank-country-and-lending-groups

7. Webbe JWH, Duffy JMN, Afonso E, Al-Muzaffar I, Brunton G, Greenough A, et al. Core outcomes in neonatology: development of a core outcome set for neonatal research. Archives of Disease in Childhood - Fetal and Neonatal Edition. 2020 Jul 1;105(4):425–31.

8. Allin BSR, Hall NJ, Ross AR, Marven SS, Kurinczuk JJ, Knight M. Development of a gastroschisis core outcome set. Archives of Disease in Childhood - Fetal and Neonatal Edition. 2019 Jan 1;104(1):F76–82.

9. Kohl C, McIntosh EJ, Unger S, Haddaway NR, Kecke S, Schiemann J, et al. Online tools supporting the conduct and reporting of systematic reviews and systematic maps: a case study on CADIMA and review of existing tools. Environmental Evidence. 2018 Feb 1;7(1):8.

10. Ottawa Hospital Research Institute [Internet]. [cited 2023 Jan 10]. Available from: https://www.ohri.ca/programs/clinical_epidemiology/oxford.asp

11. Higgins J, Tthomas J, Chandler J, Cumpston M, Li T, Page M, et al. Cochrane Handbook for Systematic Reviews of Interventions [Internet]. [cited 2023 Jan 10]. Available from: https://training.cochrane.org/handbook

12. Algert C, Bowen J, Hadfield R, Olive E, Morris J, Roberts C. Birth at hospitals with co-located paediatric units for infants with correctable birth defects. AUSTRALIAN & NEW ZEALAND JOURNAL OF OBSTETRICS & GYNAECOLOGY. 2008;48(3):273–9.

13. Kandasamy Y, Whitehall J, Gill A, Stalewski H. Surgical management of gastroschisis in North Queensland from 1988 to 2007. JOURNAL OF PAEDIATRICS AND CHILD HEALTH. 2010;46(1–2):40–4.

14. Kitchanan S, Patole S, Muller R, Whitehall J. Neonatal outcome of gastroschisis and exomphalos: A 10-year review. JOURNAL OF PAEDIATRICS AND CHILD HEALTH. 2000;36(5):428–30.

15. Nasr A, Langer J, Canadian Pediat Surg Network. Influence of location of delivery on outcome in neonates with congenital diaphragmatic hernia. JOURNAL OF PEDIATRIC SURGERY. 2011;46(5):814–6.

Confidential: For Review Only

16. Nasr A, Langer J, Canadian Paediat Surg Network. Influence of location of delivery on outcome in neonates with gastroschisis. JOURNAL OF PEDIATRIC SURGERY. 2012;47(11):2022–5.

17. Al-Shanafey S, Giacomantonio M, Henteleff H. Congenital diaphragmatic hernia: experience without extracoporeal membrane oxygenation. PEDIATRIC SURGERY INTERNATIONAL. 2002;18(1):28–31.

18. Gallot D, Boda C, Ughetto S, Perthus I, Robert-Gnansia E, Francannet C, et al. Prenatal detection and outcome of congenital diaphragmatic hernia: a French registry-based study. Ultrasound Obstet Gynecol. 2007 Mar;29(3):276–83.

19. Schlee D, Theilen T, Fiegel H, Hutter M, Rolle U. Outcome of esophageal atresia: inborn versus outborn patients. DISEASES OF THE ESOPHAGUS. 2022;35(8).

20. Nagata K, Usui N, Kanamori Y, Takahashi S, Hayakawa M, Okuyama H, et al. The current profile and outcome of congenital diaphragmatic hernia: A nationwide survey in Japan. JOURNAL OF PEDIATRIC SURGERY. 2013;48(4):738–44.

21. Aly H, Bianco-Batlles D, Mohamed MA, Hammad TA. Mortality in infants with congenital diaphragmatic hernia: a study of the United States National Database. J Perinatol. 2010 Aug;30(8):553–7.

22. Boloker J, Bateman D, Wung J, Stolar C. Congenital diaphragmatic hernia in 120 infants treated consecutively with permissive hypercapnea/spontaneous respiration/elective repair. JOURNAL OF PEDIATRIC SURGERY. 2002;37(3):357–65.

23. Carmichael S, Ma C, Lee H, Shaw G, Sylvester K, Hintz S. Survival of infants with congenital diaphragmatic hernia in California: impact of hospital, clinical, and sociodemographic factors. JOURNAL OF PERINATOLOGY. 2020;40(6):943–51.

24. Sola J, Bronson S, Cheung M, Ordonez B, Neville H, Koniaris L. Survival disparities in newborns with congenital diaphragmatic hernia: a national perspective. JOURNAL OF PEDIATRIC SURGERY. 2010;45(6):1336–42.

25. Dalton B, Gonzalez K, Reddy S, Hendrickson R, Iqbal C. Improved outcomes for inborn babies with uncomplicated gastroschisis. JOURNAL OF PEDIATRIC SURGERY. 2017;52(7):1132–4.

26. Hong C, Fullerton B, Han S, Morrow K, Edwards E, Soll R, et al. Impact of disease-specific volume and hospital transfer on outcomes in gastroschisis. JOURNAL OF PEDIATRIC SURGERY. 2019;54(1):65–9.

27. Savoie K, Huang E, Aziz S, Blakely M, Dassinger S, Dorale A, et al. Improving gastroschisis outcomes: Does birth place matter? JOURNAL OF PEDIATRIC SURGERY. 2014;49(12):1771–5.

28. Wang B, Tashiro J, Allan B, Sola J, Parikh P, Hogan A, et al. A nationwide analysis of clinical outcomes among newborns with esophageal atresia and tracheoesophageal fistulas in the United States. JOURNAL OF SURGICAL RESEARCH. 2014;190(2):604–12.

29. Putnam L, Harting M, Tsao K, Morini F, Yoder B, Luco M, et al. Congenital Diaphragmatic Hernia Defect Size and Infant Morbidity at Discharge. PEDIATRICS. 2016;138(5).

30. Stopenski S, Guner Y, Jolley J, Major C, Hatfield T, Ebanks A, et al. Inborn Versus Outborn Delivery in Neonates With Congenital Diaphragmatic Hernia. JOURNAL OF SURGICAL RESEARCH. 2022;270:245–51.

31. Murphy F, Mazlan T, Tarheen F, Corbally M, Puri P. Gastroschisis and exomphalos in Ireland 1998-2004. Does antenatal diagnosis impact on outcome? PEDIATRIC SURGERY INTERNATIONAL. 2007;23(11):1059–63.

32. Aldridge N, Pandya P, Rankin J, Miller N, Broughan J, Permalloo N, et al. Detection rates of a national fetal anomaly screening programme: A national cohort study. BJOG. 2023 Jan;130(1):51–8.

Confidential: For Review Only

33. Morris JK, Springett AL, Greenlees R, Loane M, Addor MC, Arriola L, et al. Trends in congenital anomalies in Europe from 1980 to 2012. PLOS ONE. 2018 Apr 5;13(4):e0194986.

34. O'Brien K, Robson K, Bracht M, Cruz M, Lui K, Alvaro R, et al. Effectiveness of Family Integrated Care in neonatal intensive care units on infant and parent outcomes: a multicentre, multinational, cluster-randomised controlled trial. Lancet Child Adolesc Health. 2018 Apr;2(4):245–54.

35. Webbe JWH, Ali S, Sakonidou S, Webbe T, Duffy JMN, Brunton G, et al. Inconsistent outcome reporting in large neonatal trials: a systematic review. Arch Dis Child Fetal Neonatal Ed. 2020 Jan;105(1):69–75.

36. Bhatt P, Poku FA, Umscheid J, Ayensu M, Parmar N, Vasudeva R, et al. Trends in prevalence and mortality of gastroschisis and omphalocele in the United States from 2010 to 2018. World J Pediatr. 2022 Jul 1;18(7):511–4.

37. Tan Tanny SP, Beck C, King SK, Hawley A, Brooks JA, McLeod E, et al. Survival Trends and Syndromic Esophageal Atresia. Pediatrics. 2021 May;147(5):e2020029884.

38. Politis MD, Bermejo-Sánchez E, Canfield MA, Contiero P, Cragan JD, Dastgiri S, et al. Prevalence and mortality in children with congenital diaphragmatic hernia: a multicountry study. Annals of Epidemiology. 2021 Apr 1;56:61-69.e3.

39. Glinianaia SV, Rankin J, Pierini A, Coi A, Santoro M, Tan J, et al. Ten-Year Survival of Children With Congenital Anomalies: A European Cohort Study. Pediatrics. 2022 Feb 11;149(3):e2021053793.

40. Safe_and_Sustainable_Review_of_Childrens_Congenital_Cardiac_Services_in_England_Pre_Consultation_Business_Case.pdf [Internet]. [cited 2023 Feb 1]. Available from: https://www.chfed.org.uk/wp-content/uploads/2012/06/Safe_and_Sustainable_Review_of_Childrens_Congenital_Cardiac_Services_in_England_Pre_Consultation_Business_Case.pdf

41. Pet GE, Stark RA, Meehan JJ, Javid PJ. Outcomes of bedside sutureless umbilical closure without endotracheal intubation for gastroschisis repair in surgical infants. Am J Surg. 2017 May;213(5):958–62.

42. Patkowski D. Thoracoscopic approach for oesophageal atresia: A real game changer? Journal of Pediatric Surgery. 2023 Feb 1;58(2):204–8.

43. Deprest JA, Nicolaides KH, Benachi A, Gratacos E, Ryan G, Persico N, et al. Randomized Trial of Fetal Surgery for Severe Left Diaphragmatic Hernia. New England Journal of Medicine. 2021 Jul 8;385(2):107–18.

Confidential: For Review Only

Figure 1: PRISMA flowchart

Figure 2: Harvest plot of overall direction-of-effect in included studies by overall quality score.
Height depicts overall quality judgement and colour depicts surgical conditions (blue = CDH; yellow = gastroschisis; orange=TOF/OA).
Arrows denote adverse effects (↑), beneficial effects (↓) or conflicting evidence (⇔) of birth in a surgical unit.

Confidential: For Review Only

Confidential: For Review Only

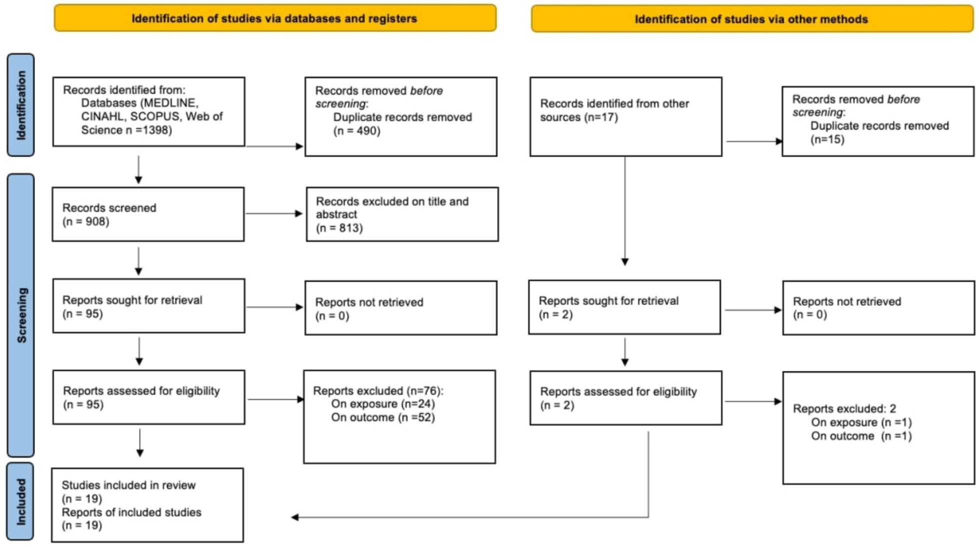

Figure 1: PRISMA flowchart

118x65mm (300 x 300 DPI)

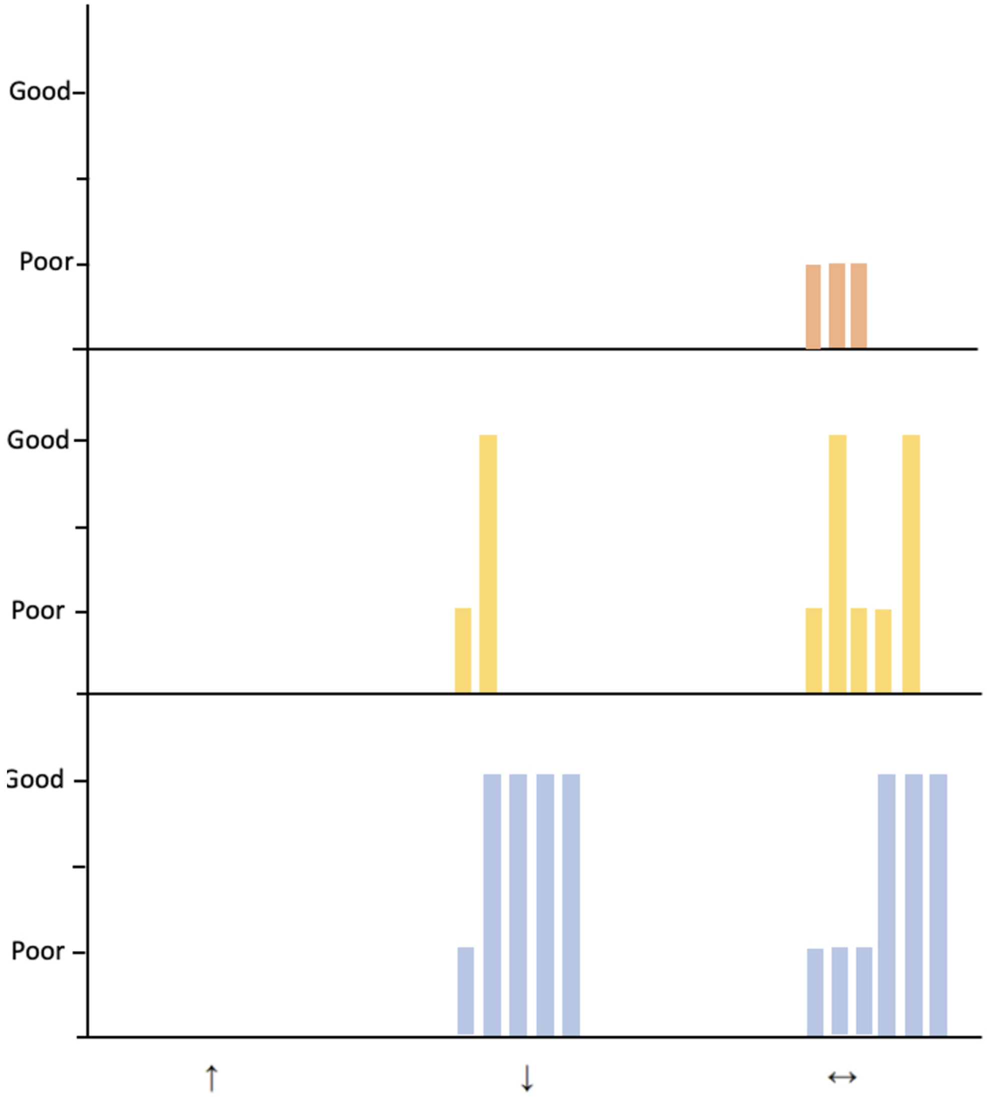

Figure 2: Harvest plot of overall direction-of-effect in included studies by overall quality score.
Height depicts overall quality judgement and colour depicts surgical conditions (blue = CDH; yellow = gastroschisis; orange=TOF/OA).
Arrows denote adverse effects (↑), beneficial effects (↓) or conflicting evidence (⇔) of birth in a surgical unit.

93x105mm (300 x 300 DPI)

CONGENITAL DIAPHRAGMATIC HERNIA											
**Author/Year**	**Setting**	**Population**	**Study Sample**	**Exclusions**	**Comparison**	**Outcomes**	**Analysis**	**Results – absolute numbers**	**Results – analysis presented**	**Adjustments for confounders**	**Author's conclusion on the effect of birth location on outcomes ?****
Al-Shanafey et al., 2002 (17)	Halifax, Canada	Infants born with CDH born in or referred to regional unit between 1973-1999	81 infants total : 41 transferred to surgical centre (outborn)	Diagnosis of lethal cardiac malformation Prenatal deaths and deaths immediately upon delivery	Born in specialist surgical centres vs born outside and referred	Mortality	Descriptive Chi-square test	No transfer: 9/40 died Transfer: 7/41 died	Non-significant difference between groups P=0.54	Nil	No difference
Algert et al., 2008 (12)	New South Wales, Australia	Infants born alive with CDH managed in a single regional centre between 2001-2003	57 infants total: 22 transferred to surgical centre (outborn)	Diagnosis made >28days after birth Antenatally diagnosable and lethal uncorrectable defects such as anencephaly, renal agenesis and trisomy 13 or 18	'Co-located hospital' – not requiring neonatal emergency transport vs with 'Other hospital' – requiring transport	1-year Mortality	Test of proportions	No transfer: 19/35 died Transfer: 4/22 died	Significant difference between groups P<0.01	Nil	Favour birth in surgical centre/ regionalisation of services
Aly et al., 2010 (21)	USA: Kids' Inpatient Database	All infants with CDH and respiratory distress requiring mechanical ventilation admitted to selected hospitals during 1997-2004 ς	2140 infants total: 1020 transferred to surgical centre (outborn)	Brain anomalies, abdominal wall defects, other congenital anomalies	Group 1 did not undergo transport vs group 2 transported from the birth hospital to the surgical facility	Mortality after surgery	Logistic regression modelling	No transfer: 143/1120 died Transfer: 165/1020 died	aOR: 1.46 (1.02-2.05) P=0.02	Gender, race, birth weight category, age at operation, comorbidities (pneumothorax, pulmonary hypertension, sepsis, necrotizing enterocolitis, birth asphyxia)	Favour birth in surgical centre/ regionalisation of services
Boloker et al., 2002 (22)	New York, USA	Infants born with CDH and admitted to a single surgical centre during 1992-2000 ς	120 infants total: 53 transferred to surgical centre (outborn)	Other congenital anomalies	Inborn vs outborn transfers	Mortality	Descriptive	No transfer: 23/67 died Transfer: 6/53 died		Nil	No difference
Carmichael et al., 2020 (23)	California, USA	Infants born with CDH during 2006-2011 in California ς	577 infants total: 357 transferred to surgical centre (outborn)	Infants with time of diagnosis unknown and infants that had fetal surgery	Transfer from birth hospital for repair vs no transfer from birth hospital	Mortality to infancy	Descriptive Hazard Ratio	No transfer: 103/220 died Transfer: 77/357 died	Unadjusted: HR: 0.4(0.3-0.5) Adjusted: aHR: 0.3 (0.2-0.5)	Demographics, Birthweight Gestational age Other birth defects	Favour birth in surgical centre/ regionalisation of services

Confidential: For Review Only

Gallot et al., 2007 (18)	Central-Eastern France	Infants born with CDH during 1986-2003	387 infants total: 211 transferred (outborn)	Nil	Delivery in tertiary hospital vs peripheral hospital and transfer	Mortality	Descriptive Chi-square test	No transfer: 78/176 died Transfer: 74/211 died	Group difference: P=0.06	Nil	Favour birth in surgical centre/ regionalisation of services
Nagata et al., 2013 (20)	Japan	Infants born with CDH during 2006-2010 ς	614 infants total: 165 transferred to surgical centre (outborn)	Diagnosis made >28days after birth	Inborn vs outborn	Mortality	Descriptive Chi-square test	No transfer: 129/449 died Transfer: 22/165 died	Group difference: P<0.001	Nil	N/A
Nasr and Langer, 2011 (15)	Canada	Infants born with CDH during 2005-2008 ς	140 infants total: 65 transferred (outborn)	Postnatal diagnosis	Inborn: born in the same building as NICU vs outborn requiring an ambulance transfer	Mortality	Descriptive Multivariate analysis	No transfer: 17/75 died Transfer: 21/65	Group difference: P=0.30 aOR 2.8, P=0.04	Adjustment for possible case-mix – unspecified variables	Favour birth in surgical centre/ regionalisation of services
Putnam et al., 2016 (29)	Congenital Diaphragmatic Hernia Study Group: Australia, Canada, Chile, Germany, Italy, Japan, Malaysia, Poland, Russia, Scotland, Sweden, The Netherlands, USA	Infants born with CDH during 2007-2014 ς	3665 infants: number of transferred not available	Nil	Inborn vs outborn	Pulmonary morbidity<br><br>Neurologic morbidity<br><br>Gastrointestinal morbidity	Descriptive Multivariate analysis	327/660 transferred and had pulmonary morbidity<br><br>228/446 transferred and had neurologic morbidity<br><br>787/1348 transferred and had gastrointestinal morbidity	OR 1.25 (0.86-1.84), P=0.247<br><br>OR 1.04 (0.37-1.47), P=0.848<br><br>OR 1.64 (1.11-2.42), P=0.013	Patient characteristics, including defect size	No difference
Sola et al., 2010 (24)	USA – Kids' Inpatient Database	Infants with CDH during 1997-2006 ς	2774 infants total: 891 transferred (outborn)	Admission >8 days of age	Hospital transfer vs birth	Mortality	Descriptive Chi-square test	No transfer: 333/1680 died Transfer: 212/891 died	Group difference: P=0.003	Nil	No difference
Stopenski et al., 2022 (30)	Congenital Diaphragmatic Hernia Study Group: Argentina, Canada, Chile, Egypt, England, Germany, Italy,	Infants with CDH during 2007-2019 with prenatal diagnosis ς	4195 infants total: 1108 transferred (outborn)	Postnatal diagnosis	Inborn-delivered and treated at CDHSG hospitals. Outborn - delivered in a separate	Mortality	Descriptive Logistic regression	No transfer: 2082/3087 died Transfer: 733/1108 died	Group difference: P=0.44<br><br>OR: 1.07 (0.015-1.25) P=0.391	Demographics Clinical severity	No difference

Confidential: For Review Only

	Japan, Malaysia, Netherlands, Poland, Qatar, Russia, Scotland, Spain, Sweden, USA				institution and required transfer to a definitive care center.						

Supplementary Table 1a: Summary of studies on CDH included.

*not all studies provided statistical analysis i.e., ORs, some studies only described the number of events

**not all authors provided conclusions on favoured birth location

RR- Risk Ration, aRR- adjusted Risk Ratio, OR – Odds Ratio ,aOR – adjusted Odds Ratio, P- p-value, HR hazard ratio, aHR – adjusted Hazard Ratio

† Length of stay given as median (IQR)

ς Potentially overlapping cohort: multicentre or population-based studies may include duplicates infants

**GASTROSCHISIS**											
**Author/Year**	**Setting**	**Population**	**Study Sample**	**Exclusions**	**Comparison**	**Outcomes**	**Analysis**	**Results – absolute numbers**	**Results – analysis presented**	**Adjustments for confounders**	**Author's conclusion on the effect of birth location on outcomes ?****
Algert et al., 2008 (12)	New South Wales, Australia	Infants born with gastroschisis during 2001-2003	62 infants: 11 transferred to a surgical centre	Diagnosis made >28days after birth Infants with antenatally diagnosable and lethal uncorrectable defects such as anencephaly, renal agenesis and trisomy 13 or 18	Co-located hospital – not requiring neonatal emergency transport compared with Other hospital – requiring transport	1-year Mortality	Test of Proportion	Transfer: 0/11 died<br>No transfer: 7/51 died	Non-significant difference	Nil	No difference
Dalton et al., 2017 (25)	Kansas, USA	Infants born with gastroschisis during 2010-2015 ς	79 infants total: 18 transferred to surgical centre (outborn)	Complicated gastroschisis diagnosis	Inborn vs outborn	Length of stay†<br><br>Days to first feed	Descriptive Fisher's exact test	Transfer: 40+/- 26 days<br>No transfer: 30+/- 15 days<br><br>Transfer: 15+/-8.4 days<br>No transfer: 13+/-8.5 days	Group difference: P= 0.03<br><br>Group difference: P=0.17	Nil	Favour birth in surgical centre/ regionalisation of services

Confidential: For Review Only

						Days to full feed<br><br>Total Parenteral Nutrition days		Transfer: 36+/- 23 days No transfer: 16 +/- 54 days<br><br>Transfer: 32+/- 23 days No transfer: 23+/- 12 days	Group difference: P=0.07<br><br>Group difference: P= 0.03		
Hong et al., 2019 (26)	USA: Vermont Oxford Network	Infants >1500g born with gastroschisis during 2009-2015 ς	4663 infants total: 1134 transferred (outborn)	Infants transferred after repair	Inborn – birth in surgical centre vs outborn – delivery outside of centre	Mortality<br><br>Length of stay†	Multivariate logistic regression	Transfer: 23/1134 died No transfer: 67/3529 died<br><br>Transfer: 38 (27,64) days No transfer: 35 (26,55) days	aOR: 1.02 (0.60-1.72) P>0.05<br><br>Poisson regression: 0.10 (0.04,0.16) P<0.05	Clustering within hospitals	No difference
Kandasamy et al., 2009 (13)	North Queensland, Australia	Infants born with gastroschisis during 1988-2007 ς	50 infants total: 10 transferred (outborn)	Nil	Inborn vs outborn	Mortality<br><br>Length of stay	Chi-square test	No numerical data available – No difference between groups found			No difference
Kitchanan et al., 2000 (14)	North Queensland, Australia	Infants born with gastroschisis during 1988-1997ς	21 infants (inborn): 4 transferred (outborn)	Nil	Inborn – delivered in regional tertiary unit vs outborn – delivered in peripheral hospitals	Days to first feed<br><br>Days to full enteral feeding<br><br>Total Parenteral Nutrition days	Descriptive Non-parametric test	Transfer: 25 days No transfer: 9 days<br><br>Transfer: 19 days No transfer: 16 days<br><br>Transfer: 42 days No transfer: 14 days	Group difference: P=0.012<br><br>Group difference: P= 0.02<br><br>Group difference: P=0.012		No difference
Nasr and Langer, 2012 (16)	Canada	Infants born with gastroschisis during 2005-2008	395 infants: 158 transferred	Postnatal diagnosis	Inborn: born in the same building as NICU vs outborn requiring an	Mortality<br><br>Length of	Descriptive Logistic regression	Transfer: 4/158 died No transfer: 11/237 died<br><br>Transfer: 49 (27,62)	Group difference: P=0.10<br><br>Group difference:	Possible case-mix – not specified	No difference

Confidential: For Review Only

					ambulance transfer	stay†<br><br>Days to first feed<br><br>Total Parental Nutrition days<br><br>Complications		days<br>No transfer: 48 (21,54) days<br><br>Transfer: 12 (6,14) days<br>No transfer: 12 (7,15) days<br><br>Transfer: 42 (22,40) days<br>No transfer: 36 (17,40) days<br><br>Transfer: 4/158<br>No transfer: 11/237	P=0.80<br><br>Group difference: P=0.85<br><br>Group difference: P= 0.19<br><br>Group difference: P=0.10<br>OR 1.60 (1.09-2.7), P=0.05		
Savoie et al., 2014 (27)	Paediatric Surgery Research Collaborative centres, USA: Memphis, Houston, Nashville, Little Rock, Indianapolis, Kansas	Infants with gastroschisis during 2008-2013	524 infants total: 239 transferred outborn	Nil	Inborn-delivery in hospital where surgical repair occurred vs outborn-required transfer to surgical centre for repair	30-day Mortality<br><br>Length of stay†<br><br>Days to first enteral feed<br><br>Days to full enteral feeding<br><br>Total Parenteral Nutrition days	Descriptive<br>T-test<br>Chi-square<br>Multivariable analyses	Transfer: 4/239 died<br>No transfer: 4/283 died<br><br>Transfer: 42 (28,76) days<br>No transfer: 34 (24,63) days<br><br>Transfer: 18 (13,26) days<br>No transfer: 16 (11,22) days<br><br>Transfer: 31 (23,57) days<br>No transfer: 27 (20,43) days<br><br>Transfer: 29 (20,54)	Group difference: P=0.81<br><br>Group difference: P=0.002<br><br>Group difference: P=0.004<br><br>Group difference: P=0.0008<br><br>Group difference: P=0.06	Possible confounders – not specified	Favour birth in surgical centre/ regionalisation of services

Confidential: For Review Only

								days<br>No transfer:<br>27 (17,445)<br>days			

Confidential: For Review Only

Supplementary Table 1b: Summary of studies on gastroschisis included.

*not all studies provided statistical analysis i.e., ORs, some studies only described the number of events

**not all authors provided conclusions on favoured birth location

RR- Risk Ration, aRR- adjusted Risk Ratio, OR – Odds Ratio ,aOR – adjusted Odds Ratio, P- p-value, HR hazard ratio, aHR – adjusted Hazard Ratio

† Length of stay given as median (IQR)

ς Potentially overlapping cohort: multicentre or population-based studies may include duplicates infants

**OESOPHAGEAL ATRESIA / TRACHEOESOPHAGEAL FISTULA**											
**Author/Year**	**Setting**	**Population**	**Study Sample**	**Exclusions**	**Compairson**	**Outcomes**	**Analysis**	**Results – absolute numbers**	**Results – analysis presented**	**Adjustments for confounders**	**Author's conclusion on the effect of birth location on outcomes ?****
Algert et al., 2008 (12)	New South Wales, Australia	Infants with oesophageal atresia during 2001-2003	42 infants: 25 transferred	Diagnosis made >28days after birth<br>Antenatally diagnosable and lethal uncorrectable defects such as anencephaly, renal agenesis and trisomy 13 or 18	Co-located hospital – not requiring neonatal emergency transport compared with 'Other hospital' – requiring transport	1-year Mortality	Test of Proportion	Transfer:<br>1/25 died<br>No transfer:<br>3/17 died	Non-significant difference between groups	Nil	No differences
Schlee et al., 2022 (19)	Frankfurt, Germany	Infants with oesophageal atresia during 2009-2013	57 infants total: 35 transferred (outborn)	Nil	Inborn- born in institution vs outborn – referred postnatally and transported for surgery	1-year mortality<br><br>Length of stay†<br><br>Days to full enteral feeding	Descriptive<br>Mann-Whitney U test<br>Fisher's exact test	Transfer:<br>0/35 died<br>No transfer:<br>2/17 died<br><br>Transfer:<br>17 (13,36) days<br>No transfer:<br>92 (18,158) days<br><br>Transfer:<br>8 (6,11) days<br>No transfer:<br>12 (7,18) days	Group Difference:<br>P= 0.103<br><br>Group difference:<br>P= 0.009<br><br>Group difference:<br>P=0.137	Nil	No difference

Wang et al., 2014 (28)	USA: Kids' Inpatient Database	Infants with oesophageal atresia/trachoesophageal fistula during 1997-2009	4168 infants total: 1169 transferred (outborn)	Nil	Divided by admission source: Birth vs Hospital transfer	Mortality	Descriptive Fisher's exact test	Transfer: 99/1169 died No transfer: 186/1850 died	Group difference: P=0.399	Nil	No difference

Supplementary Table 1c: Summary of studies on TOF/OA included.

*not all studies provided statistical analysis i.e., ORs, some studies only described the number of events

**not all authors provided conclusions on favoured birth location

RR- Risk Ration, aRR- adjusted Risk Ratio, OR – Odds Ratio ,aOR – adjusted Odds Ratio, P- p-value, HR hazard ratio, aHR – adjusted Hazard Ratio

† Length of stay given as median (IQR)

ς Potentially overlapping cohort: multicentre or population-based studies may include duplicates infants

Confidential: For Review Only

Supplementary Material 1:

Table S1: Example Search Strategy

Search string	Database or further sources	Results	Date	Comments
((gastroschisis) OR (oesophageal atresia) OR (congenital diaphragmatic hernia)) AND ((location) OR (birth place) OR (site of delivery)) OR (transfer) OR (inborn) OR (outborn)	MEDLINE	192	2022-05-06	
MM "Gastroschisis") OR "gastroschisis" OR congenital diaphragmatic hernia OR congenital diaphragmal hernia OR tracheoesophageal fistula OR esophageal atresia AND ((location) OR (birth place) OR (site of delivery)) OR (transfer) OR (inborn) OR (outborn)	CINAHL	97	2022-05-06	
(((((ALL=(location)) OR ALL=(birthplace)) OR ALL=(site of delivery)) OR ALL=(inborn)) OR ALL=(outworn)) OR ALL=(transfer) AND (((ALL=(gastroschisis)) OR ALL=(congenital diaphragmatic hernia)) OR ALL=(tracheoesophageal fistula)) OR ALL=(esophageal atresia)	Web of Science	481	2022-05-06	
TITLE-ABS-KEY (((gastroschisis) OR (oesophageal AND atresia) OR (congenital AND diaphragmatic AND hernia)) AND ((location) OR (birth AND place) OR (site AND of AND delivery)) OR (transfer) OR (inborn) OR (outborn))	SCOPUS	529	2022-05-06	
((gastroschisis) OR (oesophageal atresia) OR (congenital diaphragmatic hernia)) AND ((location) OR (birth place) OR (site of delivery)) OR (transfer) OR (inborn) OR (outborn)	MEDLINE	10	2023-01-10	
(((((ALL=(location)) OR ALL=(birthplace)) OR ALL=(site of delivery)) OR ALL=(inborn)) OR ALL=(outworn)) OR ALL=(transfer) AND (((ALL=(gastroschisis)) OR ALL=(congenital diaphragmatic hernia)) OR ALL=(tracheoesophageal fistula)) OR ALL=(esophageal atresia)	Web of Science	35	2023-01-11	
TITLE-ABS-KEY (((gastroschisis) OR (oesophageal AND atresia) OR (congenital AND diaphragmatic AND hernia)) AND ((location) OR (birth AND place) OR (site AND of AND delivery)) OR (transfer) OR (inborn) OR (outborn))	SCOPUS	45	2023-01-11	
MM "Gastroschisis") OR "gastroschisis" OR congenital diaphragmatic hernia OR congenital diaphragmal hernia OR tracheoesophageal fistula OR esophageal atresia AND ((location) OR (birth place) OR (site of delivery)) OR (transfer) OR (inborn) OR (outborn)	CINAHL	9	2023-01-11	

Table S2: Excluded on Full Text Screening

Title	Author	Reason Exclusion
A single-center observational study on congenital diaphragmatic hernia: Outcome, predictors of mortality and experience from a tertiary perinatal center in Singapore.	Teo, Wan-Yee; Sriram, Bhavani; Alim, Aa Abdul; Ruan, Xucong; Rajadurai, V S	Details on whether transfer required? (Comparator);
Management and outcome of neonates with a prenatal diagnosis of esophageal atresia type A: A population-based study.	Garabedian, C; Bonnard, A; Rousseau, V; Sfeir, R; CRACMO; Drumez, E; Michaud, L; Gottrand, F; Houfflin-Debarge, V	Details on whether transfer required? (Comparator);
Survival of outborns with congenital diaphragmatic hernia: the role of protective ventilation, early presentation and transport distance: a retrospective cohort study.	Bojanic, Katarina; Pritisanac, Ena; Luetic, Tomislav; Vukovic, Jurica; Sprung, Juraj; Weingarten, Toby N; Carey, William A; Schroeder, Darrell R; Grizelj, Ruza	Details on whether transfer required? (Comparator);
[Influence of selected factors on the treatment and prognosis in newborns with gastroschisis on the basis of own experience].	Sawicka, Ewa; Wieprzowski, Lukasz; Jaczynska, Renata; Maciejewski, Tomasz	Details on whether transfer required? (Comparator);
Effect of time to surgical evaluation on the outcomes of infants with gastroschisis.	Bucher, Brian T; Mazotas, Ioanna G; Warner, Brad W; Saito, Jacqueline M	Details on whether transfer required? (Comparator);
Gastroschisis: a multi-centre comparison of management and outcome.	Manson, Joanna; Ameh, Emmanuel; Canvassar, Noel; Chen, Tiffany; den Hoeve, A Van; Lever, F; Hesse, Afua; Millar, Alastair; Emil, Sherif; Ade-Ajayi, Niyi	Details on whether transfer required? (Comparator);
[Congenital gastroschisis--prenatal diagnosis and perinatal management].	Weichert, J; Kahl, F O; Schroer, A; Bohlmann, M K; Diedrich, K; Hartge, D R	Details on whether transfer required? (Comparator);
Management and outcome for babies born with a gastroschisis.	Williamson, Sarah Louise; Lawrence, Louise; Arul, G. Suren; Rasiah, Shree Vishna	Details on exposure?

Confidential: For Review Only

Study of 24 cases with congenital esophageal atresia: what are the risk factors?	Sugito K; Koshinaga T; Hoshino M; Inoue M; Goto H; Ikeda T; Hagiwara N	Details on whether transfer required? (Comparator);
A 20-year experience on neonatal extracorporeal membrane oxygenation in a referral center.	Schaible T; Hermle D; Loersch F; Demirakca S; Reinshagen K; Varnholt V; Schaible, T; Hermle, D; Loersch, F; Demirakca, S; Reinshagen, K; Varnholt, V	Details on whether transfer required? (Comparator);
Predictive factors for complications in children with esophageal atresia and tracheoesophageal fistula	Shah, R; Varjavandi, V; Krishnan, U	Details on whether transfer required? (Comparator);
Congenital diaphragmatic hernia - Results of an ECMO-centre	Dahlheim, M; Witsch, M; Demirakca, S; Lorenz, C; Schaible, T	Details on whether transfer required? (Comparator);
Congenital diaphragmatic hernia: A local experience	Khawahur, H; Kattan, A; Al-Alaiyan, S; Saidy, K	Details on whether transfer required? (Comparator);
Congenital diaphragmatic hernia: a modern day approach	Waag, KL; Loff, S; Zahn, K; Ali, M; Hien, S; Kratz, M; Neff, W; Schaffeder, R; Schaible, T	Details on whether transfer required? (Comparator);
Outcomes of Congenital Diaphragmatic Hernia: An 8-Year Experience	Fallahi, M; Mohajerzadeh, L; Borhani, S; Kazemian, M; Roozroukh, M; Khaleghnejad-Tabari, A; Azma, R; Mahdavi, A	Details on whether transfer required? (Comparator);
Outcomes of newborns with gastroschisis: The effects of mode of delivery, site of delivery, and interval from birth to surgery	Quirk, JG; Fortney, J; Collins, HB; West, J; Hassad, SJ; Wagner, C	
Congenital diaphragmatic hernia - a Belgrade single center experience	Kalanj, J; Salevic, P; Rsovac, S; Medjo, B; Antunovic, SS; Simic, D	Details on whether transfer required? (Comparator);
Congenital diaphragmatic hernia: a survey of practice in Scandinavia	Skari, H; Bjornland, K; Frenckner, B; Friberg, LG; Heikkinen, M; Hurme, T; Loe, B; Mollerlokken, G; Nielsen, OH; Qvist, N; Rintala, R; Sandgren, K; Serlo, W; Wagner, K; Wester, T; Emblem, R	Details on whether transfer required? (Comparator);
Survival of outborns with congenital diaphragmatic hernia: the role of protective ventilation, early presentation and transport distance: a retrospective cohort study	Bojanic, K; Pritisanac, E; Luetic, T; Vukovic, J; Sprung, J; Weingarten, TN; Carey, WA; Schroeder, DR; Grizelj, R	Details on whether transfer required? (Comparator);
A population-based study of congenital diaphragmatic hernia: Impact of associated anomalies and preoperative blood gases on survival	Kaiser, JR; Rosenfeld, CR	Details on whether transfer required? (Comparator);
Outcomes of congenital diaphragmatic hernia: A population-based study in western Australia	Colvin, J; Bower, C; Dickinson, JE; Sokol, J	Details on whether transfer required? (Comparator);
Outcomes in the physiologically most severe congenital diaphragmatic hernia (CDH) patients: Whom should we treat?	Kays, DW; Islam, S; Perkins, JM; Larson, SD; Taylor, JA; Talbert, JL	Details on whether transfer required? (Comparator);
Congenital diaphragmatic hernia: outcome review of 2,173 surgical repairs in US infants	Abdullah, F; Zhang, YY; Sciortino, C; Camp, M; Gabre-Kidan, A; Price, MR; Chang, DC	Details on whether transfer required? (Comparator);
Congenital Diaphragmatic Hernia Defect Size and Infant Morbidity at Discharge	Putnam, LR; Harting, MT; Tsao, K; Morini, F; Yoder, BA; Luco, M; Lally, PA; Lally, KP	Details on whether transfer required? (Comparator);
Analysis of 29 consecutive thoracoscopic repairs of congenital diaphragmatic hernia in neonates compared to historical controls	Cho, SD; Krishnaswami, S; Mckee, JC; Zallen, G; Silen, ML; Bliss, DW	Details on whether transfer required? (Comparator);
CONGENITAL DIAPHRAGMATIC-HERNIA - UPDATE ON REGIONAL EXPERIENCE	ALUMRAN, K; KHAWAJA, S; DAWODU, AH; ALARFJ, A	Details on whether transfer required? (Comparator);
IS SPECIALIST CENTER DELIVERY OF GASTROSCHISIS BENEFICIAL	NICHOLLS, G; UPADHYAYA, V; GORNALL, P; BUICK, RG; CORKERY, JJ	Details on whether transfer required? (Comparator);
Practice variation in gastroschisis: Factors influencing closure technique	Stanger, J; Mohajerani, N; Skarsgard, ED	Details on whether transfer required? (Comparator);
Delivery in a tertiary Center with co-located surgical facilities makes the difference among neonates with prenatally diagnosed major abnormalities	Calisti, A; Oriolo, L; Giannino, G; Spagnol, L; Molle, P; Buffone, EL; Donadio, C	Known birth location and whether surgical services are available on site (Intervention/Exposure)
INFLUENCE OF PLACE OF DELIVERY ON OUTCOME IN BABIES WITH GASTROSCHISIS	STOODLEY, N; SHARMA, A; NOBLETT, H; JAMES, D	Known birth location and whether surgical services are available on site (Intervention/Exposure)
Gastroschisis outcomes and site of delivery	Robilio, D; Greve, L; Towner, D	Known birth location and whether surgical services are available on site

Confidential: For Review Only

		(Intervention/Exposure)
Mode of delivery and neonatal survival of infants with gastroschisis in Australia and New Zealand	Abdel-Latif, ME; Bolisetty, S; Abeywardana, S; Lui, K	Details on whether transfer required? (Comparator);
Factors Associated with Mortality in Neonates with Gastroschisis	Clark, RH; Walker, MW; Gauderer, MWL	Known birth location and whether surgical services are available on site (Intervention/Exposure)
Perinatal demography of gastroschisis in North Queensland	Whitehall, JS; Kandasamy, Y; Stalewski, H; Gill, A	Details on whether transfer required? (Comparator);
An Investigation of Racial-Ethnic Disparities in Location of Delivery, Hospital Transfer, and Survival Among Infants with Congenital Diaphragmatic Hernia	Carmichael, SL; Ma, C; Shaw, GM; Sylvester, KG; Hintz, SR	Known birth location and whether surgical services are available on site (Intervention/Exposure)
Gastroschisis and exomphalos in Ireland 1998-2004. Does antenatal diagnosis impact on outcome?	Murphy, FL; Mazlan, TA; Tarheen, F; Corbally, MT; Puri, P	Details on whether transfer required? (Comparator);
Trends in incidence and outcomes of gastroschisis in the United States: analysis of the national inpatient sample 2010-2014	Bhatt, P; Lekshminarayanan, A; Donda, K; Dapaah-Siakwan, F; Thakkar, B; Parat, S; Chabra, S; Billimoria, Z	Details on whether transfer required? (Comparator);
Complications of esophageal strictures dilatation in children A tertiary-center experience	Bawazir, O; Almaimani, MO	Details on whether transfer required? (Comparator);
The Effect of Level of Care on Gastroschisis Outcomes	Apfeld, JC; Kastenberg, ZJ; Sylvester, KG; Lee, HC	Details on whether transfer required? (Comparator);
Outcomes of newborns with gastroschisis: The effects of mode of delivery, site of delivery, and interval from birth to surgery - Discussion	Niebyl, JR; Woods, JR; Evans, MI; Queenan, JT; Carpenter, MW; Merkatz, IR; Quirk	Details on whether transfer required? (Comparator);
CONTROVERSIES IN THE MANAGEMENT OF GASTROSCHISIS - A STUDY OF 40 PATIENTS	STRINGER, MD; BRERETON, RJ; WRIGHT, VM	Details on whether transfer required? (Comparator);
Length of stay and cost analysis of neonates undergoing surgery at a tertiary neonatal unit in England	Shetty, S; Kennea, N; Desai, P; Giuliani, S; Richards, J	Details on whether transfer required? (Comparator);
Considerations for delivery of infants with congenital abnormalities	Wilkins-Haug, L	Details on whether transfer required? (Comparator);
Timing and location of delivery of the fetus with gastroschisis	Leuthner, SR; Wigton, T; Brown, AM; Sweeney, B; Sato, T; Aiken, J	Details on whether transfer required? (Comparator);
A systematic review and meta-analysis of the impact of location of delivery on outcomes in infants with gastroschisis	Nitsche, JF; Li, Y; Brost, B	Details on whether transfer required? (Comparator);
Prenatal diagnosis and the pediatric surgeon: The impact of prenatal consultation on perinatal management	Crombleholme, TM; DAlton, M; Cendron, M; Alman, B; Goldberg, MD; Klauber, GT; Cohen, A; Heilman, C; Lewis, M; Harris, BH	Details on whether transfer required? (Comparator);
Transfer and treatment mechanism and practical effect analysis of prenatally diagnosed congenital dia-phragmatic hernia	Ying, W.; Lishan, M.; Chao, L.; Yandong, W.; Jingna, L.; Yunlong, Z.; Yanxia, Z.; Rui, Z.	Details on whether transfer required? (Comparator);
The financial burden of surgery for congenital malformations—the austrian perspective	Gasparella, P.; Singer, G.; Kienesberger, B.; Arneitz, C.; Fülöp, G.; Castellani, C.; Till, H.; Schalamon, J.	Details on whether transfer required? (Comparator);
International recommendations on neonatal management with congenital diaphragmatic hernia: literature review	Babintseva, A.G.; Godovanets, Yu.D.; Khodzinska, Yu.Yu.; Popeliuk, N.O.; Koshurba, I.V.	Details on whether transfer required? (Comparator);
Clinical experience of ex-utero intrapartum treatment and prognostic factors of neonatal diaphragmatic hernia	Yunlong, Z.; Lishuang, M.; Yidin, J.; Ying, W.; Chao, L.; Yandong, W.; Jingna, L.; Yan, L.; Shan, J.; Hua, M.; Yunshu, O.; Feng, F.; Huf, Y.; Lijian, P.	Details on whether transfer required? (Comparator);
Effect of multidisciplinary treatment on outcomes in infants with congenital diaphragmatic hernia: 10-year experience	Pan, W.; Wang, W.; Wang, J.; Xie, W.; Wang, Y.; Jiang, Y.	Details on whether transfer required? (Comparator);
Prenatal diagnoses and outcomes of congenital diaphragmatic hernia.	Laye, M.R.; Rehberg, J.F.; Kosek, M.A.; Bufkin, L.K.; Bofill, J.A.	Details on whether transfer required? (Comparator);
Risk factors for mortality of newborns with gastroschisis in a tertiary hospital of Taiwan	Zhao, L.-L.; Chang, C.-J.; Chou, H.-C.; Tsao, P.-N.; Hsieh, W.-S.	Details on whether transfer required? (Comparator);
Gastroschisis: Determinants of neonatal outcome	Singh, S.J.; Fraser, A.; Leditschke, J.F.; Spence, K.; Kimble, R.; Dalby-Payne, J.;	Details on whether transfer required? (Comparator);

	Baskaranathan, S.; Barr, P.; Halliday, R.; Badawi, N.; Peat, J.K.; Glasson, M.; Cass, D.	
Congenital diaphragmatic hernia. Prenatal diagnosis and neonatal outcome	Lamberti, A.; Liguori, M.; Teodoro, A.; Tartaglione, A.; Caccioppoli, U.; Paladini, D.	Known birth location and whether surgical services are available on site (Intervention/Exposure)
Current status of antenatal diagnosis & perinatal medical network	Suita, S.	Details on whether transfer required? (Comparator);
Exomphalos and gastroschisis, a study of fetal and neonatal outcome in Wales 1974-1987	Evans, J.; Madarikan, B.A.; Lari, J.	Details on whether transfer required? (Comparator);
Anterior abdominal wall defects	Lafferty, P.M.; Emmerson, A.J.; Fleming, P.J.; Frank, J.D.; Noblett, H.R.	Known birth location and whether surgical services are available on site (Intervention/Exposure)
Development of a program for planned cesarean delivery and immediate pediatric surgical repair of ventral wall defects	Evans, M.I.; Drugan, A.; Greenholz, S.K.; Hauff, N.; Jewell, M.R.; Klein, M.D.	Details on whether transfer required? (Comparator);
Congenital diaphragmatic hernia: a 20 year experience	Simson, J.N.L.; Eckstein, H.B.	Details on whether transfer required? (Comparator);
Prenatal diagnosis and natural history of the fetus with a congenital diaphragmatic hernia: Initial clinical experience	Nakayama, D.K.; Harrison, M.R.; Chinn, D.H.; Callen, P.W.; Filly, R.A.; Golbus, M.S.; De Lorimier, A.A.	Known birth location and whether surgical services are available on site (Intervention/Exposure)
Perinatal management of gastroschisis	Fitzsimmons, J.; Nyberg, D. A.; Cyr, D. R.; Hatch, E.	Known birth location and whether surgical services are available on site (Intervention/Exposure)
Clinical profile, outcomes and predictors of mortality in neonates operated for gastrointestinal anomalies in a tertiary neonatal care unit- An observational study	Jerry, A.L.; Amboiram, P.; Balakrishnan, U.; Chandrasekaran, A.; Agarwal, P.; Devi, U.	Known birth location and whether surgical services are available on site (Intervention/Exposure)
Prenatal diagnoses and outcomes of congenital diaphragmatic hernia.	Laye, M.R.; Rehberg, J.F.; Kosek, M.A.; Bufkin, L.K.; Bofill, J.A.	Known birth location and whether surgical services are available on site (Intervention/Exposure)
Risk factors for mortality of newborns with gastroschisis in a tertiary hospital of Taiwan	Zhao, L.-L.; Chang, C.-J.; Chou, H.-C.; Tsao, P.-N.; Hsieh, W.-S.	Known birth location and whether surgical services are available on site (Intervention/Exposure)
Risk of need for extracorporeal membrane oxygenation support in neonates with congenital diaphragmatic hernia treated with inhaled nitric oxide	Sebald, M.; Friedlich, P.; Burns, C.; Stein, J.; Noori, S.; Ramanathan, R.; Seri, I.	Known birth location and whether surgical services are available on site (Intervention/Exposure)
Gastroschisis: Determinants of neonatal outcome	Singh, S.J.; Fraser, A.; Leditschke, J.F.; Spence, K.; Kimble, R.; Dalby-Payne, J.; Baskaranathan, S.; Barr, P.; Halliday, R.; Badawi, N.; Peat, J.K.; Glasson, M.; Cass, D.	Known birth location and whether surgical services are available on site (Intervention/Exposure)
Congenital diaphragmatic hernia. Prenatal diagnosis and neonatal outcome	Lamberti, A.; Liguori, M.; Teodoro, A.; Tartaglione, A.; Caccioppoli, U.; Paladini, D.	Known birth location and whether surgical services are available on site (Intervention/Exposure)
Current status of antenatal diagnosis & perinatal medical network	Suita, S.	Details on whether transfer required? (Comparator);
Exomphalos and gastroschisis, a study of fetal and neonatal outcome in Wales 1974-1987	Evans, J.; Madarikan, B.A.; Lari, J.	Details on whether transfer required? (Comparator);
Anterior abdominal wall defects	Lafferty, P.M.; Emmerson, A.J.; Fleming, P.J.; Frank, J.D.; Noblett, H.R.	Known birth location and whether surgical services are available on site (Intervention/Exposure)
Development of a program for planned cesarean delivery and immediate pediatric surgical repair of ventral wall defects	Evans, M.I.; Drugan, A.; Greenholz, S.K.; Hauff, N.; Jewell, M.R.; Klein, M.D.	Details on whether transfer required? (Comparator);
Congenital diaphragmatic hernia: a 20 year experience	Simson, J.N.L.; Eckstein, H.B.	Details on whether transfer required? (Comparator);
Prenatal diagnosis and natural history of the fetus with a congenital diaphragmatic hernia: Initial clinical experience	Nakayama, D.K.; Harrison, M.R.; Chinn, D.H.; Callen, P.W.; Filly, R.A.; Golbus, M.S.; De Lorimier, A.A.	Details on whether transfer required? (Comparator);
Perinatal management of gastroschisis	Fitzsimmons, J.; Nyberg, D. A.; Cyr, D. R.; Hatch, E.	Details on whether transfer required? (Comparator);
Clinical profile, outcomes and predictors of mortality in neonates operated for gastrointestinal anomalies in a tertiary neonatal care unit- An observational study	Jerry, A.L.; Amboiram, P.; Balakrishnan, U.; Chandrasekaran, A.; Agarwal, P.; Devi, U.	Details on whether transfer required? (Comparator);

Confidential: For Review Only

Confidential: For Review Only
